# Reconstitution of glycan-driven MHC-I recycling reveals calreticulin as mediator between TAPBPR and tapasin

**DOI:** 10.1038/s44318-026-00873-2

**Published:** 2026-08-17

**Authors:** Tim J Heinke, Amin Fahim, Niko Popovic, Tobias Rath, Ines K Müller, Nina Morgner, Simon Trowitzsch, Robert Tampé

**Affiliations:** 1 Institute of Biochemistry, Biocenter, Goethe University Frankfurt, Max-von-Laue-Str. 9, 60438 Frankfurt am Main, Germany; 2 Institute of Physical and Theoretical Chemistry, Goethe University Frankfurt, Max-von-Laue-Str. 9, 60438 Frankfurt am Main, Germany

**Keywords:** Immunology, Translation & Protein Quality

## Abstract

Protein folding in the endoplasmic reticulum (ER) relies on N-linked glycosylation and glycan remodeling to guide quality control. Major histocompatibility complex class I (MHC-I) molecules, essential for adaptive immunity, undergo a specialized maturation pathway involving the peptide-loading complex (PLC), the editor TAPBPR, the UDP-glucose:glycoprotein glucosyltransferase, and the lectin chaperone calreticulin. However, how glycan-dependent mechanisms coordinate MHC-I transfer between these factors has remained unclear. Using a fully reconstituted system, we show that retrograde transfer of peptide-receptive MHC-I from TAPBPR to tapasin requires calreticulin recognition of monoglucosylated MHC-I glycans. While calreticulin’s C-terminal acidic helix is dispensable for releasing reglucosylated MHC-I from TAPBPR, it is essential for productive docking of MHC-I onto tapasin. These findings reveal a glycan-surveillance mechanism that enables retrieval of suboptimally loaded MHC-I molecules missed by the initial quality control at the PLC. Our work defines a glycan-dependent chaperone network, finely tuned by a combination of low-micromolar interactions between the constituents, that ensures efficient MHC-I maturation and illustrates fundamental principles of ER protein quality control.

## Synopsis

Major histocompatibility complex class I (MHC-I) molecules undergo a specialized maturation pathway, but the mechanisms that coordinate MHC-I transfer between its components are incompletely understood. This study uses a fully reconstituted ER quality control system to demonstrate that calreticulin drives the glycan-dependent recycling of peptide-receptive MHC-I molecules from TAPBPR to the peptide-loading complex.

- This study reconstitutes a minimal MHC-I ER quality control pathway from purified recombinant MHC-I, TAPBPR, UGGT, calreticulin, and tapasin–ERp57 components.
- UGGT-dependent monoglucosylation of peptide-receptive MHC-I is required for its transfer from TAPBPR to the peptide-loading complex.
- Calreticulin selectively binds monoglucosylated MHC-I and forms a transient intermediate that mediates handover between MHC-I quality control checkpoints.
- The acidic C-terminal helix of calreticulin is dispensable for MHC-I capture but essential for transfer of MHC-I to tapasin–ERp57.
- These findings define the molecular basis of a key quality control step in MHC-I antigen presentation.

## Abstract (web summary)

Calreticulin binds monoglucosylated MHC-I to mediate its handover between MHC-I quality control checkpoints.

## Graphical abstract

**Figure:**
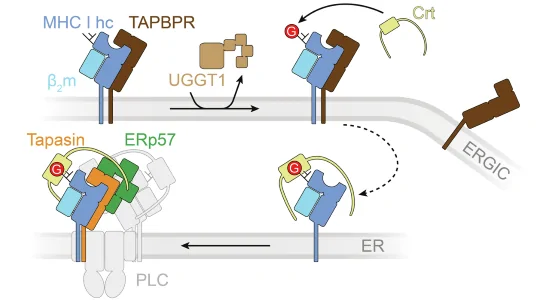

**Figure:**
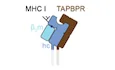

## Introduction

Protein folding and quality control (QC) in the endoplasmic reticulum (ER) govern the maturation of approximately one-third of the mammalian proteome, including membrane-associated, secreted, and organelle-targeted proteins (Gemmer et al, 2023; Guay et al, 2025). Nascent polypeptides entering the ER via signal peptide-mediated translocation frequently receive N-linked glycans from the oligosaccharyltransferase (OST), which attaches a Glc_3_Man_9_GlcNAc_2_ glycan to asparagine residues within Asn-X-Ser/Thr motifs (Breitling and Aebi, 2013). Subsequent glucose trimming by ER-resident glycosidases generates specialized glycoforms that act as maturation barcodes interpreted by lectin-like chaperones (Barker and Rose, 2013; Caputo et al, 2016; Helenius and Aebi, 2001). Glucosidase II plays a central role in this glycan cycle by sequentially removing the two innermost glucose residues. This process generates initially monoglucosylated and subsequently fully deglucosylated glycoforms, which mediate entry into the calnexin/calreticulin (Cnx/Crt) cycle as well as release from it (Deprez et al, 2005; Helenius and Aebi, 2004). Another key regulator of this process is UDP-glucose:glycoprotein glucosyltransferase 1 (UGGT1), which selectively reglucosylates non-native glycoproteins, thereby prolonging their residence in the Cnx/Crt cycle until proper folding is achieved (Kozlov and Gehring, 2020; Ninagawa et al, 2024). Through this iterative trimming process, glucosidase II functionally opposes UGGT1-mediated reglucosylation and enables repeated rounds of chaperone binding, thereby serving as another driver of glycoprotein QC.

Major histocompatibility class I (MHC I) molecules are subject to this multilayered QC (Neerincx et al, 2017). MHC I heavy chains assemble with β₂-microglobulin (β_2_m) in the ER and ultimately bind peptides derived from proteasomal degradation products transported by TAP1/2 (Blum et al, 2013; Pishesha et al, 2022; Trowitzsch and Tampé, 2020). High-fidelity peptide selection requires the peptide-loading complex (PLC), a dynamic supramolecular assembly composed of TAP1/2, tapasin, ERp57, Crt, and peptide-receptive MHC I (Blees et al, 2017; Stolz et al, 2026). Tapasin and the related chaperone TAPBPR stabilize peptide-receptive MHC I and catalyze peptide exchange (Boyle et al, 2013; Chen and Bouvier, 2007; Fleischmann et al, 2015; Hermann et al, 2013; Hermann et al, 2015; Howarth et al, 2004; Morozov et al, 2016; Sagert et al, 2020; Tan et al, 2002; Wearsch and Cresswell, 2007; Williams et al, 2002). Although structurally related (Jiang et al, 2017; Jiang et al, 2022; Müller et al, 2022; Thomas and Tampé, 2017), the two chaperones operate in different ER sub-compartments: tapasin functions within the PLC, whereas TAPBPR resides outside the PLC (Boyle et al, 2013; Hermann et al, 2015; Lan et al, 2023; Neerincx et al, 2017).

Crt is an integral component of the PLC: it binds MHC I through a monoglucosylated N-glycan on Asn86, interacts through its P-domain with ERp57, and engages the ER-lumenal domain of tapasin via its acidic C-terminal helix (Blees et al, 2017; Domnick et al, 2022; Frickel et al, 2002; Kozlov et al, 2006; Wearsch et al, 2004). Lack of Crt or mutations in Crt that alter Ca²⁺ buffering, lectin activity, or ER retention can profoundly impact antigen presentation and are associated with neoplasia and altered immune surveillance (Arshad and Cresswell, 2018, 2021; Gao et al, 2002; Klampfl et al, 2013). Despite extensive knowledge on the functions of the individual chaperones, the mechanisms by which MHC I molecules are shuttled between TAPBPR and the PLC remain insufficiently understood. In particular, it is not known how MHC I molecules that fail TAPBPR-mediated peptide editing are returned to the PLC for another round of high-affinity peptide selection. Existing cellular studies (Garstka et al, 2007; Howe et al, 2009; Neerincx and Boyle, 2019; Wearsch et al, 2004; Wearsch et al, 2011) cannot resolve the sequential nature of these QC handovers due to overlapping pathways, limited temporal resolution, and the complexity of the ER environment.

To address these critical gaps, we developed a compositionally defined in vitro reconstitution system that integrates protein- and glyco-engineering to capture cooperative actions of TAPBPR, UGGT1, Crt, and tapasin-ERp57. This platform provides precise biochemical control over individual ERQC steps and, importantly, enables isolation of transient intermediates that cannot be experimentally accessed in living cells. Using this approach, we uncover how Crt orchestrates the retrograde transfer of peptide-receptive MHC I from TAPBPR to the PLC in a glycan-dependent fashion. We define specific molecular contributions of Crt’s lectin domain and C-terminal acidic helix in mediating chaperone exchange. Our results show that the acidic helix is essential for productive recruitment of MHC I to tapasin-ERp57, while it is not required for releasing MHC I from TAPBPR. This requirement aligns with published observations on a cancer-associated Crt frameshift mutant in which substitution of the acidic helix with a basic counterpart impairs MHC I maturation (Arshad and Cresswell, 2018), underscoring the functional importance of this structural element.

Our data reveal the principle of a sequential, cooperative QC network that ensures the fidelity of antigen presentation by routing suboptimally loaded MHC I molecules back to the PLC. These insights advance our mechanistic understanding of ERQC processes fundamental to adaptive immunity and provide a biochemical framework for interpreting phenotypes of disease-associated Crt mutants and MHC I allomorph-specific QC outcomes.

## Results

### Engineering glycan-driven MHC I chaperone handover in vitro

**Figure 1:**
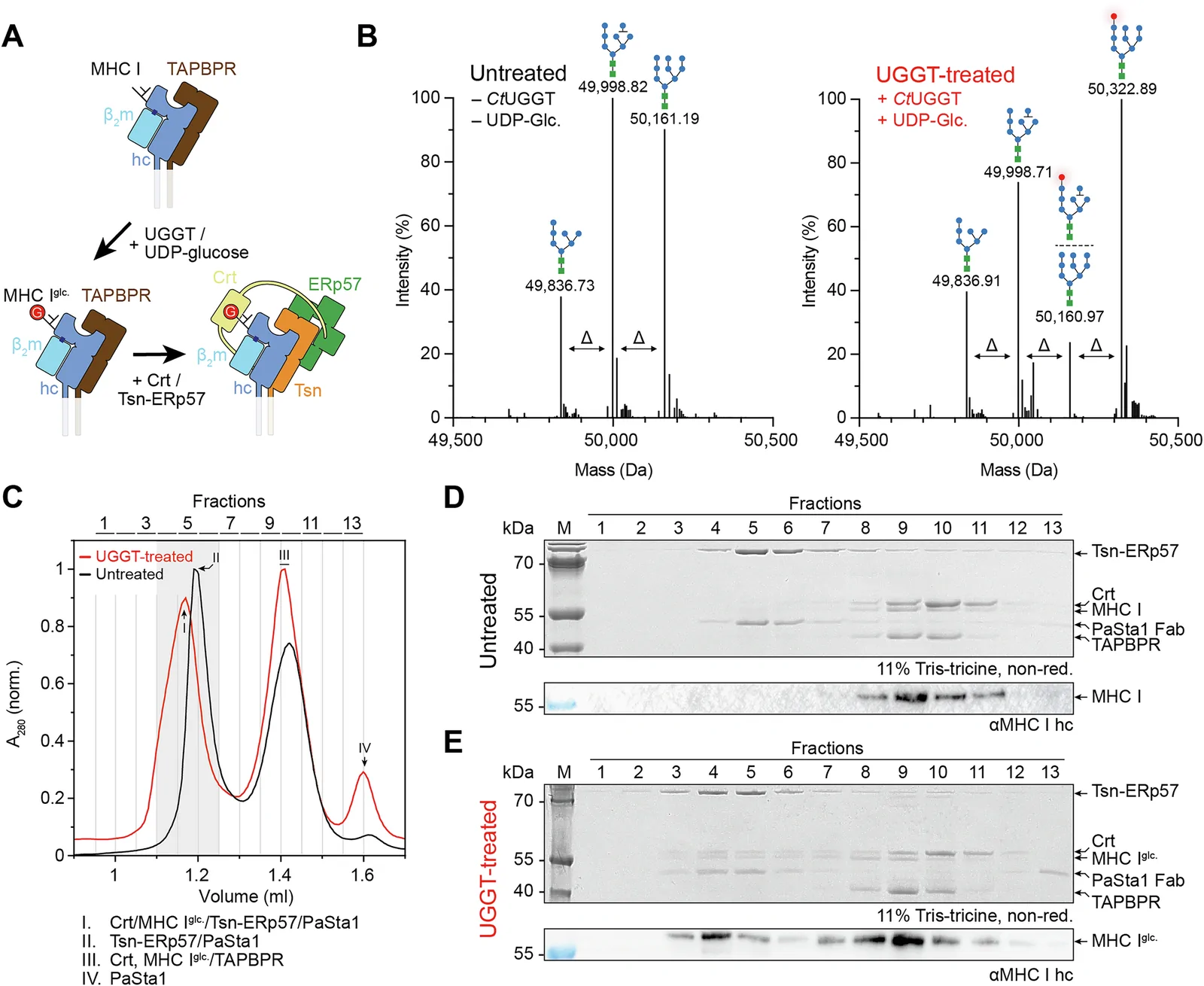
Transfer of MHC I from TAPBPR to tapasin depends on monoglucosylated glycan. (**A**) Schematic representation of the handover assay, highlighting the interaction between MHC I heavy chain (hc, blue), β_2_-microglobulin (β_2_m, cyan), TAPBPR (brown), calreticulin (Crt, yellow), ERp57 (green), and tapasin (Tsn, orange). The glucose moiety of the monoglucosylated glycan (Glc_1_Man_9_GlcNAc_2_) of the MHC I hc is marked by a red-circled G. (**B**) Deconvoluted mass spectra of single-chain β_2_m-HLA-A*68:02 displaying the native glycan pattern (untreated) and UGGT/UDP-glucose-treated single-chain β_2_m-HLA-A*68:02 (UGGT-treated). A mass shift of 162 Da (Δ), corresponding to one hexose moiety, is indicated (Δ). Peak labels show the structures of the N-glycans attached to the β_2_m-HLA-A*68:02 single-chain construct and corresponding observed masses in Dalton (green dot – GlcNAc, blue dot – Man, red dot – Glc). (**C**) Size-exclusion chromatography (SEC) analyses of β_2_m-HLA-A*68:02 containing either an unmodified Man_9_GlcNAc_2_ glycan (untreated, black) or a monoglucosylated Glc_1_Man_9_GlcNAc_2_ glycan (UGGT-treated, red) in the presence of tapasin-ERp57/PaSta1, TAPBPR, and Crt (3 µM each). Gray region indicates the differences in migration range of tapasin-ERp57/PaSta1 and Crt/MHC I^glc.^/tapasin-ERp57/PaSta1 complexes. Roman numbers above each peak relate to proteins or protein complexes defined below. (**D**,** E**) Non-reducing SDS-PAGE and immunoblot analysis of SEC fractions from untreated (**D**) or UGGT-treated (**E**) single-chain β_2_m-HLA-A*68:02 using the HC10 antibody against MHC I heavy chain. The depicted fractions correspond to an SEC elution volume range of 0.95–1.60 mL. M marker, I input, kDa kilodalton, A_280_ absorption at 280 nm; αMHC I anti-MHC I heavy chain. Data shown in panels (**C**–**E**) are representative of three independent experiments (*n* = 3). Source data are available online for this figure.

The ER folding environment is highly complex: Client proteins encounter multiple chaperones and glycan-modifying enzymes in dynamic, overlapping equilibria that pose a considerable challenge for mechanistic studies in cellulo. To resolve these steps with precision, we designed a fully reconstituted in vitro system composed of purified components. This reductionist approach allowed us to control the glycosylation state of MHC I molecules and to sequentially monitor their interactions with TAPBPR, Crt, and the tapasin-ERp57 complex. We selected the TAPBPR-dependent, yet tapasin-independent, MHC I allomorph HLA-A*68:02 (Bashirova et al, 2020; Ilca et al, 2019) to enable robust detection of chaperone exchange events. To analyze UGGT1-dependent transfer of MHC I from the secondary chaperone TAPBPR to the primary MHC I chaperone tapasin in vitro (Fig. 1A), we first co-expressed and secreted a fusion construct comprising the ectodomain of HLA-A*68:02 heavy chain (hc) and β_2_m (hereafter termed MHC I), equipped with a cleavable C-terminal Fos leucine zipper motif. Additionally, we included the ER-lumenal domain of TAPBPR with a cleavable C-terminal Jun leucine zipper motif and a Twin-Strep-tag (O’Rourke et al, 2019; Sagert et al, 2023).

**Figure EV1:**
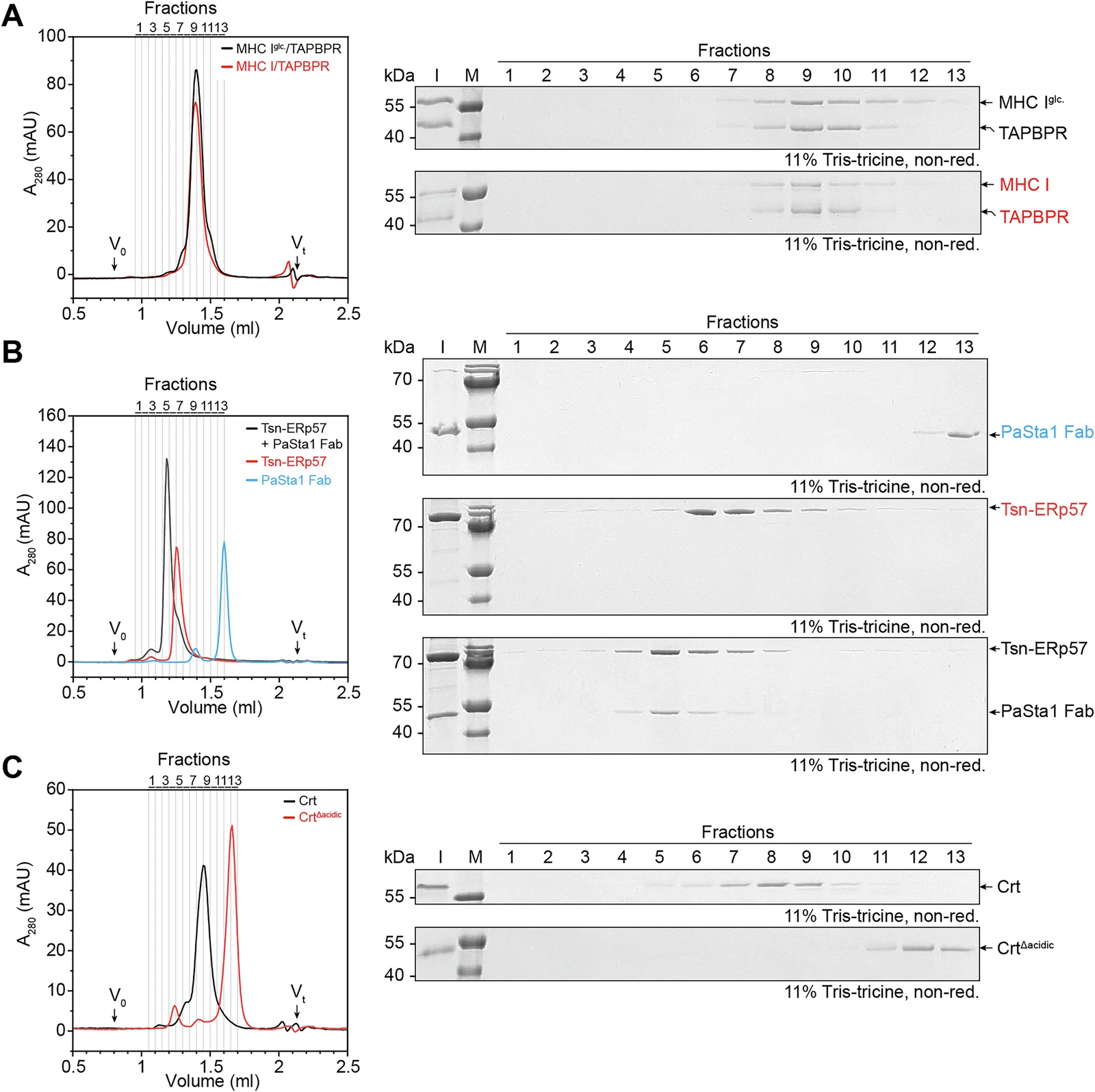
Analysis of chaperone-MHC I complexes and their individual components by SEC. (**A**) SEC profiles (left) and SDS-PAGE analyses of indicated fractions (right) for the single-chain β_2_m-HLA-A*68:02/TAPBPR complex (3 µM, black line) or the monoglucosylated complex (3 µM, red line). (**B**) SEC profiles (left) and SDS-PAGE analysis of indicated fractions (right) for PaSta1 Fab alone (3 µM, blue line), soluble tapasin-ERp57 (3 µM, red line), and the tapasin-ERp57/PaSta1 complex (3 µM, black line). (**C**) SEC profiles (left) and SDS-PAGE analysis of indicated fractions (right) for calreticulin (Crt, 3 µM, black line) and its deletion variant (Crt^Δacidic^, 3 µM, red line). M marker, I input, kDa kilodalton, A_280_ absorption at 280 nm. Data shown in panels (**A**–**C**) are representative of three independent experiments (*n* = 3). Source data are available online for this figure.

**Figure EV2:**
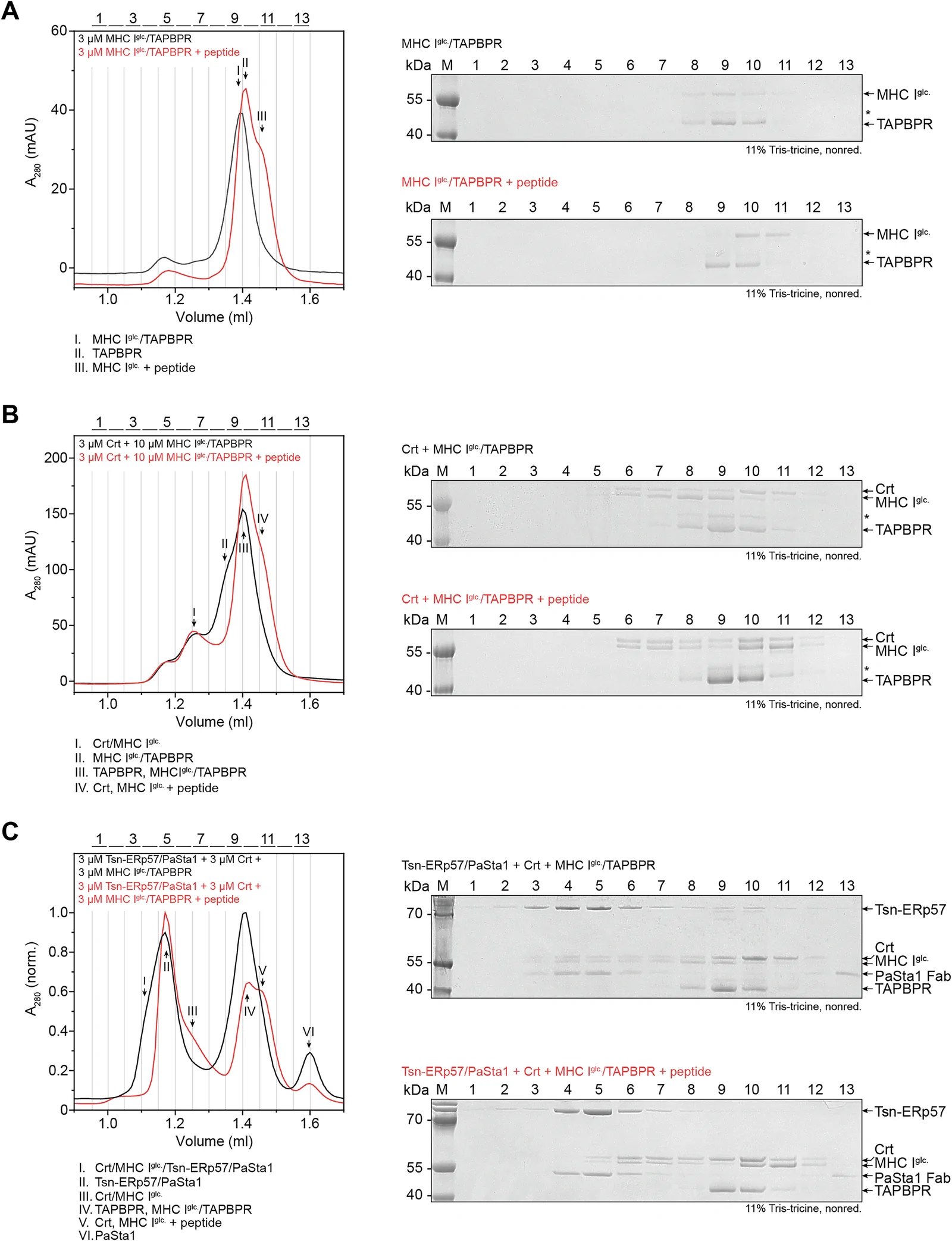
Influence of high-affinity peptides on MHC I quality control and recycling complexes. (**A**) SEC profiles (left) and SDS-PAGE analysis of indicated fractions (right) for the monoglucosylated β_2_m-HLA-A*68:02/TAPBPR complex (3 µM) in the presence (red line) or absence (black line) of peptide ETVSKQSNV (0.75 mM). (**B**) SEC profiles (left) and SDS-PAGE analysis (right) of mixtures of calreticulin (Crt, 3 µM) with monoglucosylated single-chain β_2_m-HLA-A*68:02/TAPBPR complex (10 µM) in the presence (red line) or absence (black line) of peptide ETVSKQSNV (2.5 mM). (**C**) SEC profiles (left) and SDS-PAGE analysis (right) of mixtures of tapasin-ERp57/PaSta1 Fab (3 μM), monoglucosylated single-chain β_2_m-HLA-A*68:02/TAPBPR complex, and Crt with (red line) or without (black line) peptide ETVSKQSNV (0.75 mM). Note that SEC profiles and corresponding SDS-PAGE analyses shown in panels (**B**, **C**) are identical to those in Figs. 3B, C and 1C, E, respectively. Image shown in panel (**B**) was taken from the same gel as shown in Fig. 3C, but at a different exposure time. Roman numbers above each peak correspond to the proteins or protein complexes defined below. M marker, I input, kDa kilodalton, A_280_ absorption at 280 nm. Data shown in panels (**A**–**C**) are representative of two independent experiments (*n* = 2) with peptide and three independent experiments (*n* = 3) without peptide. Source data are available online for this figure.

To generate soluble TAPBPR/MHC I heterodimers with a defined mannose-rich Man_9_GlcNAc_2_ glycan attached to Asn86 of the MHC I hc, we included the α-mannosidase I inhibitor kifunensine (Chang et al, 2007; Elbein, 1991) during expression in HEK293 cells (Sagert et al, 2023). We purified the secreted, zippered MHC I/TAPBPR complex by affinity chromatography, removed the Jun-Fos zipper motifs by Tobacco Etch Virus (TEV) protease treatment, and performed size-exclusion chromatography (SEC) to isolate the final complex (Fig. EV1A). HLA-A*68:02 remained peptide-receptive in complex with TAPBPR, as shown by dissociation of TAPBPR following incubation with high-affinity peptide ETVSKQSNV (predicted *K*_d_ ≈ 16 nM) (Andreatta and Nielsen, 2016) (Fig. EV2A), consistent with published reports (O’Rourke et al, 2019; Sagert et al, 2023).

We next produced a recombinant tapasin-ERp57 heterodimer, comprising the ER-lumenal domain of tapasin and full-length ERp57 carrying the stabilizing Cys36Ala substitution, using insect cell expression (Dong et al, 2009; Müller et al, 2022) (Fig. EV1B). To enable separation of MHC I/TAPBPR from MHC I/tapasin-ERp57 complexes by SEC, we increased the hydrodynamic radius of the tapasin-ERp57 heterodimer by attaching a Fab fragment of the well-described PaSta1 antibody (Dick et al, 2002) to tapasin at a slight molar excess (~1.2-fold) (Fig. EV1B). The PaSta1 Fab fragment binds on top of the N-IgV domain of tapasin without affecting interactions between MHC I and tapasin-ERp57 (Jiang et al, 2022).

Lastly, we isolated recombinant His-tagged Crt or a Crt variant lacking a segment of the C-terminal acidic helix (hereafter termed Crt^Δacidic^) from *E. coli* extracts by immobilized-metal-affinity chromatography followed by SEC (Fig. EV1C). We next assayed the chaperone handover of MHC I from TAPBPR to tapasin-ERp57 by assessing complex formation under competitive conditions using SEC. In parallel, we determined equilibrium dissociation constants (*K*_d_) for the critical interactions underlying this process by isothermal titration calorimetry (ITC). The glycan status of MHC I was determined by liquid chromatography coupled mass spectrometry (LC-MS) (Appendix Table S1).

### A monoglucosylated glycan of MHC I is essential for chaperone transfer

**Figure EV3:**
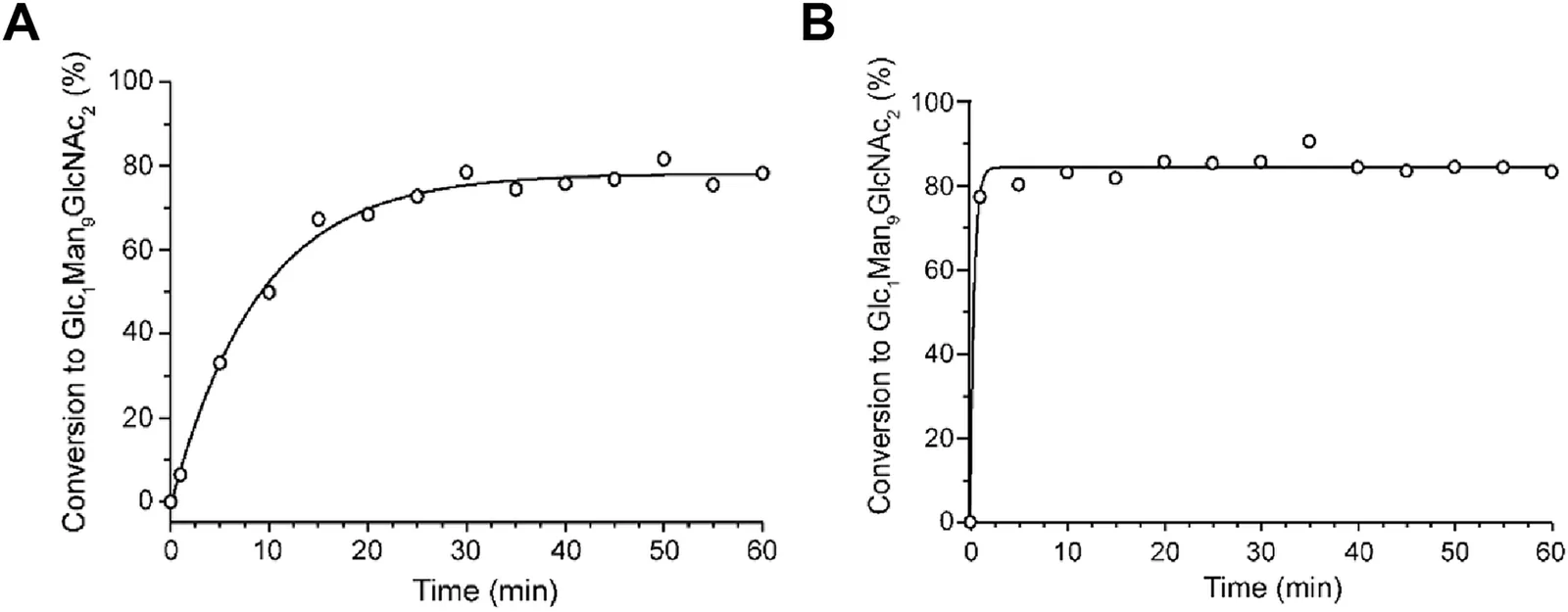
Comparison of the monoglucosylation capacity of human UGGT1 and *Chaetomium thermophilum* UGGT on MHC I/TAPBPR complexes. (**A**) Human UGGT1 (*Hs*UGGT1, 1 µM) (Sagert et al, 2023) or (**B**) *Chaetomium thermophilum* UGGT (*Ct*UGGT, 1 µM) was incubated with MHC I/TAPBPR complexes (3 μM) in 1× HBS buffer supplemented with UDP-glucose (UDP-Glc; 200 µM) and CaCl_2_ (5 mM) at room temperature. Conversion to the monoglucosylated Glc_1_Man_9_GlcNAc_2_ glycan (MHC I^glc.^) was analyzed by LC-MS. Source data are available online for this figure.

We first examined the glycan requirements of the MHC I transfer from TAPBPR to tapasin by our SEC-based approach (Fig. 1A). To this end, TAPBPR-chaperoned, peptide-receptive MHC I was incubated with *Chaetomium thermophilum* (*Ct*) UGGT in the presence of UDP-glucose and Ca^2+^ (Sagert et al, 2023). We used *Ct*UGGT throughout the study (hereafter UGGT), as it generates the same glycan pattern as human UGGT1 but exhibits superior stability and faster MHC I reglucosylation kinetics (Fig. EV3). UGGT efficiently monoglucosylated purified MHC I/TAPBPR heterodimers, yielding an MHC I species predominantly carrying the Glc_1_Man_9_GlcNAc_2_ monoglucosylated N-glycan (termed MHC I^glc.^ hereafter), as confirmed by LC-MS (Fig. 1B; Appendix Table S1). UGGT also modified MHC I glycans lacking one or two mannose residues on the B- and/or C-branches (Glc_1_Man_7-8_GlcNAc_2_), although with lower efficiency (Fig. 1B; Appendix Table S1). The lower reglucosylation efficiency of Man_8_GlcNAc_2_ compared to Man_9_GlcNAc_2_ glycan is consistent with the previously described activity of human UGGT1 (Parodi et al, 1983; Sousa and Parodi, 1995; Sousa et al, 1992).

When we compared the handover of TAPBPR-chaperoned monoglucosylated and mannose-only MHC I to tapasin in the presence of Crt using SEC, we observed the formation of a kinetically stable complex between MHC I, Crt, and tapasin-ERp57 only when the MHC I carried the monoglucosylated Glc_1_Man_8-9_GlcNAc_2_ glycan (Fig. 1C–E). In contrast, MHC I with a mannose-only Man_7-9_GlcNAc_2_ glycan failed to be recruited to the tapasin-ERp57 complex (Fig. 1C,D). Thus, monoglucosylation is required for Crt-dependent capture of MHC I by tapasin-ERp57.

Consistent with the logic of MHC I quality control, the presence of the HLA-A*68:02-restricted, high-affinity peptide ETVSKQSNV blocked transfer of MHC I to the tapasin-ERp57 chaperone complex (Fig. EV2), indicating that peptide loading prevents further chaperone engagement. Taken together, these findings indicate that TAPBPR-assisted reglucosylation of MHC I by UGGT1 governs the client exchange process.

### MHC I chaperone exchange is dependent on calreticulin

**Figure 2:**
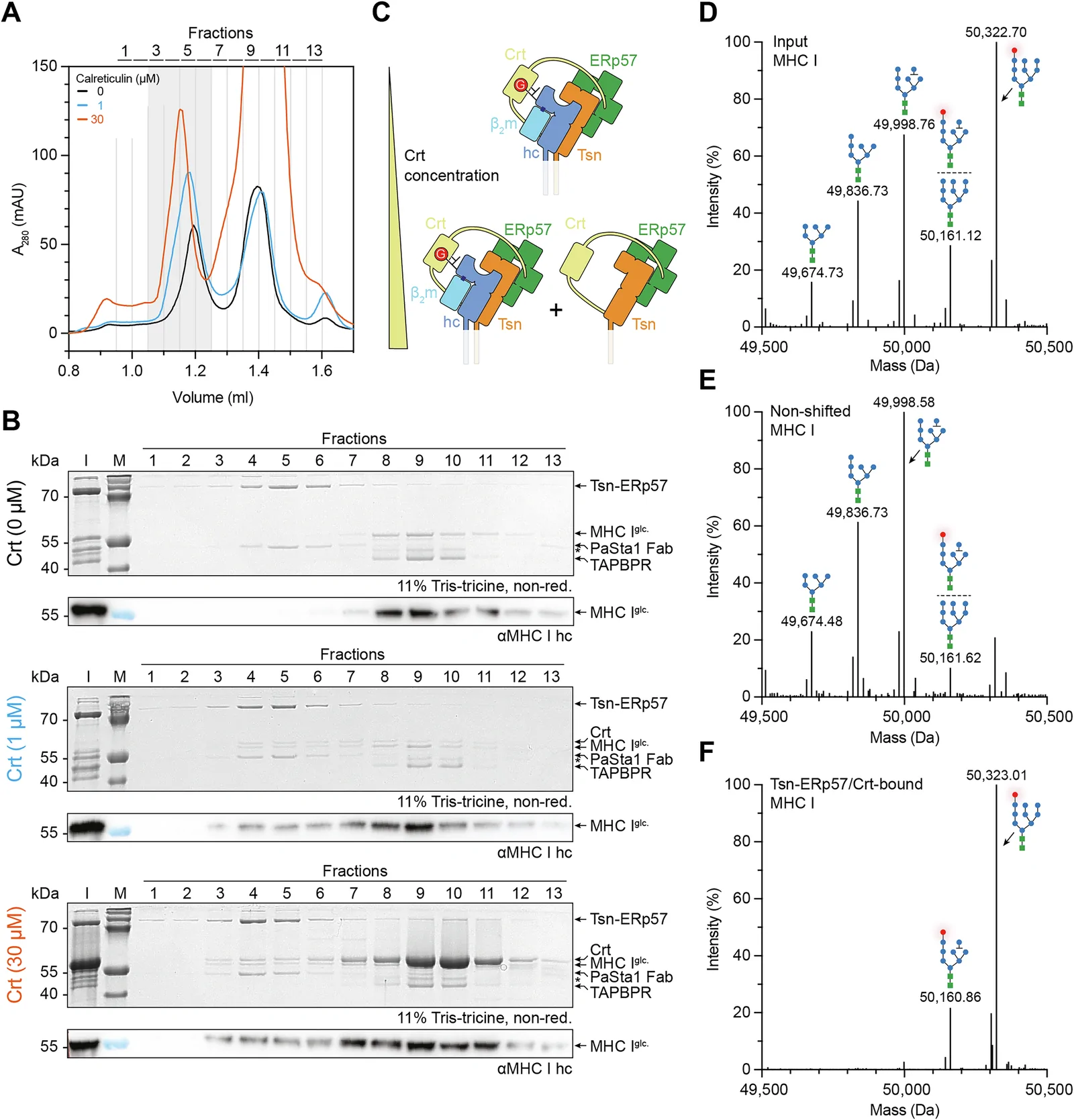
Calreticulin facilitates the transfer of monoglucosylated MHC I from TAPBPR to the tapasin-ERp57 quality control complex. (**A**) Size-exclusion chromatography (SEC) profiles of monoglucosylated β_2_m-HLA-A*68:02/TAPBPR (3 µM) incubated with tapasin (Tsn)-ERp57/PaSta1 (3 µM) in the presence of varying concentrations of calreticulin (Crt): no Crt (0 µM), substoichiometric Crt (1 µM), and tenfold excess Crt (30 µM). The gray shaded region indicates elution range of the Crt/MHC I^glc.^/tapasin-ERp57/PaSta1 complex. (**B**) SDS-PAGE analysis of SEC fractions from panel (**A**), including immunoblot analysis of the MHC I heavy chain (αMHC I hc, HC10 antibody). Depicted fractions correspond to an SEC elution volume range of 0.95–1.60 mL. (**C**) Schematic depiction of Crt concentration-dependent recruitment of monoglucosylated MHC I to the tapasin-ERp57 complex by Crt. (**D**–**F**) Deconvoluted mass spectra of single-chain β_2_m-HLA-A*68:02 before SEC (**D**, input MHC I), of TAPBPR-associated β_2_m-HLA-A*68:02 (**E**, non-shifted MHC I), or of tapasin-ERp57/Crt-bound MHC I. (**F**) Peak labels indicate the N-glycan structures attached to single-chain β_2_m-HLA-A*68:02 and the corresponding observed masses (Da) (green dot – GlcNAc; blue dot – Man; red dot – Glc). Data shown in panel (**E**) correspond to fraction 8 (1 μM Crt) in panel (**B**), whereas data shown in panel (**F**) correspond to fraction 4 (1 μM Crt) in panel (**B**). M marker, I input, kDa kilodalton, A_280_ absorption at 280 nm; αMHC I anti-MHC I heavy chain; * – impurity of β_2_m-HLA-A*68:02/TAPBPR complexes. Data shown in panels (**A**, **B**) are representative of three independent experiments (*n* = 3). Source data are available online for this figure.

To determine whether Crt is required for shuttling TAPBPR-chaperoned MHC I^glc.^ to tapasin, we monitored the formation of complexes between monoglucosylated MHC I and the tapasin-ERp57/PaSta1 heterotrimer at varying Crt concentrations (Fig. 2). When equimolar amounts of monoglucosylated MHC I/TAPBPR and tapasin-ERp57/PaSta1 (3 µM) were mixed in the absence of Crt, SEC revealed two separate peaks corresponding to the individual complexes (Fig. 2A,B). Addition of substoichiometric amounts of Crt (1 µM) led to co-migration of Crt with MHC I^glc.^/tapasin-ERp57/PaSta1, producing a single peak at a higher apparent molecular weight (Fig. 2A,B). This behavior is consistent with the formation of a pentameric complex comprising MHC I^glc.^, Crt, and the disulfide-linked tapasin-ERp57 heterodimer bound to PaSta1 Fab. Increasing the Crt concentration to a tenfold molar excess (30 µM) did not substantially increase formation of the pentameric complex, indicating that recruitment is primarily governed by the availability of monoglucosylated MHC I (Fig. 2B). We reasoned that the higher Crt concentration may also lead to the formation of a tetrameric Crt/tapasin-ERp57/PaSta1 complex (Fig. 2B), in which Crt engages in low-affinity interactions with the tapasin-ERp57 heterodimer. These results demonstrate that Crt is essential for bridging MHC I^glc.^ to tapasin-ERp57. We conclude that either the binding equilibria among MHC I quality control components are shifted towards the pentameric complex in the presence of Crt, or that Crt actively facilitates the extraction of peptide-receptive, monoglucosylated MHC I from TAPBPR.

### Calreticulin facilitates the transfer of monoglucosylated MHC I to tapasin

The globular lectin domain of Crt specifically recognizes monoglucosylated glycans on its client proteins via a concave β-sheet, forming a channel that engages the terminal four saccharides (Glc_1_Man_3_) of the glycan’s A-branch through an extensive network of hydrogen bonds and hydrophobic interactions (Kozlov et al, 2010). The tetrasaccharide represents the minimal binding unit and exhibits low-micromolar affinity (*K*_d_ ≈ 1 µM) for Crt, which specifically recognizes the presence of the glucose moiety (Kapoor et al, 2003; Kozlov et al, 2010).

To assess the functional consequences of this specificity, we used LC-MS to compare the N-glycan composition of MHC I molecules that were successfully transferred to the tapasin-ERp57/PaSta1 complex with those that remained bound to TAPBPR (Fig. 2D–F; Appendix Table S1). Strikingly, only monoglucosylated MHC I was transferred to the tapasin-ERp57/PaSta1 complex via Crt (Fig. 2F; Appendix Table S1), while MHC I carrying mannose-only glycans remained associated with TAPBPR during SEC (Fig. 2E; Appendix Table S1). These results demonstrate that Crt selectively targets monoglucosylated, peptide-receptive MHC I^glc.^ for handover to the tapasin-ERp57 editing complex, providing a crucial mechanistic explanation for how glycan processing ensures the fidelity and directionality of MHC I quality control.

### Calreticulin is recruited to TAPBPR-chaperoned, monoglucosylated MHC I

Peptide-receptive MHC I molecules are recruited to the PLC in association with Crt, where they receive and edit their peptide cargo (Trowitzsch and Tampé, 2020; Wearsch et al, 2011). Empty, partially misfolded, or suboptimally loaded MHC I molecules that escaped initial ER quality control by the PLC are thought to be captured by TAPBPR and reglucosylated by UGGT1 (Neerincx et al, 2017; Sagert et al, 2023; Wearsch et al, 2011; Zhang et al, 2011). These reglucosylated MHC I molecules were suggested to be re-engaged by Crt and directed back to the PLC for another round of peptide loading and editing (Neerincx et al, 2017; Wearsch et al, 2011; Zhang et al, 2011).

**Figure 3:**
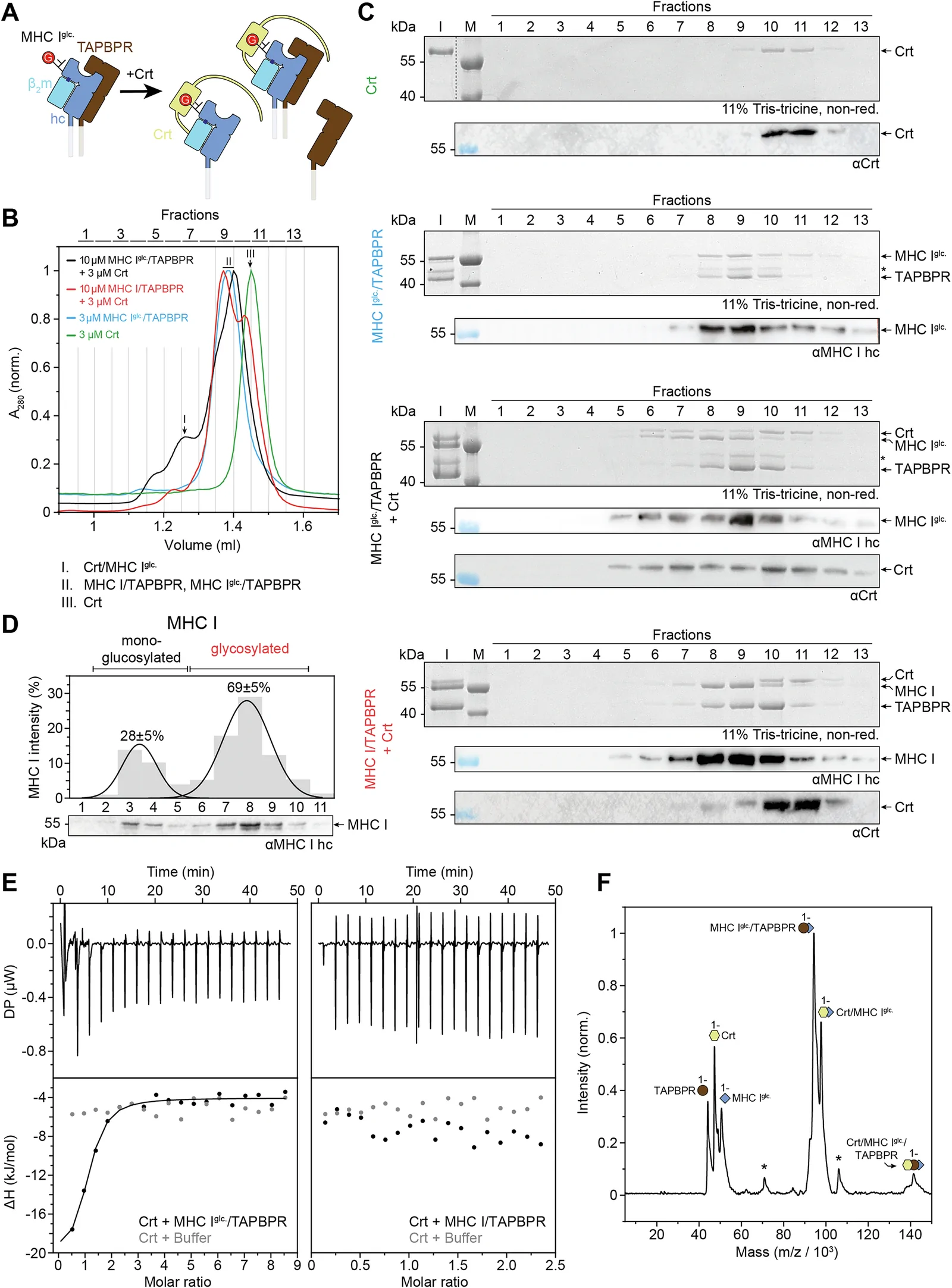
Monoglucosylation of MHC I attracts calreticulin to the TAPBPR-chaperoned complex for chaperone exchange. (**A**) Schematic depiction of the formation of MHC I intermediates upon calreticulin (Crt)-dependent engagement from the TAPBPR-chaperoned state. hc – MHC I heavy chain. (**B**) Size-exclusion chromatography (SEC) profiles of Crt alone (3 µM, green line), monoglucosylated β_2_m-HLA-A*68:02/TAPBPR (3 µM, blue line), a threefold molar excess of monoglucosylated β_2_m-HLA-A*68:02/TAPBPR (10 µM) over Crt (3 µM) (black line), and a threefold molar excess of untreated β_2_m-HLA-A*68:02/TAPBPR (10 µM) over Crt (3 µM, red line). The gray shaded region indicates the elution range of the Crt/MHC I^glc.^ intermediate. Roman numbers above each peak correspond to the proteins or protein complexes defined below. (**C**) Non-reducing SDS-PAGE and immunoblot analysis of SEC fractions from panel (**B**). The HC10 antibody (αMHC I hc) was used to detect the HLA-A*68:02 heavy chain, whereas the FMC75 antibody (αCrt) was used to detect Crt. Depicted fractions correspond to an SEC elution volume range of 0.95–1.60 mL. (**D**) Quantification of monoglucosylated MHC I species present in the isothermal titration calorimetry (ITC) sample containing Crt and monoglucosylated MHC I/TAPBPR. (**E**) Representative ITC experiment showing the interaction of Crt (titrant, 250 µM) with monoglucosylated MHC I/TAPBPR (analyte, 20 µM) (left). The corresponding non-monoglucosylated (mannose-only) MHC I/TAPBPR complex served as control (right). Integrated heats of Crt titrated into buffer are indicated in gray. (**F**) Ultrasoft laser-induced liquid bead ion desorption (LILBID) mass spectrometry analysis of complexes formed upon mixing Crt (3 µM) with the MHC I/TAPBPR complex (3 µM). Color coding is shown as in panel (**A**). Charge states of the individual proteins or complexes and the corresponding observed masses are indicated. Asterisks indicate unidentified complexes. M marker, I input, kDa kilodalton, A_280_ absorption at 280 nm; * – impurity corresponding to β_2_m-HLA-A*68:02/TAPBPR. Data shown in panels (**B**, **C**) are representative of three independent experiments (*n* = 3), whereas data shown in panel (**F**) are representative of two independent experiments (*n* = 2). Source data are available online for this figure.

We hypothesized that MHC I/Crt intermediates should be detectable in our in vitro handover assay. To test whether Crt can, in a Glc_1_Man_8-9_GlcNAc_2_ glycan-dependent manner, extract MHC I from MHC I/TAPBPR complexes, we first mixed Crt with monoglucosylated, TAPBPR-chaperoned MHC I in stoichiometric ratios (3 µM each) and analyzed changes in complex composition by SEC (Fig. 3A). Compared to Crt alone, Crt when mixed with monoglucosylated MHC I migrated at a larger apparent molecular weight, consistent with formation of an Crt/MHC I^glc.^ intermediate (Fig. 3B,C).

**Figure EV4:**
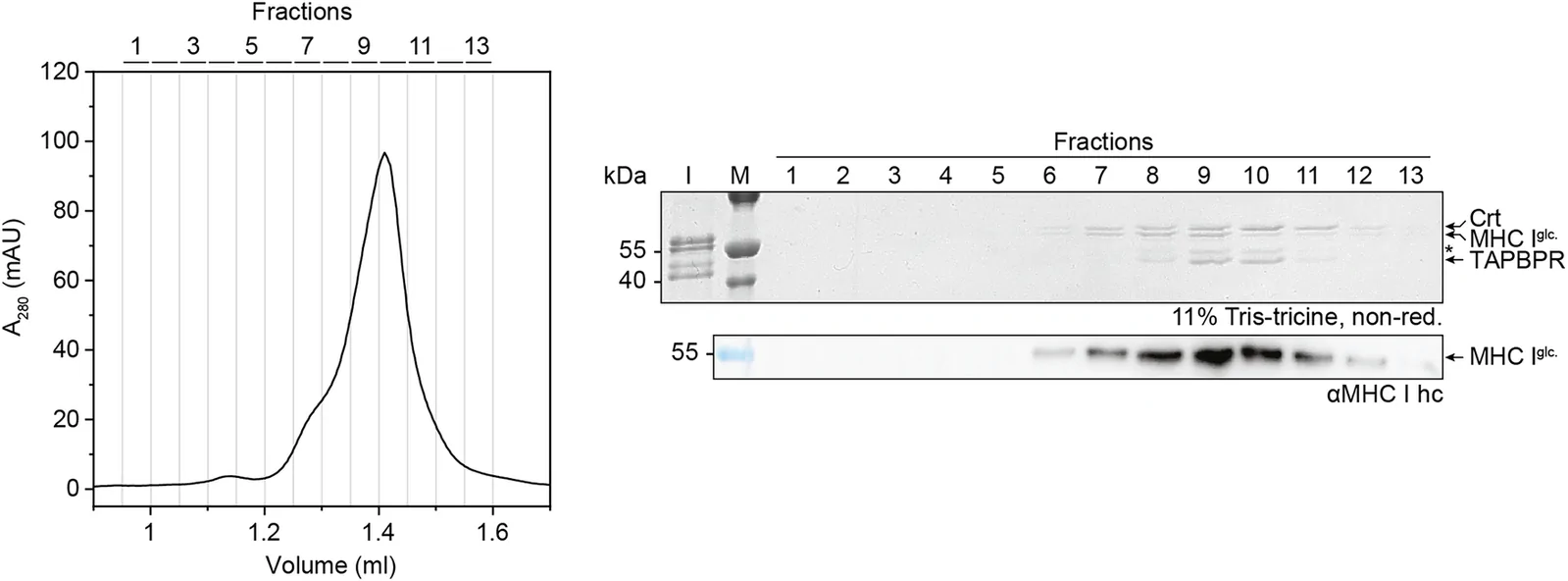
Chaperone exchange of MHC I at equimolar concentrations of MHC I^glc.^/TAPBPR and calreticulin. Size-exclusion chromatography (SEC) profiles (left) with SDS-PAGE and immunoblot analyses (right) of monoglucosylated single-chain β_2_m-HLA-A*68:02/TAPBPR complex and calreticulin (Crt) (3 µM each). The HC10 antibody was used to detect the MHC I heavy chain (αMHC I hc). M marker, I input, kDa kilodalton. Data shown in panels are representative of three independent experiments (*n* = 3). Source data are available online for this figure.

Based on the LC-MS glycan analyses (Figs. 1B and 2D–F; Appendix Table S1), we hypothesized that the limited availability of monoglucosylated MHC I constrains Crt/MHC I complex formation. Indeed, increasing the concentration of monoglucosylated, TAPBPR-chaperoned MHC I to 10 µM markedly enhanced complex formation, as confirmed by SDS-PAGE and immunoblot analysis (Figs. 3B,C and EV4). In contrast, non-monoglucosylated MHC I failed to shift Crt toward higher molecular weight species (Fig. 3C), indicating that Crt engagement strictly depends on glycan monoglucosylation.

We next set out to determine the equilibrium dissociation constant of Crt binding to TAPBPR-chaperoned monoglucosylated MHC I via ITC. To precisely quantify the amount of monoglucosylated MHC I that we used as analyte, we mixed UGGT-treated TAPBPR/MHC I with Crt and Tsn-ERp57, separated the formed complexes via SEC, and determined the ratio of Tsn-ERp57/Crt-bound MHC I (monoglucosylated) versus non-shifted MHC I (non-glucosylated) by immunoblotting followed by densitometric analysis (Fig. 3D). This quantification revealed that 28 ± 5% of the MHC I pool was shifted to the Tsn-ERp57/Crt-bound state and was thus reglucosylated (Fig. 3D). Consistent with the reported thermodynamics of Crt binding to the isolated tetrasaccharide Glcα1–3Manα1–2Manα1–2ManαMe only (Kapoor et al, 2003), we determined an equilibrium dissociation constant of Crt to monoglucosylated MHC I in the low-micromolar range (*K*_d_ = 0.7 ± 0.3 µM; Fig. 3E). Fitting of the thermogram data revealed a reduced effective cell concentration of monoglucosylated MHC I from 20 µM to 5.5 µM ± 0.4 µM consistent with the fraction of monoglucosylated MHC I determined by densitometry (Fig. 3D) and with the glycan composition analysis of UGGT-treated MHC I/TAPBPR by LC-MS (Figs. 1B and 2D–F).

To directly determine stoichiometries within the Crt/MHC I intermediates, we employed ultrasoft laser-induced liquid bead ion desorption (LILBID) mass spectrometry, which preserves noncovalent protein assemblies. LILBID-MS unambiguously detected the Crt/MHC I^glc.^ intermediate, as well as MHC I^glc.^/TAPBPR and Crt/MHC I^glc.^/TAPBPR complexes (Fig. 3F), providing strong evidence for the coexistence of these QC states.

**Figure EV5:**
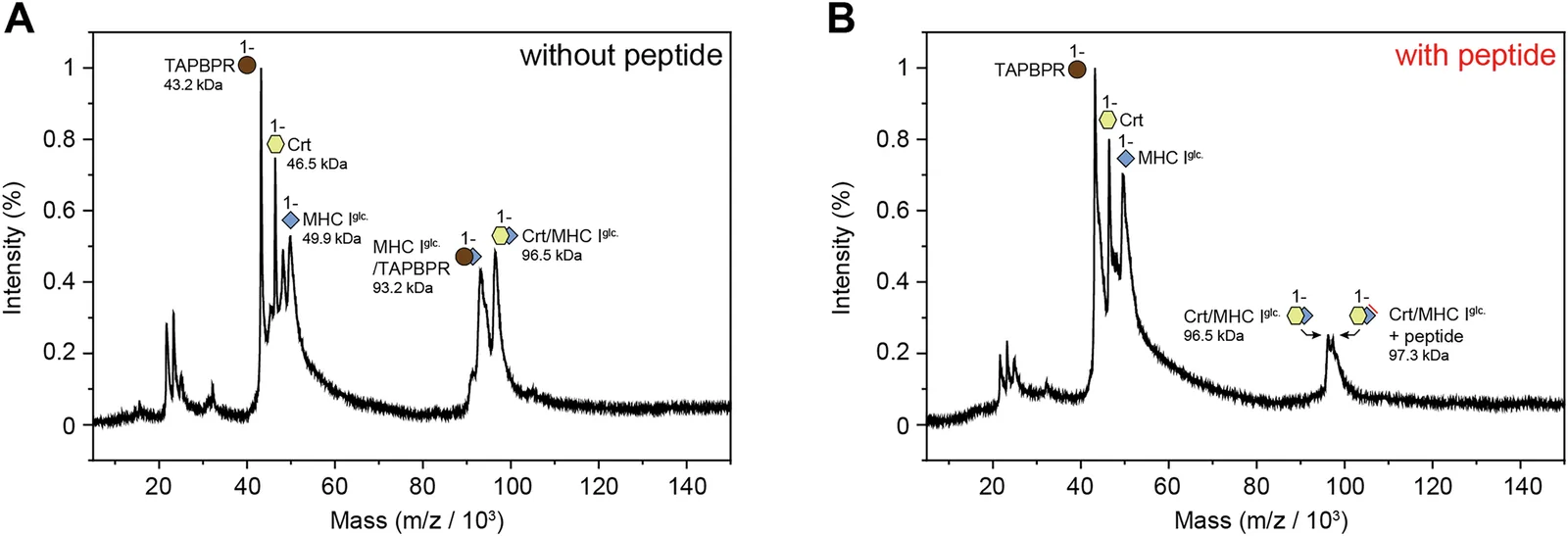
High-affinity peptide induces disengagement of MHC I from TAPBPR. (**A**,** B**) LILBID mass spectra of complexes formed upon mixture of monoglucosylated β_2_m-HLA-A*68:02/TAPBPR complex (10 µM) with calreticulin (Crt; 3 µM) in the absence (**A**) or presence (**B**) of peptide ETVSKQSNV (2.5 mM). Charge states of the individual proteins or complexes and the corresponding observed masses are indicated. Brown circle – TAPBPR; yellow hexagon – Crt; blue square – MHC I^glc.^. Source data are available online for this figure.

Notably, the Crt/MHC I^glc.^ complexes formed independently of the presence or absence of high-affinity peptide (Figs. EV2B and EV5), demonstrating that our system captures Crt/MHC I intermediates both before and after peptide acquisition during PLC-mediated editing. On the contrary, MHC I/TAPBPR complexes dissociated in the presence of high-affinity peptides (Figs. EV2A,B and EV5A,B).

In summary, these data show that monoglucosylation of MHC I is essential for Crt engagement, supporting a model in which Crt shifts the equilibrium of peptide-receptive MHC I^glc.^ from the TAPBPR-chaperoned state toward PLC recruitment.

### The acidic helix of calreticulin is dispensable for engaging monoglucosylated MHC I from TAPBPR

Within the PLC, Crt interacts with the membrane-proximal immunoglobulin C1 (IgC1) domain of tapasin through an extended acidic helix (Blees et al, 2017; Domnick et al, 2022) that precedes the C-terminal KDEL ER-retention sequence. In a subset of myeloproliferative neoplasms, frameshift mutants of Crt have been identified that comprise an altered amino acid composition of the C-terminal domain and lack the ER-retention signal (Klampfl et al, 2013; Nangalia et al, 2013). These mutants of Crt reverse the overall charge of the C-terminal helix, fail to incorporate into the PLC, and are predominantly secreted, likely due to the absence of the ER-retention signal (Arshad and Cresswell, 2018).

**Figure 4:**
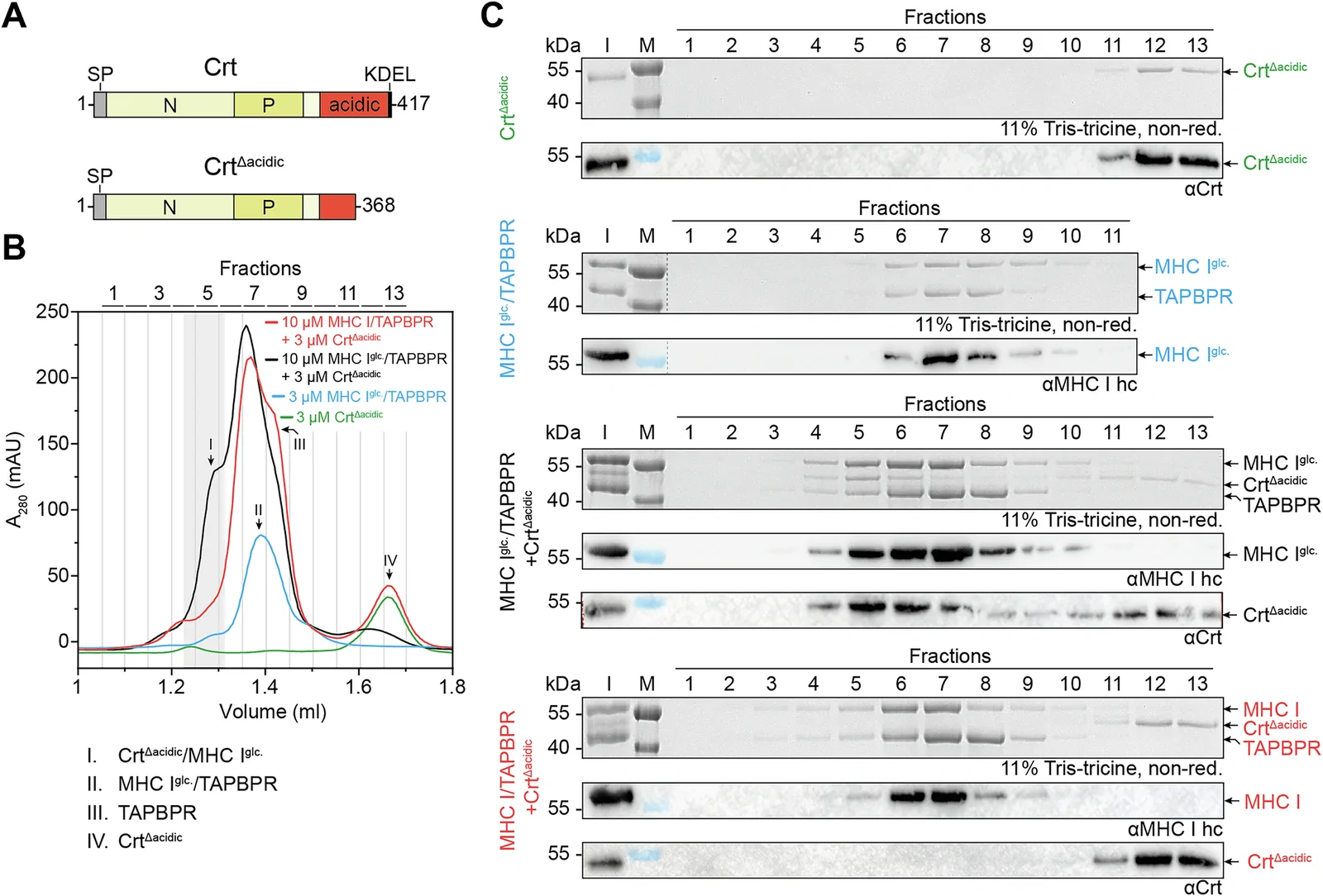
Calreticulin lacking its acidic helix binds monoglucosylated MHC I derived from a TAPBPR-chaperoned state. (**A**) Schematic representation of wild-type calreticulin (Crt) and Crt with a truncated acidic helix (Crt^Δacidic^). (**B**) Size-exclusion chromatography (SEC) profiles of Crt^Δacidic^ alone (3 µM, green line), monoglucosylated β_2_m-HLA-A*68:02/TAPBPR complex (3 µM, blue line), a threefold molar excess of monoglucosylated β_2_m-HLA-A*68:02/TAPBPR (10 µM) over Crt^Δacidic^ (3 µM, black line), and a threefold molar excess of untreated β_2_m-HLA-A*68:02/TAPBPR (10 µM) over Crt^Δacidic^ (3 µM, red line). The gray shaded region indicates the elution range of the Crt^Δacidic^/MHC I^glc.^ intermediate. Roman numbers above each peak correspond to the proteins or protein complexes defined below. (**C**) Non-reducing SDS-PAGE and immunoblot analysis of SEC fractions from panel (**B**), detecting the MHC I heavy chain (αMHC I hc, HC10 antibody) and Crt (αCrt, FMC75 antibody). Depicted fractions correspond to an SEC elution volume range of 1.05–1.70 mL. KDEL—ER-retention signal lysine-aspartate-glutamate-leucine, SP signal peptide, N N-domain, P P-domain, A_280_ absorption at 280 nm, kDa kilodalton, I input sample, M protein size marker. Data shown in panels (**B**, **C**) are representative of three independent experiments (*n* = 3). Source data are available online for this figure.

To explore the role of the C-terminal acidic helix in MHC I-chaperone exchange, we recombinantly produced a Crt variant lacking part of the acidic helix (Crt^Δacidic^; Fig. 4A). We then tested whether Crt^Δacidic^ could engage monoglucosylated MHC I in the TAPBPR-chaperoned complex by comparing its effect on the monoglucosylated MHC I/TAPBPR complex to the mannose-only MHC I/TAPBPR complex (Fig. 4B). Similar to wild-type Crt, a pronounced shift to higher apparent molecular weight was evident in SEC (Fig. 3B,C; fractions 6 and 7), indicating co-migration of Crt^Δacidic^ and monoglucosylated MHC I. This interaction was further confirmed by SDS-PAGE and immunoblotting (Fig. 4C). As observed for wild-type Crt, Crt^Δacidic^ did not co-migrate with mannose-only (non-reglucosylated) MHC I (Fig. 4B,C).

These results demonstrate that the C-terminal acidic helix of Crt is dispensable for engaging monoglucosylated MHC I in the TAPBPR-chaperoned state, while recognition of the monoglucosylated glycan of MHC I by the lectin domain of Crt is essential for this process (Sadasivan et al, 1996).

### The acidic helix of calreticulin is essential for chaperone exchange

We next examined whether the Crt^Δacidic^ variant facilitated transfer of MHC I^glc.^ from the TAPBPR- to the tapasin-chaperoned state. Crt engages in several low-affinity interactions with the PLC editing module (Del Cid et al, 2010). Aside from the lectin-Glc_1_Man_3_ interaction, Crt binds ERp57 via the tip of its P domain and cradles tapasin with its C-terminal acidic helix (Blees et al, 2017; Domnick et al, 2022). Together, these interactions enable Crt to act as a “safety belt” for peptide-receptive MHC I within the PLC and ensure MHC I’s close association with tapasin (Blees et al, 2017; Domnick et al, 2022). We therefore hypothesized that Crt variants lacking one or more of these interactions would be unable to transfer monoglucosylated MHC I from TAPBPR to tapasin.

**Figure 5:**
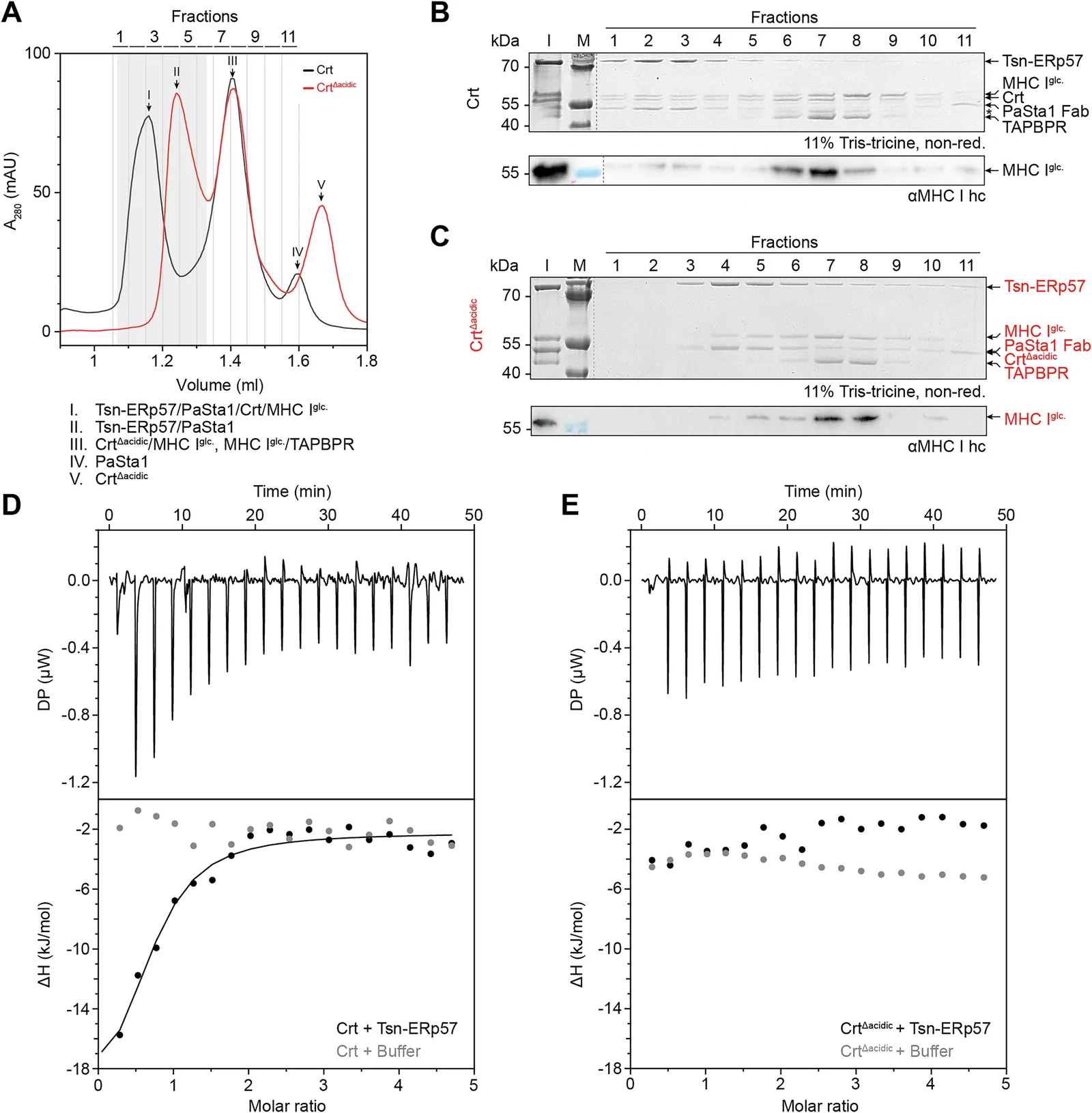
The C-terminal acidic helix of calreticulin is essential for the exchange of monoglucosylated MHC I from TAPBPR to tapasin. (**A**) Size-exclusion chromatography (SEC) profiles of calreticulin (Crt, black line) or Crt lacking the acidic helix (Crt^Δacidic^, red line) (3 µM each) incubated with the tapasin-ERp57/PaSta1 (3 µM) and monoglucosylated, peptide-receptive HLA-A*68:02/TAPBPR (3 µM). The gray shaded region indicates the differences in elution range between the Crt/MHC I^glc.^/tapasin-ERp57/PaSta1 and the tapasin-ERp57/PaSta1 complex. Roman numbers above each peak correspond to the proteins or protein complexes defined below. (**B**, **C**) Non-reducing SDS-PAGE and immunoblot analysis (αMHC I hc, HC10 antibody) of SEC fractions shown in panel (**A**), assessing the ability of Crt (**B**) and Crt^Δacidic^ (**C**) to promote transfer of MHC I to tapasin-ERp57. Depicted fractions correspond to an SEC elution volume range of 1.05–1.60 mL. (**D**,** E**) Representative isothermal titration calorimetry (ITC) experiments showing binding of Crt (**D**) or Crt^Δacidic^ (**E**) (black; titrant, 500 µM) to the tapasin-ERp57 heterodimer (analyte, 19 µM). Control experiments of Crt or Crt^Δacidic^ into buffer (50 mM HEPES-NaOH, pH 7.4, 150 mM NaCl, 1 mM CaCl_2_) are shown in gray. A_280_ absorption at 280 nm; kDa kilodalton, I input sample, M protein size marker. Data shown in panels (**A**–**C**) are representative of three independent experiments (*n* = 3). Source data are available online for this figure.

To test this, we mixed monoglucosylated MHC I/TAPBPR complexes and tapasin-ERp57/PaSta1 with either wild-type Crt or the Crt^Δacidic^ variant and analyzed complex formation by SEC and SDS-PAGE (Fig. 5). The presence of Crt, in combination with the monoglucosylated MHC I/TAPBPR complex and tapasin-ERp57/PaSta1, resulted in a shift of monoglucosylated MHC I to the tapasin-ERp57/PaSta1 complex migrating at higher apparent molecular weight (Fig. 5A,B). On the other hand, Crt^Δacidic^ was unable to transfer MHC I^glc.^ to tapasin-ERp57/PaSta1 as revealed by SEC and SDS-PAGE analysis (Fig. 5A,C).

To assess whether deletion of the acidic helix of Crt affects the thermodynamics of its interaction with the tapasin-ERp57 complex, we performed ITC by titrating either Crt or Crt^Δacidic^ (500 µM each) to tapasin-ERp57 (19 µM). Equilibrium dissociation constants (*K*_*d*_) were derived from these measurements (Fig. 5D,E). Crt bound the soluble tapasin-ERp57 complex with a *K*_d_ of 4.2 ± 2.3 µM (Fig. 5D), whereas no binding of Crt^Δacidic^ was detected under the same conditions (Fig. 5E).

These data demonstrate that the C-terminal acidic helix of Crt is essential for docking monoglucosylated MHC I onto the tapasin-ERp57 heterodimer during chaperone exchange. Notably, our in vitro findings align with cellular experiments showing that Crt frameshift mutants characterized by transformation of the acidic helix to a basic helix and the loss of the ER-retention motif, do not incorporate into the PLC and fail to rescue peptide-receptive MHC I (Arshad and Cresswell, 2018; Elf et al, 2016).

## Discussion

Here, we reconstituted the sequential transfer of MHC I between downstream and upstream ER quality control (QC) factors using purified components. This reductionist system enabled us to dissect the QC “handover network” that governs how peptide-receptive MHC I complexes are captured, retained, reglucosylated, and eventually delivered back to the PLC for high-affinity peptide editing and loading. Central to this process is the UGGT1-dependent monoglucosylation of MHC I, which drives engagement with the Cnx/Crt cycle and prevents premature ER exit (Gao et al, 2002; Ninagawa et al, 2024; Sagert et al, 2023). TAPBPR acts down- and upstream of this step by stabilizing peptide-receptive MHC I and maintaining them in a QC-competent state, thereby facilitating reglucosylation by UGGT1 (Boyle et al, 2013; Neerincx et al, 2017; Sagert et al, 2023).

**Figure 6:**
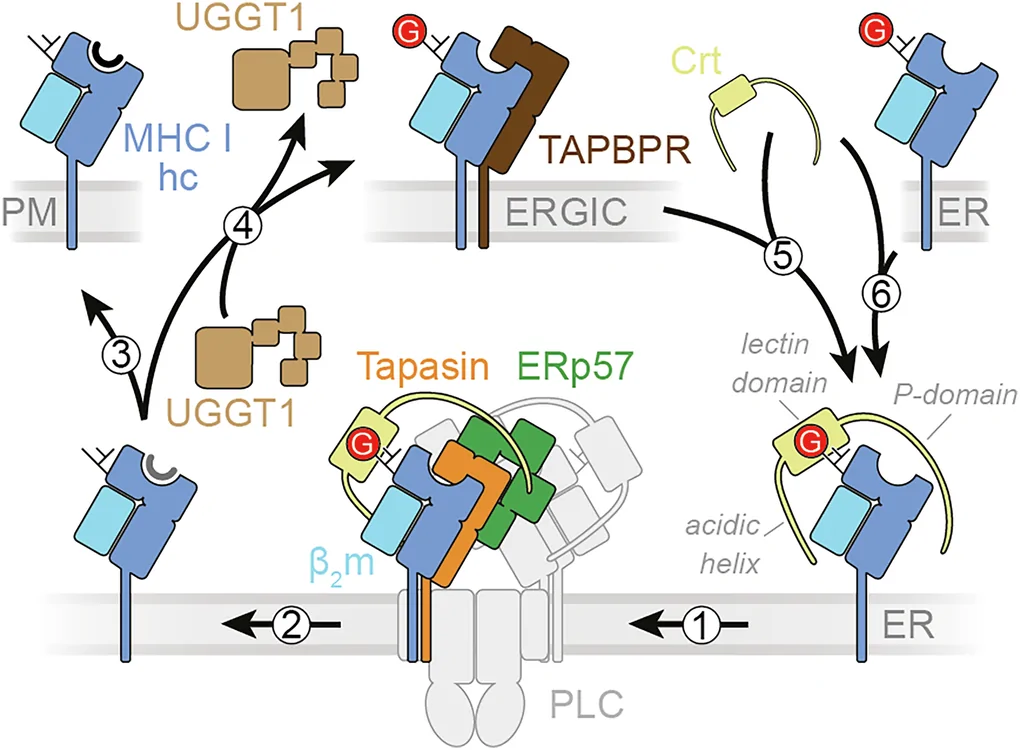
Model of MHC I recycling by a dedicated chaperone network. Monoglucosylated, peptide-receptive MHC I (MHC I hc, blue; β_2_m, light blue) is recognized and escorted by the lectin-like ER chaperone calreticulin (Crt, yellow) to the peptide-loading complex (PLC) for peptide loading and editing (1). MHC I molecules loaded with optimal (high-affinity) peptide cargo are released from the PLC and fully deglucosylated by α-glucosidase II, which removes the terminal glucose (2). MHC I molecules carrying high-affinity peptides and a Man_9_GlcNAc_2_ glycan are licensed to traffic through the secretory pathway to present their antigenic cargo at the plasma membrane (PM) (3). Peptide/MHC I complexes that escaped the PLC with suboptimal (low-affinity) peptides are recognized in a secondary quality control step by TAPBPR and are reglucosylated by the glycoprotein folding sensor UDP-glucose:glycoprotein glucosyltransferase (UGGT1) (4). Reglucosylated, peptide-receptive MHC I molecules are re-engaged by Crt and trafficked back to the ER (5), where they can re-enter the PLC for another round of peptide loading and editing. Additionally, the N-glycan of newly synthesized and translocated MHC I is trimmed by glucosidases I and II to a monoglucosylated glycan, which is recognized by Crt for docking to the PLC (6). The extent to which different MHC I allomorphs depend on tapasin or TAPBPR for editing of their peptide repertoire is also influenced by their intrinsic binding affinities for MHC I-specific chaperones.

Our data support a model in which antigen presentation fidelity arises from a series of reversible QC checkpoints spanning the PLC with tapasin-ERp57, TAPBPR, UGGT1, and Crt. These checkpoints jointly safeguard against premature ER exit of incompletely edited MHC I. Consistent with reports that PLC-associated MHC I carries exclusively monoglucosylated N-glycans (Domnick et al, 2022; Wearsch et al, 2004; Wearsch et al, 2011), we show that monoglucosylation is not merely permissive but essential for productive Crt-mediated retrieval from TAPBPR—a process that has been challenging to resolve in living cells (Wearsch et al, 2011; Zhang et al, 2011). While our data show that glycan recognition by Crt is essential to extract MHC I from TAPBPR, successful transfer to the tapasin-ERp57 editing complex additionally requires the functional engagement of Crt’s lectin domain and acidic C-terminal helix with tapasin. The observed increase in affinity of full-length Crt for the tapasin-ERp57 complex compared to the isolated P-domain/ERp57 interaction suggests that the C-terminal acidic helix provides an additional binding interface, thereby enhancing recruitment to the PLC. These results highlight the importance of multivalent, low-affinity interactions in governing the specificity and directionality of MHC I chaperone exchange. Crt thus acts as a molecular shuttle between TAPBPR-bound MHC I and components of the PLC, driven by affinity-based redistribution within a dynamic network of competing interactions. In this context, monoglucosylation functions as a molecular switch that selectively stabilizes Crt-MHC I interactions in the absence of TAPBPR (Fig. 6).

A clear conceptual distinction emerges between UGGT1- and Crt-mediated recognition of MHC I. Calreticulin primarily recognizes the monoglucosylated glycan and can engage both peptide-loaded and peptide-receptive MHC I. By contrast, UGGT1 acts as a folding sensor that selectively reglucosylates conformationally dynamic or incompletely matured glycoproteins. In the case of MHC I, peptide-deficient or suboptimally loaded conformers likely expose structural features recognized by UGGT1, whereas calreticulin engagement occurs downstream once the monoglucosylated glycan has been installed. In this framework, TAPBPR may stabilize a peptide-receptive, yet conformationally flexible MHC I state that remains competent for UGGT1 recognition and reglucosylation, thereby promoting re-entry into the calreticulin-dependent quality-control cycle.

A notable outcome of our reductionist system is the capture of a stable Crt/MHC I^glc.^ intermediate, supporting the notion that Crt alone can act as a selective chaperone for peptide-receptive MHC I^glc.^ (Howe et al, 2009). This intermediate likely represents an obligate handover state bridging TAPBPR-mediated retention with tapasin-mediated peptide editing. The existence of this intermediate clarifies how Crt can coordinate the retrograde trafficking of incompletely loaded MHC I, complementing but not functionally replacing tapasin and ERp57 within the PLC. Our findings refine the current model of MHC I quality control by introducing a multivalent, glycan-gated recruitment mechanism in which Crt integrates information from both the glycan status of the client and the availability of PLC components. Within this framework, UGGT1-mediated reglucosylation, with or without the assistance of TAPBPR, marks suboptimally loaded MHC I molecules for re-engagement. Crt then selectively captures these substrates, while multivalent interactions—including those mediated by its acidic helix—promote their stabilization within the PLC. This mechanism provides both specificity and reversibility, two key features required for maintaining high fidelity in antigen presentation.

Our system also provides insights into the functional defects associated with disease-linked Crt frameshift mutants, which comprise a basic C-terminal segment instead of the acidic helix and lack an ER-retention signal (Arshad and Cresswell, 2018; Elf et al, 2016; Klampfl et al, 2013). Although our Crt^Δacidic^ construct does not directly mimic disease-associated calreticulin frameshift mutants, the observed defect in tapasin-ERp57 recruitment is consistent with previous cellular studies reporting impaired PLC incorporation and defective MHC I maturation for these variants (Arshad and Cresswell, 2018). The inability of these mutants to support PLC function provides a mechanistic explanation for reduced surface MHC I levels observed in pathological settings. Future work using authentic disease-associated mutants will be required to disentangle the contributions of charge reversal, altered sequence composition, and loss of ER retention to these phenotypes.

Regarding MHC I allomorph-specific behavior, we focused on the TAPBPR-dependent HLA-A*68:02 allomorph, which provides a biochemically tractable scaffold for dissecting QC transitions (Bashirova et al, 2020; Ilca et al, 2019; Purcell et al, 2001; Rizvi et al, 2014). Notably, HLA-A*68:02 is readily transferred from TAPBPR to tapasin-ERp57, suggesting that both tapasin-dependent and tapasin-independent allomorphs can traverse the PLC when provided access to the appropriate QC machinery. However, future studies will be required to determine whether similar glycan-dependent recycling mechanisms also operate in MHC I allotypes with reduced or absent TAPBPR dependence. Our observations are nonetheless consistent with the conceptual division of MHC I allomorphs into ‘generalists’, which bind broad peptide repertoires but require more extensive quality control, and ‘specialists’, which rapidly stabilize upon binding a narrow set of high-affinity peptides (Chappell et al, 2015).

Direct evidence for TAPBPR-independent ‘specialist’ allotypes in the human system remains limited. It therefore is unclear how broadly the mechanism described here applies across the MHC I repertoire. Although allotype-specific differences in tapasin and TAPBPR dependence are well documented in humans, it is unresolved whether these differences map onto the ‘generalist/specialist’ dichotomy originally proposed for the chicken MHC system (Chappell et al, 2015). Together, our mechanistic model provides general principles explaining how distinct MHC I allomorphs achieve high-fidelity peptide selection while relying to varying degrees on TAPBPR, UGGT1, Crt, and tapasin (Ilca et al, 2019; Neerincx et al, 2017; Sun et al, 2023).

A key motivation for developing our reconstituted system was the inability of cellular assays to isolate transient intermediates or resolve the temporal order of MHC I interactions with TAPBPR, UGGT1, Crt, and tapasin-ERp57. Accordingly, our reconstituted system does not address the role of intracellular compartmentalization in shaping MHC I quality control. In cells, anterograde and retrograde trafficking between the endoplasmic reticulum and the *cis*-Golgi is mediated by COPII- and COPI-coated vesicles, respectively, imposing spatial and temporal constraints on chaperone–client interactions. This vesicular transport machinery adds an additional layer of regulation, as partially folded or suboptimally loaded MHC I molecules can cycle between compartments rather than remaining confined to a single environment. Such compartmental separation of folding, editing, and retrieval is likely to influence both the kinetics and directionality of MHC I maturation.

While our in vitro system captures the core biochemical interactions and thermodynamics underlying glycan-dependent recruitment to the PLC, it does not recapitulate its spatial organization. Nonetheless, the affinity-driven redistribution mechanism described here operates within a broader framework in which COPII-mediated export and COPI-dependent retrieval dynamically regulate the availability of both MHC I clients and chaperones. Such a cycle could enhance the efficiency of quality control by repeatedly exposing suboptimally loaded molecules to distinct editing environments.

Our reconstituted system enables the detection of short-lived intermediates that are essential not only for mechanistic analysis but also for future structural studies. While the major predictions of our model can be tested in cellulo—for example, through glycan-deficient MHC I mutants, Crt C-terminal variants or lectin-dead mutants (Kapoor et al, 2004), by modulating UGGT1 activity, or by using tapasin/TAPBPR affinity mutants—the absence of such cellular data does not diminish the value of establishing a biochemical blueprint for MHC I QC transitions. Rather, it provides an indispensable foundation for designing focused and interpretable cellular assays. In this context, it is of note that calreticulin is a highly abundant ER-resident chaperone that is present in concentrations ranging from tens to hundreds of micromolar, whereas TAPBPR is expressed at substantially lower levels. Considering the low-micromolar affinities measured here, these concentration differences establish conditions that strongly favor Crt-mediated engagement of reglucosylated MHC I and recruitment into the peptide-loading complex environment. Future studies will be required to determine to what extent compartmentalization further contributes to the selection and stabilization of high-affinity peptide–MHC I complexes.

We note, however, that our reductionist system inevitably simplifies the complex environment of the ER. It does not account for membrane context, molecular crowding, local Ca²⁺ dynamics, or post-ER trafficking pathways, and it focuses on a single TAPBPR-dependent allomorph. Consequently, while this approach provides mechanistic resolution that is difficult to achieve in cellulo, future studies integrating live-cell imaging and targeted genetic engineering will be crucial for quantifying how strongly each pathway contributes under physiological conditions.

## Methods

**Table:** Reagents and tools table

Reagent/resource	Reference or source	Identifier or catalog number
**Experimental models**		
EMBacY	Trowitzsch et al, 2010	
EXPI293F	Gibco	A14527
HEK293-F	Thermo Fisher	R79007
Rosetta DE3 pLysS	Sigma-Aldrich	70956-3
SF21	Thermo Fisher	11496015
**Recombinant DNA**		
pAMI-Tapasin^ΔTMD^-ERp57^C60A^	Müller et al, 2022	
pcDNA3.1-A68:02^ΔTMD^-β_2_m	O’Rourke et al, 2019	
pcDNA3.1-PaSta1 Fab lc	Prof. Dr. D. Margulies	
pcDNA3.1-PaSta1 Fab hc	Prof. Dr. D. Margulies	
pcDNA3.1-TAPBPR^ΔTMD^	O’Rourke et al, 2019	
pETM-Crt	This study	
pETM-Crt^Δacidic^	This study	
pHLsec-*Ct*UGGT	This study	
**Antibodies**		
α-Crt (FMC75)	Abcam	ab22683
α-MHC I hc (HC10)	In-house production (hybridoma)	
α-mouse HRP conjugate	Sigma-Aldrich	A-2554
**Oligonucleotides and other sequence-based reagents**		
ETVSKQSNV peptide	This study	
**Chemicals, Enzymes and other reagents**		
Agar	Thermo Fisher	12357153
Amicon Ultra (3, 10, 30, 50 kDa MWCO)	Merck Millipore	UFC505096
Ammonium acetate	Sigma-Aldrich	5330040050
Ammonium persulfate (APS)	Carl Roth	9592.1
Ampicillin sodium salt	Carl Roth	K029.2
Avidin	IBA Lifesciences	2-0204-050
Benzamidine hydrochloride monohydrate	Carl Roth	CN38.3
Benzonase Nuclease	Merck Millipore	70664-3
Blotting Paper	Macherey-Nagel	742125
Bluo-Gal	BioMol	50375.1
Bromophenolblue	Carl Roth	A512.1
Calcium chloride dihydrate	Carl Roth	5239.1
Clarity Western ECL substrate	Bio-Rad	1705061
Desthiobiotin	IBA Lifesciences	2-1000-002
Disodium hydrogenphosphate	Carl Roth	4984.3
Econo-Pac Disposable Chromatography columns	Bio-Rad	7321010
EGTA	Carl Roth	3054.1
Expi293™expression medium	Gibco	A1435101
Formic acid	Thermo Fisher	A11750
FreeStyle 293 Expression Medium	Gibco	12338026
Gentamycin sulfate	Carl Roth	0233.3
Glycerol	Sigma-Aldrich	G5516
Hydrochloric acid (HCl)	Sigma-Aldrich	320331
Hydroxyethyl piperazinyl ethanesulfonic acid (HEPES)	Carl Roth	9105.6
Imidazole	Sigma-Aldrich	56750
InstantBlue Coomassie	abcam	ab119211
Isopropyl alcohol	Carl Roth	6752.4
Isopropyl-β-D-1-thiogalactopyranoside (IPTG)	Carbolution	CC06004
Kanamycin sulfate	Carl Roth	T832.4
Kifunensine	BioMol	Cay10009437-50
LB broth base	Thermo Fisher	12780029
Linear PEI	Polysciences	23966-100
Milk powder	Carl Roth	T145.3
Ni^2+^-NTA agarose resin	Thermo Fisher	R90110
NucleoBond Xtra midi kit	Macherey-Nagel	740410.100
Page ruler prestained protein ladder	Thermo Fisher	26616
Parafilm M	Biozym	743311
PMSF	Carl Roth	6367.4
Potassium chloride	Carl Roth	6781.1
Potassium dihydrogenphosphate	Carl Roth	3904.1
PureYield Plasmid Midiprep System	Promega	A2492
ROTIPHORESE Gel 40 Acrylamide/bisacrylamide (1:19, v/v)	Carl Roth	3030.1
ROTI PVDF membrane, 0.45 µm	Carl Roth	T200.1
SF-900TM II SFM	Gibco	10902104
Slide-A-Lyzer Dialysis Casette, 10 kDa MW cut off	Thermo Fisher	66110
Sodium azide	Carl Roth	K305.1
Sodium chloride (NaCl)	Carl Roth	3957.1
Sodium hydroxide	Carl Roth	6771.1
Sodium dodecyl sulfate (SDS)	Carl Roth	CN30.3
Spectra/Por® 7 dialysis membrane, 3.5 k MW cut off	Carl Roth	E861.1
Strep-Tactin Sepharose resin	IBA Lifesciences	2-1201-010
Tetracycline hydrochloride	Carl Roth	HP63.1
Tetramethylethylenediamin (TEMED)	Carl Roth	2367.3
Trichloroacetonitrile	Thermo Fisher	139371000
Tricine	Carl Roth	6977.5
Tris	Carl Roth	4855.3
Tryptone	Carl Roth	6681.5
Tween 20	Carl Roth	9127.2
UDP-Glucose	Sigma-Aldrich	U4625
Valproic acid	Merck Millipore	P4543-25G
X-tremeGENE HP DNA-transfection reagent	Sigma-Aldrich	6366236001
Yeast extract	Carl Roth	2904.3
Zeba Spin Desalting column, 2 ml	Thermo Fisher	89890
**Software**		
Adobe Illustrator 2024	Adobe	
Excel	Microsoft	
MicroCal PEAQ-ITC Analysis Software Version 1.21	Malvern Panalytical	
OriginPro 2022b	Origin Lab	
ImageJ	Schneider et al, 2012	
Unify 3.11	Waters	
Word	Microsoft	
**Other**		
BioAccord	Waters	
Fusion FX	Vilber	
Incubator (Multitron)	HT infors	
Laminar flow hood	Heraeus, Holton	
MicroCal PEAQ-ITC	Malvern Panalytical	
Mini-PROTEAN electrophoresis system	Bio-Rad	
NanoDrop-1000	PEQLAB Biotechnology	
New Brunswick Innova 43	Eppendorf	
FPLC system (Äkta ETTAN/Äkta Go)	GE Healthcare/Cytiva	
Sonifier 250	Branson	
Superdex 200 Increase column (3.2/300, 10/300)	GE Healthcare	
ThermoMixer	Eppendorf	
Trans-Blot Turbo Transfer System	Bio-Rad	
Ultracentrifuge XE-90	Beckman Coulter	

### Plasmid constructs

Single-chain β_2_m-HLA-A*68:02 (HLA-A68), human TAPBPR, human tapasin^ΔTMD^-ERp57^C36A^, and *Ct*UGGT were previously described (Müller et al, 2022; O’Rourke et al, 2019; Roversi et al, 2017). PaSta1 Fab constructs (heavy and light chain) were kindly provided by David H. Margulies (Jiang et al, 2022). Human, full-length calreticulin (UniProtKB P27797) with an N-terminal, TEV-cleavable His_6_-tag lacking the signal peptide (amino acids 18-417) and the calreticulin variant lacking part of the C-terminal acidic helix (amino acids 18-369) were cloned into the pETM-11 vector (Gunter Stier, European Molecular Biology Laboratory).

### Cell culture and transfection of HEK293-F/Expi293 cells

HEK293-F or Expi293 cells were grown in FreeStyle 293 Expression Medium (Gibco) at 37 °C, 5% CO_2_, and shaken at 120 rpm. For co-expression of the MHC I^Fos^ and TAPBPR^Jun^, 5 mL expression medium was supplemented with 75 μg of β_2_m-HLA-A68^Fos^ and 75 μg of TAPBPR^Jun^ DNA (equimolar ratio) and mixed with a four-fold molar excess of linear PEI (Polysciences) diluted in 5 mL expression medium. Similarly, co-expression of PaSta1 Fab was performed with a 1:2 molar ratio of PaSta1 Fab DNA of heavy chain (50 µg) and light chain (100 µg). In accordance, expression of *Chaetomium thermophilum* (*Ct*) UGGT was performed with 150 µg of *Ct*UGGT plasmid DNA. All plasmid DNA used for transfection was purified using the PureYield Plasmid Midiprep System (Promega). After incubation for 30 min, the transfection mixture was added to 50 mL of HEK293-F cells at a density of 16 × 10^6^ cells/mL. Three hours post transfection, cells were diluted to 3 × 10^6^ cells/mL and 3.5 mM valproic acid (Merck Millipore), and 10 μM kifunensine (Biomol) were added. The culture supernatant containing the secreted proteins was harvested five days after transfection.

### Purification of *Ct*UGGT

To purify secreted *Ct*UGGT from transfected HEK293 cells, the culture supernatant was cleared from cells by centrifugation at 2000×g for 10 min and dialyzed twice against 1xPBS (1.5 mM KH_2_PO_4_, 8 mM Na_2_HPO_4_, 137 mM NaCl, 2.7 mM KCl, pH 7.6) for 3 h and overnight, respectively. Ni^2+^-NTA beads (Thermo Fisher Scientific) were added to the supernatant and stirred overnight at 4 °C. Metal-affinity chromatography was performed in PBS, supplemented with 20 mM imidazole and 5% (v/v) glycerol as wash buffer. *Ct*UGGT was eluted with 1xPBS supplemented with 200 mM imidazole and 5% (v/v) glycerol. Monodisperse UGGT was isolated by size-exclusion chromatography (SEC, Superdex 200 Increase 10/300, Cytiva) in 1xHBS buffer (50 mM HEPES-NaOH, pH 7.4, 150 mM NaCl). Protein fractions were concentrated by ultrafiltration (Amicon Ultra, 30–50 kDa MWCO, Merck) and stored at −80 °C.

### Purification of MHC I^Fos^/TAPBPR^Jun^ complexes

HEK293 culture was cleared from cells by centrifugation at 2000×*g* for 10 min. Avidin (IBA Lifesciences, 7.5 mg/l) and Tris-HCl pH 8.0 (25 mM final) was added to the cleared supernatant. Affinity chromatography was performed with Strep-Tactin Sepharose (1 mL/L) (IBA Lifesciences) using 1xTBS buffer (25 mM Tris-HCl, pH 8.0, 100 mM NaCl). The tethered complex was eluted in 1xTBS supplemented with 5 mM desthiobiotin. Leucine zippers were removed by overnight digestion at 4 °C with Tobacco Etch Virus (TEV) protease. MHC I/TAPBR complexes were isolated either by size-exclusion chromatography (SEC) on a Superdex 200 Increase 10/300 column (Cytiva) in 1xHBS buffer and concentrated by ultrafiltration (Amicon Ultra 50 kDa MWCO, Merck) or subjected to UGGT-mediated reglucosylation before SEC purification.

### UGGT-mediated reglucosylation

MHC I/TAPBPR complexes (3 μM) were incubated with *Ct*UGGT (1 μM) in 1xHBS buffer supplemented with 200 μM UDP-glucose (UDP-Glc) and 5 mM CaCl_2_ for 10 min at RT. The reaction was stopped by adding 25 mM of EGTA, and MHC I-TAPBPR complexes were purified via SEC (Superdex 200 10/300 Increase, Cytiva) equilibrated in 1xHBS buffer. MHC I/TAPBPR peak fractions were pooled, flash-frozen in liquid nitrogen, and stored at −80 °C. Samples of the purified MHC I-TAPBPR complex were analyzed by LC-MS.

### Protein production of calreticulin variants

Crt and Crt^Δacidic^ were produced in *Escherichia coli* Rosetta(DE3)pLysS cells. In brief, cells were transformed with pETM-11 plasmids encoding Crt and Crt^Δacidic^. Overnight cultures of 10–20 mL LB media (10 g tryptone, 5 g yeast extract, 10 g NaCl per 1 l) supplemented with kanamycin were used to inoculate 2 L of LB main culture. The main culture was incubated at 37 °C and 180 rpm until an optical density at 600 nm (OD_600_) of 0.4–0.6 was reached. Temperature was reduced to 20 °C, and protein production was induced by the addition of 1 mM isopropyl-β-D-thiogalactopyranoside (IPTG). After 20 h, cells were harvested by centrifugation at 4 °C and 4500×*g* for 15 min. Cells were flash-frozen in liquid nitrogen and stored at −80 °C.

### Protein production in insect cells

The disulfide-linked tapasin-ERp57 heterodimer was produced in *Spodoptera frugiperda* (*Sf*21) cells as described (Müller et al, 2022). Cells were grown in *Sf*900 II SFM medium (Thermo Fisher Scientific) at 28 °C and transfected with modified EMBacY bacterial artificial chromosomes (BACs) (Trowitzsch et al, 2010) using X-tremeGENE DNA-transfection reagent (Roche). After incubation for 72 h at 28 °C, recombinant baculovirus V0 was harvested and utilized for the production of amplified baculovirus V1. The tapasin-ERp57 heterodimer was expressed in 800 mL suspension culture at a cell density of 10^6^ cells/mL by infection with 0.5–2.0% (v/v) baculovirus V1. Cells were harvested 72 h post cell proliferation arrest by centrifugation at 4 °C and 1300×*g* for 10 min. Cell pellets were flash-frozen in liquid nitrogen and stored at −80 °C.

### Purification of tapasin-ERp57 heterodimers

Infected *Sf*21 cell pellets were resuspended in 100 mL lysis buffer (50 mM HEPES-NaOH, pH 7.4, 150 mM NaCl, 25 mM imidazole, 1 mM phenylmethylsulfonyl fluoride (PMSF), 1 mM benzamidine) per one liter expression culture and lysed by sonication. After centrifugation at 20,000×*g*, 4 °C for 30 min, the supernatant was incubated with pre-equilibrated Ni^2+^-*N*-nitrilotriacetic acid (NTA) agarose resin (Thermo Fisher Scientific) for 1 h at 4 °C. The resin was washed with lysis buffer without protease inhibitors, and bound proteins were eluted with elution buffer (1xHBS supplemented with 300 mM of imidazole). Protein fractions were pooled, the His_6_-tag was cleaved, and the buffer was exchanged to 1xHBS buffer by dialysis overnight at 4 °C. Tapasin-ERp57 heterodimers were concentrated by ultrafiltration (Amicon Ultra 50 kDa, MWCO, Merck) and isolated by SEC (Superdex 200 10/300 Increase, Cytiva) equilibrated in 1xHBS buffer. Peak fractions were pooled, flash-frozen in liquid nitrogen, and stored at −80 °C.

### Purification of calreticulin variants

Cell pellets of *E. coli* Rosetta(DE3)pLysS containing expressed Crt^WT^ and Crt^Δacidic^, respectively, were resuspended in 100 mL lysis buffer (50 mM HEPES-NaOH pH 7.4, 150 mM NaCl, 25 mM imidazole, 5 mM CaCl_2_, 1 mM phenylmethylsulfonyl fluoride (PMSF), 1 mM benzamidine, Benzonase 1:10,000 (v:v) from Merck Millipore) per 1 l expression culture and lysed by sonication. The lysate was centrifuged for 30 min at 20,000×*g*, 4 °C, and the supernatant incubated with pre-equilibrated Ni^2+^-NTA agarose resin (Thermo Scientific) for 1 h at 4 °C. The Ni^2+^-NTA agarose resin was washed with lysis buffer without protease inhibitors and Benzonase. The immobilized protein was eluted with elution buffer (1xHBS supplemented with 300 mM of imidazole and 1 mM CaCl_2_). Protein fractions were pooled, the His_6_-tag was cleaved, and the buffer was exchanged to 1xHBS buffer supplemented with 1 mM CaCl_2_ by dialysis overnight at 4 °C. Crt^WT^ or Crt^Δacidic^, respectively, were concentrated by ultrafiltration (Amicon Ultra 10 kDa, MWCO, Merck) and isolated by SEC (Superdex 200 10/300 Increase, Cytiva) equilibrated in 1xHBS buffer supplemented with 1 mM CaCl_2_. Peak fractions were pooled, flash-frozen in liquid nitrogen, and stored at −80 °C.

### Purification of PaSta1 antibody fragment

Secreted PaSta1 Fab was purified from cleared HEK293 culture supernatant after centrifugation at 2000×*g* for 10 min at 4 °C. The cleared supernatant of HEK293-F was supplemented with Tris-HCl pH 8.0 (25 mM final), and affinity chromatography was performed with Ni^2+^-NTA agarose resin (Thermo Scientific). The resin was washed with 1xHBS buffer supplemented with 25 mM imidazole, and the bound protein was eluted with 1xHBS supplemented with 300 mM imidazole. Protein fractions were pooled, and the buffer was exchanged overnight by dialysis against 1xHBS buffer at 4 °C. PaSta1 Fab was isolated by SEC (Superdex 200 Increase 10/300, Cytiva) in 1xHBS buffer. Protein fractions were concentrated by ultrafiltration (Amicon Ultra, 10 kDa MWCO, Merck) and stored at −80 °C.

### Complex formation of various MHC I/chaperone complexes

SEC analyses of each component of the chaperone exchange reaction were performed with 3 µM of each protein used in the experiment, unless stated otherwise. Tapasin-ERp57 was pre-incubated with PaSta1 Fab in equimolar ratio for at least 15 min at 4 °C prior to experiments. Before the exchange experiments involving peptide-loaded HLA-A*68:02, the MHC I complex was incubated overnight at 4 °C with 250x molar excess of the high-affinity peptide ETVSKQSNV (predicted *K*_d_ of 16 nM (Reynisson et al, 2020)). Exchange experiments were performed by incubation of several exchange components for 30 min at 4 °C. SEC analyses were performed with a Superdex 200 3.2/300 Increase column (Cytiva) equilibrated with 1xHBS at a flow rate of 0.05 mL/min. At least 20 µL samples of each SEC fraction were gathered and analyzed by SDS-PAGE and immunoblotting.

### Liquid chromatography (LC)-MS analyses

LC-MS analyses were conducted on a BioAccord System (Waters). Top-down LC-MS data for glycan analysis were collected using a cone voltage of 45, 1.5 kV capillary voltage, and a desolvation temperature of 500 °C on an ACQUITY UPLC Protein BEH C4 Column, 300 Å, 1.7 μm, 2.1 × 50 mm, or 150 mm (Waters) at 80 °C applying a linear water/acetonitrile gradient (5–45% (v/v) acetonitrile in 8.0 min or 5–95% (v/v) acetonitrile in 66.5 min) supplemented with 0.1% (v/v) formic acid. Mass spectra were recorded in positive polarity at 2 Hz in full scan mode at 400–7000 m/z. Reglucosylation mixtures were spun down at 16,000×*g* for 10 min and directly analyzed by LC-MS. Glycoprotein masses were calculated and confirmed using the software platform UNIFY 3.1.0 (Waters). Combined intact mass spectra of glycoproteins were deconvoluted in UNIFY 3.1.0, utilizing the quantitative MaxEnt1 algorithm, iterating to convergence with 1.0 Da model width. Deconvoluted spectra were centroidized based on peak height and plotted in OriginPro 2022b (Origin Lab Corporation, USA). Of note, assignment of glycan compositions to mass peaks is based on observed masses and mass changes in Figs. 1B and 2D–F. LC-MS data were deposited in the GUDe data repository under the DOI accession number: https://doi.org/10.25716/gude.106c-n5yg.

### Laser-induced liquid bead ion desorption (LILBID)-MS analyses

Samples were prepared for native LILBID-MS analysis by performing a buffer exchange into 200 mM ammonium acetate, using Zeba Spin desalting columns (Thermo Fisher Scientific, USA). The samples, at concentrations of 3–10 µM, were loaded into the piezo-driven droplet generator (MD-K-130, Microdrop Technologies GmbH, Germany) operating at 10 Hz and generating 50 µm diameter droplets. Sample droplets were irradiated by an IR laser (a Nd:YAG pumped optical parametric oscillator (OPO)) with a wavelength of 2.8 µm in a high-vacuum irradiation chamber. A laser pulse of 6 ns pulse length was used to cause explosive expansion of the sample droplets, leading to the release of ionized molecules into a homebuilt time-of-flight analyzer by applying an acceleration voltage (2.6 kV). The procedure was performed with Wiley McLaren type ion optics, by setting both first and second lens to −4 kV and then ramping the first lens 5 to 25 µs after the explosion to −6.6 kV for 370 µs. A reflectron, working at −7.2 kV, guided the ions into the Daly type detector, optimized for high m/z. Data processing was performed by using OriginPro 2022b (Origin Lab Corporation, USA). LILBID-MS data were deposited in the GUDe data repository under the accession number: https://doi.org/10.25716/gude.18f9-c6gc.

### Isothermal titration calorimetry (ITC)

Thermodynamic parameters of the interactions between Crt and either the Tsn–ERp57^C36A^ heterodimer, or the MHC I/TAPBPR heterodimer were determined using a MicroCal PEAQ-ITC microcalorimeter (Malvern Panalytical). In a typical experiment, the titrant (500 μM) was loaded into a 40-μL injection syringe, and the analyte (20 μM) was placed in the 280 μL sample cell. Prior to measurements, all components were dialyzed against an identical buffer (50 mM HEPES-NaOH pH 7.4, 150 mM NaCl, and 1 mM CaCl_2_). Experiments were performed at 25 °C using 19 injections of 2 μL at 2.5 min intervals. The first injection (0.4 µL) was excluded from analysis. As a control, Crt was titrated into the buffer alone. Data were analyzed using MicroCal PEAQ-ITC Analysis Software (1.21) to determine equilibrium dissociation constants (*K*_d_), and stoichiometry (*n*). Protein concentrations were determined by absorbance at 280 nm using calculated extinction coefficients.

### SDS-PAGE and immunoblotting

6× sodium dodecyl sulfate (SDS)-loading buffer (150 mM Tris-HCl, pH 6.8, 30% (v/v) glycerol, 1.2% (w/v) SDS, 0.02% (w/v) Bromophenol Blue) was added to SDS-PAGE samples and incubated at 95 °C for 10 min. 10 μl of samples were loaded on 11% Tris-tricine gel, and gel electrophoresis was performed at 180 V for 45 min. The gels were either stained with InstantBlue Coomassie Protein Stain (abcam) or blotted onto a PVDF membrane (0.45 µm) by semi-dry blotting for 30 min at 25 V. The blotted membranes were blocked in 5% (w/v) milk powder in TBS-T for 1 h and incubated with an appropriate primary antibody at 4 °C overnight. MHC I hc was analyzed with the anti-HLA-A,B,C primary antibody HC10 (in-house production, hybridoma, mouse, 1:20 dilution) and Crt^WT^/Crt^Δacidic^ with the anti-Crt primary antibody FMC75 (abcam, mouse, 1:2000 dilution). Blots were washed three times in TBS-T at RT for 10 min each and then incubated with a corresponding second antibody-HRP conjugate (anti-mouse, A-2554 from Sigma-Aldrich, goat, 1:20,000) for 1 h at RT. After washing three times with TBS-T for 10 min each at RT, membranes were incubated with Clarity Western ECL reagent (Bio-Rad). Immunoblot signals were quantified by ImageJ (Schneider et al, 2012).

## Supplementary information

Appendix

Peer Review File

Source data Fig. 1

Source data Fig. 2

Source data Fig. 3

Source data Fig. 4

Source data Fig. 5

Figure EV1 Source Data

Figure EV2 Source Data

Figure EV3 Source Data

Figure EV4 Source Data

Figure EV5 Source Data

Expanded View Figures

## Data Availability

The datasets of this study are available in the following databases: SEC data: Goethe University Data Repository GUDe https://doi.org/10.25716/gude.18f9-c6gc. SDS-PAGE and corresponding immunoblots: Goethe University Data Repository GUDe https://doi.org/10.25716/gude.18f9-c6gc. LC-MS and LILBID data: Goethe University Data Repository GUDe https://doi.org/10.25716/gude.18f9-c6gc. ITC data: Goethe University Data Repository GUDe https://doi.org/10.25716/gude.18f9-c6gc. The source data of this paper are collected in the following database record: biostudies:S-SCDT-10_1038-S44318-026-00873-2.

## References

[CR1] Andreatta M, Nielsen M (2016) Gapped sequence alignment using artificial neural networks: application to the MHC class I system. Bioinformatics 32:511–517 10.1093/bioinformatics/btv639 PMC6402319 26515819

[CR2] Arshad N, Cresswell P (2018) Tumor-associated calreticulin variants functionally compromise the peptide loading complex and impair its recruitment of MHC-I. J Biol Chem 293:9555–9569 10.1074/jbc.RA118.002836 PMC6016473 29769311

[CR3] Arshad N, Cresswell P (2021) Impact of calreticulin and Its mutants on endoplasmic reticulum function in health and disease. Prog Mol Subcell Biol 59:163–180 10.1007/978-3-030-67696-4_8 34050866

[CR4] Barker MK, Rose DR (2013) Specificity of processing alpha-glucosidase I is guided by the substrate conformation: crystallographic and in silico studies. J Biol Chem 288:13563–13574 10.1074/jbc.M113.460436 PMC3650392 23536181

[CR5] Bashirova AA, Viard M, Naranbhai V, Grifoni A, Garcia-Beltran W, Akdag M, Yuki Y, Gao X, O’HUigin C, Raghavan M et al (2020) HLA tapasin independence: broader peptide repertoire and HIV control. Proc Natl Acad Sci USA 117:28232–28238 10.1073/pnas.2013554117 PMC7668082 33097667

[CR6] Blees A, Januliene D, Hofmann T, Koller N, Schmidt C, Trowitzsch S, Moeller A, Tampé R (2017) Structure of the human MHC-I peptide-loading complex. Nature 551:525–528 10.1038/nature24627 29107940

[CR7] Blum JS, Wearsch PA, Cresswell P (2013) Pathways of antigen processing. Annu Rev Immunol 31:443–473 10.1146/annurev-immunol-032712-095910 PMC4026165 23298205

[CR8] Boyle LH, Hermann C, Boname JM, Porter KM, Patel PA, Burr ML, Duncan LM, Harbour ME, Rhodes DA, Skjodt K et al (2013) Tapasin-related protein TAPBPR is an additional component of the MHC class I presentation pathway. Proc Natl Acad Sci USA 110:3465–3470 10.1073/pnas.1222342110 PMC3587277 23401559

[CR9] Breitling J, Aebi M (2013) N-linked protein glycosylation in the endoplasmic reticulum. Cold Spring Harb Perspect Biol 5:a013359 10.1101/cshperspect.a013359 PMC3721281 23751184

[CR10] Caputo AT, Alonzi DS, Marti L, Reca IB, Kiappes JL, Struwe WB, Cross A, Basu S, Lowe ED, Darlot B et al (2016) Structures of mammalian ER alpha-glucosidase II capture the binding modes of broad-spectrum iminosugar antivirals. Proc Natl Acad Sci USA 113:E4630–E4638 10.1073/pnas.1604463113 PMC4987793 27462106

[CR11] Chang VT, Crispin M, Aricescu AR, Harvey DJ, Nettleship JE, Fennelly JA, Yu C, Boles KS, Evans EJ, Stuart DI et al (2007) Glycoprotein structural genomics: solving the glycosylation problem. Structure 15:267–273 10.1016/j.str.2007.01.011 PMC1885966 17355862

[CR12] Chappell P, Meziane el K, Harrison M, Magiera L, Hermann C, Mears L, Wrobel AG, Durant C, Nielsen LL, Buus S et al (2015) Expression levels of MHC class I molecules are inversely correlated with promiscuity of peptide binding. eLife 4:e05345 10.7554/eLife.05345 PMC4420994 25860507

[CR13] Chen M, Bouvier M (2007) Analysis of interactions in a tapasin/class I complex provides a mechanism for peptide selection. EMBO J 26:1681–1690 10.1038/sj.emboj.7601624 PMC1829385 17332746

[CR14] Del Cid N, Jeffery E, Rizvi SM, Stamper E, Peters LR, Brown WC, Provoda C, Raghavan M (2010) Modes of calreticulin recruitment to the major histocompatibility complex class I assembly pathway. J Biol Chem 285:4520–4535 10.1074/jbc.M109.085407 PMC2836057 19959473

[CR15] Deprez P, Gautschi M, Helenius A (2005) More than one glycan is needed for ER glucosidase II to allow entry of glycoproteins into the calnexin/calreticulin cycle. Mol Cell 19:183–195 10.1016/j.molcel.2005.05.029 16039588

[CR16] Dick TP, Bangia N, Peaper DR, Cresswell P (2002) Disulfide bond isomerization and the assembly of MHC class I-peptide complexes. Immunity 16:87–98 10.1016/s1074-7613(02)00263-7 11825568

[CR17] Domnick A, Winter C, Sušac L, Hennecke L, Hensen M, Zitzmann N, Trowitzsch S, Thomas C, Tampé R (2022) Molecular basis of MHC I quality control in the peptide loading complex. Nat Commun 13:4701 10.1038/s41467-022-32384-z PMC9365787 35948544

[CR18] Dong G, Wearsch PA, Peaper DR, Cresswell P, Reinisch KM (2009) Insights into MHC class I peptide loading from the structure of the tapasin-ERp57 thiol oxidoreductase heterodimer. Immunity 30:21–32 10.1016/j.immuni.2008.10.018 PMC2650231 19119025

[CR19] Elbein AD (1991) Glycosidase inhibitors: inhibitors of N-linked oligosaccharide processing. FASEB J 5:3055–3063 10.1096/fasebj.5.15.1743438 1743438

[CR20] Elf S, Abdelfattah NS, Chen E, Perales-Paton J, Rosen EA, Ko A, Peisker F, Florescu N, Giannini S, Wolach O et al (2016) Mutant calreticulin requires both Its mutant C-terminus and the thrombopoietin receptor for oncogenic transformation. Cancer Discov 6:368–381 10.1158/2159-8290.CD-15-1434 PMC4851866 26951227

[CR21] Fleischmann G, Fisette O, Thomas C, Wieneke R, Tumulka F, Schneeweiss C, Springer S, Schäfer LV, Tampé R (2015) Mechanistic basis for epitope proofreading in the peptide-loading complex. J Immunol 195:4503–4513 10.4049/jimmunol.1501515 26416272

[CR22] Frickel EM, Riek R, Jelesarov I, Helenius A, Wuthrich K, Ellgaard L (2002) TROSY-NMR reveals interaction between ERp57 and the tip of the calreticulin P-domain. Proc Natl Acad Sci USA 99:1954–1959 10.1073/pnas.042699099 PMC122301 11842220

[CR23] Gao B, Adhikari R, Howarth M, Nakamura K, Gold MC, Hill AB, Knee R, Michalak M, Elliott T (2002) Assembly and antigen-presenting function of MHC class I molecules in cells lacking the ER chaperone calreticulin. Immunity 16:99–109 10.1016/s1074-7613(01)00260-6 11825569

[CR24] Garstka M, Borchert B, Al-Balushi M, Praveen PV, Kuhl N, Majoul I, Duden R, Springer S (2007) Peptide-receptive major histocompatibility complex class I molecules cycle between endoplasmic reticulum and cis-Golgi in wild-type lymphocytes. J Biol Chem 282:30680–30690 10.1074/jbc.M701721200 17656363

[CR25] Gemmer M, Chaillet ML, van Loenhout J, Cuevas Arenas R, Vismpas D, Grollers-Mulderij M, Koh FA, Albanese P, Scheltema RA, Howes SC et al (2023) Visualization of translation and protein biogenesis at the ER membrane. Nature 614:160–167 10.1038/s41586-022-05638-5 PMC9892003 36697828

[CR26] Guay KP, Chou WC, Canniff NP, Paul KB, Hebert DN (2025) N-glycan-dependent protein maturation and quality control in the ER. Nat Rev Mol Cell Biol 26:926–939 10.1038/s41580-025-00855-y PMC13091552 40389697

[CR27] Helenius A, Aebi M (2001) Intracellular functions of N-linked glycans. Science 291:2364–2369 10.1126/science.291.5512.2364 11269317

[CR28] Helenius A, Aebi M (2004) Roles of N-linked glycans in the endoplasmic reticulum. Annu Rev Biochem 73:1019–1049 10.1146/annurev.biochem.73.011303.073752 15189166

[CR29] Hermann C, Strittmatter LM, Deane JE, Boyle LH (2013) The binding of TAPBPR and Tapasin to MHC class I is mutually exclusive. J Immunol 191:5743–5750 10.4049/jimmunol.1300929 PMC3836178 24163410

[CR30] Hermann C, van Hateren A, Trautwein N, Neerincx A, Duriez PJ, Stevanovic S, Trowsdale J, Deane JE, Elliott T, Boyle LH (2015) TAPBPR alters MHC class I peptide presentation by functioning as a peptide exchange catalyst. eLife 10.7554/eLife.09617 PMC4718805 26439010

[CR31] Howarth M, Williams A, Tolstrup AB, Elliott T (2004) Tapasin enhances MHC class I peptide presentation according to peptide half-life. Proc Natl Acad Sci USA 101:11737–11742 10.1073/pnas.0306294101 PMC511045 15286279

[CR32] Howe C, Garstka M, Al-Balushi M, Ghanem E, Antoniou AN, Fritzsche S, Jankevicius G, Kontouli N, Schneeweiss C, Williams A et al (2009) Calreticulin-dependent recycling in the early secretory pathway mediates optimal peptide loading of MHC class I molecules. EMBO J 28:3730–3744 10.1038/emboj.2009.296 PMC2790484 19851281

[CR33] Ilca FT, Drexhage LZ, Brewin G, Peacock S, Boyle LH (2019) Distinct polymorphisms in HLA class I molecules govern their susceptibility to peptide editing by TAPBPR. Cell Rep 29:1621–1632.e1623 10.1016/j.celrep.2019.09.074 PMC7057265 31693900

[CR34] Jiang J, Natarajan K, Boyd LF, Morozov GI, Mage MG, Margulies DH (2017) Crystal structure of a TAPBPR-MHC I complex reveals the mechanism of peptide editing in antigen presentation. Science 358:1064–1068 10.1126/science.aao5154 PMC6320693 29025991

[CR35] Jiang J, Taylor DK, Kim EJ, Boyd LF, Ahmad J, Mage MG, Truong HV, Woodward CH, Sgourakis NG, Cresswell P et al (2022) Structural mechanism of tapasin-mediated MHC-I peptide loading in antigen presentation. Nat Commun 13:5470 10.1038/s41467-022-33153-8 PMC9482634 36115831

[CR36] Kapoor M, Ellgaard L, Gopalakrishnapai J, Schirra C, Gemma E, Oscarson S, Helenius A, Surolia A (2004) Mutational analysis provides molecular insight into the carbohydrate-binding region of calreticulin: pivotal roles of tyrosine-109 and aspartate-135 in carbohydrate recognition. Biochemistry 43:97–106 10.1021/bi0355286 14705935

[CR37] Kapoor M, Srinivas H, Kandiah E, Gemma E, Ellgaard L, Oscarson S, Helenius A, Surolia A (2003) Interactions of substrate with calreticulin, an endoplasmic reticulum chaperone. J Biol Chem 278:6194–6200 10.1074/jbc.M209132200 12464625

[CR38] Klampfl T, Gisslinger H, Harutyunyan AS, Nivarthi H, Rumi E, Milosevic JD, Them NC, Berg T, Gisslinger B, Pietra D et al (2013) Somatic mutations of calreticulin in myeloproliferative neoplasms. N Engl J Med 369:2379–2390 10.1056/NEJMoa1311347 24325356

[CR39] Kozlov G, Gehring K (2020) Calnexin cycle - structural features of the ER chaperone system. FEBS J 287:4322–4340. 10.1111/febs.15330 PMC7687155 32285592

[CR40] Kozlov G, Maattanen P, Schrag JD, Pollock S, Cygler M, Nagar B, Thomas DY, Gehring K (2006) Crystal structure of the bb’ domains of the protein disulfide isomerase ERp57. Structure 14:1331–1339 10.1016/j.str.2006.06.019 16905107

[CR41] Kozlov G, Pocanschi CL, Rosenauer A, Bastos-Aristizabal S, Gorelik A, Williams DB, Gehring K (2010) Structural basis of carbohydrate recognition by calreticulin. J Biol Chem 285:38612–38620 10.1074/jbc.M110.168294 PMC2992293 20880849

[CR42] Lan BH, Becker M, Freund C (2023) The mode of action of tapasin on major histocompatibility class I (MHC-I) molecules. J Biol Chem 299:102987 10.1016/j.jbc.2023.102987 PMC10040737 36758805

[CR43] Morozov GI, Zhao H, Mage MG, Boyd LF, Jiang J, Dolan MA, Venna R, Norcross MA, McMurtrey CP, Hildebrand W et al (2016) Interaction of TAPBPR, a tapasin homolog, with MHC-I molecules promotes peptide editing. Proc Natl Acad Sci USA 113:E1006–E1015 10.1073/pnas.1519894113 PMC4776512 26869717

[CR44] Müller IK, Winter C, Thomas C, Spaapen RM, Trowitzsch S, Tampé R (2022) Structure of an MHC I-tapasin-ERp57 editing complex defines chaperone promiscuity. Nat Commun 13:5383 10.1038/s41467-022-32841-9 PMC9474470 36104323

[CR45] Nangalia J, Massie CE, Baxter EJ, Nice FL, Gundem G, Wedge DC, Avezov E, Li J, Kollmann K, Kent DG et al (2013) Somatic CALR mutations in myeloproliferative neoplasms with nonmutated JAK2. N Engl J Med 369:2391–2405 10.1056/NEJMoa1312542 PMC3966280 24325359

[CR46] Neerincx A, Boyle LH (2019) Preferential interaction of MHC class I with TAPBPR in the absence of glycosylation. Mol Immunol 113:58–66 10.1016/j.molimm.2018.06.269 PMC6859791 30077416

[CR47] Neerincx A, Hermann C, Antrobus R, van Hateren A, Cao H, Trautwein N, Stevanovic S, Elliott T, Deane JE, Boyle LH (2017) TAPBPR bridges UDP-glucose:glycoprotein glucosyltransferase 1 onto MHC class I to provide quality control in the antigen presentation pathway. eLife 10.7554/eLife.23049 PMC5441866 28425917

[CR48] Ninagawa S, Matsuo M, Ying D, Oshita S, Aso S, Matsushita K, Taniguchi M, Fueki A, Yamashiro M, Sugasawa K et al (2024) UGGT1-mediated reglucosylation of N-glycan competes with ER-associated degradation of unstable and misfolded glycoproteins. eLife 10.7554/eLife.93117.2 PMC11630818 39654396

[CR49] O’Rourke SM, Morozov GI, Roberts JT, Barb AW, Sgourakis NG (2019) Production of soluble pMHC-I molecules in mammalian cells using the molecular chaperone TAPBPR. Protein Eng Des Sel 32:525–532 10.1093/protein/gzaa015 PMC7451022 32725167

[CR50] Parodi AJ, Mendelzon DH, Lederkremer GZ (1983) Transient glucosylation of protein-bound Man9GlcNAc2, Man8GlcNAc2, and Man7GlcNAc2 in calf thyroid cells. A possible recognition signal in the processing of glycoproteins. J Biol Chem 258:8260–8265 6863289

[CR51] Pishesha N, Harmand TJ, Ploegh HL (2022) A guide to antigen processing and presentation. Nat Rev Immunol 22:751–764 10.1038/s41577-022-00707-2 35418563

[CR52] Purcell AW, Gorman JJ, Garcia-Peydro M, Paradela A, Burrows SR, Talbo GH, Laham N, Peh CA, Reynolds EC, Lopez De Castro JA et al (2001) Quantitative and qualitative influences of tapasin on the class I peptide repertoire. J Immunol 166:1016–1027 10.4049/jimmunol.166.2.1016 11145681

[CR53] Reynisson B, Alvarez B, Paul S, Peters B, Nielsen M (2020) NetMHCpan-4.1 and NetMHCIIpan-4.0: improved predictions of MHC antigen presentation by concurrent motif deconvolution and integration of MS MHC eluted ligand data. Nucleic Acids Res 48:W449–W454 10.1093/nar/gkaa379 PMC7319546 32406916

[CR54] Rizvi SM, Salam N, Geng J, Qi Y, Bream JH, Duggal P, Hussain SK, Martinson J, Wolinsky SM, Carrington M et al (2014) Distinct assembly profiles of HLA-B molecules. J Immunol 192:4967–4976 10.4049/jimmunol.1301670 PMC4117407 24790147

[CR55] Roversi P, Marti L, Caputo AT, Alonzi DS, Hill JC, Dent KC, Kumar A, Levasseur MD, Lia A, Waksman T et al (2017) Interdomain conformational flexibility underpins the activity of UGGT, the eukaryotic glycoprotein secretion checkpoint. Proc Natl Acad Sci USA 114:8544–8549 10.1073/pnas.1703682114 PMC5559018 28739903

[CR56] Sadasivan B, Lehner PJ, Ortmann B, Spies T, Cresswell P (1996) Roles for calreticulin and a novel glycoprotein, tapasin, in the interaction of MHC class I molecules with TAP. Immunity 5:103–114 10.1016/s1074-7613(00)80487-2 8769474

[CR57] Sagert L, Hennig F, Thomas C, Tampé R (2020) A loop structure allows TAPBPR to exert its dual function as MHC I chaperone and peptide editor. eLife 10.7554/eLife.55326 PMC7117912 32167472

[CR58] Sagert L, Winter C, Ruppert I, Zehetmaier M, Thomas C, Tampé R (2023) The ER folding sensor UGGT1 acts on TAPBPR-chaperoned peptide-free MHC I. eLife 10.7554/eLife.85432 PMC10325711 37345806

[CR59] Schneider CA, Rasband WS, Eliceiri KW (2012) NIH Image to ImageJ: 25 years of image analysis. Nat Methods 9:671–675 10.1038/nmeth.2089 PMC5554542 22930834

[CR60] Sousa M, Parodi AJ (1995) The molecular basis for the recognition of misfolded glycoproteins by the UDP-Glc:glycoprotein glucosyltransferase. EMBO J 14:4196–4203 10.1002/j.1460-2075.1995.tb00093.x PMC394502 7556060

[CR61] Sousa MC, Ferrero-Garcia MA, Parodi AJ (1992) Recognition of the oligosaccharide and protein moieties of glycoproteins by the UDP-Glc:glycoprotein glucosyltransferase. Biochemistry 31:97–105 10.1021/bi00116a015 1531024

[CR62] Stolz M, Sušac L, Fahim A, Keller R, Mancia F, Trowitzsch S, Tampé R (2026) Architectural principles of transporter-chaperone coupling within the native MHC I peptide-loading complex. Sci Adv 12:eaea7735 10.1126/sciadv.aea7735 PMC12758552 41481733

[CR63] Sun Y, Papadaki GF, Devlin CA, Danon JN, Young MC, Winters TJ, Burslem GM, Procko E, Sgourakis NG (2023) Xeno interactions between MHC-I proteins and molecular chaperones enable ligand exchange on a broad repertoire of HLA allotypes. Sci Adv 9:eade7151 10.1126/sciadv.ade7151 PMC9956121 36827371

[CR64] Tan P, Kropshofer H, Mandelboim O, Bulbuc N, Hammerling GJ, Momburg F (2002) Recruitment of MHC class I molecules by tapasin into the transporter associated with antigen processing-associated complex is essential for optimal peptide loading. J Immunol 168:1950–1960 10.4049/jimmunol.168.4.1950 11823531

[CR65] Thomas C, Tampé R (2017) Structure of the TAPBPR-MHC I complex defines the mechanism of peptide loading and editing. Science 358:1060–1064 10.1126/science.aao6001 29025996

[CR66] Trowitzsch S, Bieniossek C, Nie Y, Garzoni F, Berger I (2010) New baculovirus expression tools for recombinant protein complex production. J Struct Biol 172:45–54 10.1016/j.jsb.2010.02.010 20178849

[CR67] Trowitzsch S, Tampé R (2020) Multifunctional chaperone and quality control complexes in adaptive immunity. Annu Rev Biophys 49:135–161 10.1146/annurev-biophys-121219-081643 32004089

[CR68] Wearsch PA, Cresswell P (2007) Selective loading of high-affinity peptides onto major histocompatibility complex class I molecules by the tapasin-ERp57 heterodimer. Nat Immunol 8:873–881 10.1038/ni1485 17603487

[CR69] Wearsch PA, Jakob CA, Vallin A, Dwek RA, Rudd PM, Cresswell P (2004) Major histocompatibility complex class I molecules expressed with monoglucosylated N-linked glycans bind calreticulin independently of their assembly status. J Biol Chem 279:25112–25121 10.1074/jbc.M401721200 15056662

[CR70] Wearsch PA, Peaper DR, Cresswell P (2011) Essential glycan-dependent interactions optimize MHC class I peptide loading. Proc Natl Acad Sci USA 108:4950–4955 10.1073/pnas.1102524108 PMC3064348 21383180

[CR71] Williams AP, Peh CA, Purcell AW, McCluskey J, Elliott T (2002) Optimization of the MHC class I peptide cargo is dependent on tapasin. Immunity 16:509–520 10.1016/s1074-7613(02)00304-7 11970875

[CR72] Zhang W, Wearsch PA, Zhu Y, Leonhardt RM, Cresswell P (2011) A role for UDP-glucose glycoprotein glucosyltransferase in expression and quality control of MHC class I molecules. Proc Natl Acad Sci USA 108:4956–4961 10.1073/pnas.1102527108 PMC3064381 21383159

