# Supplementary material for: Reconstitution of glycan-driven MHC-I recycling reveals calreticulin as mediator between TAPBPR and tapasin
Source: EMBO J. 2026 Aug 17;45(19):6699–722. doi: 10.1038/s44318-026-00873-2 (PMC13631358; doi:10.1038/s44318-026-00873-2)
Supplement: Supplementary file 1 [file 44318_2026_873_MOESM13_ESM.pdf]

Expanded View Figures

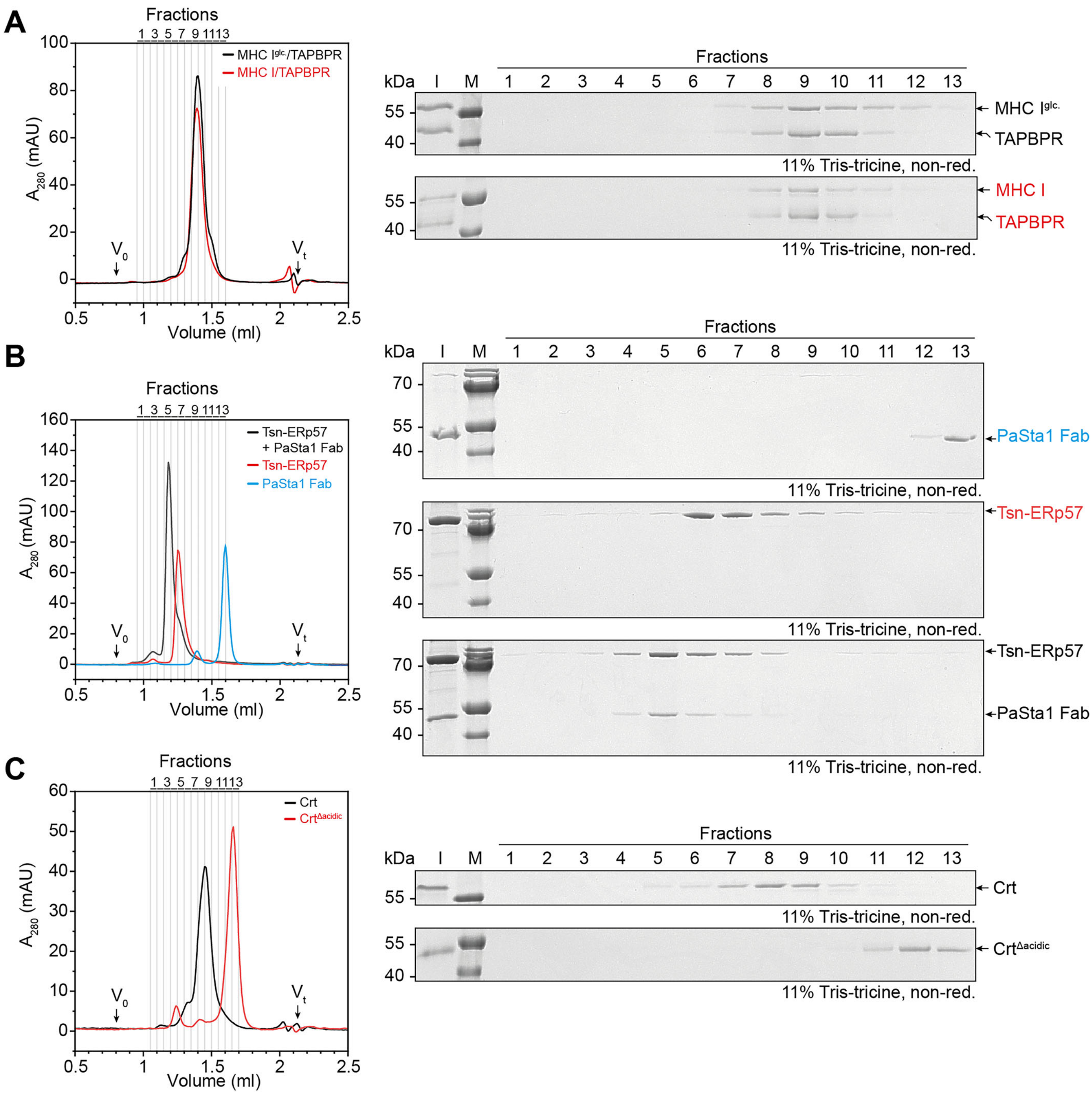

Figure EV1. Analysis of chaperone-MHC I complexes and their individual components by SEC.

(**A**) SEC profiles (left) and SDS-PAGE analyses of indicated fractions (right) for the single-chain β_2m-HLA-A*68:02/TAPBPR complex (3 μM, black line) or the monoglucosylated complex (3 μM, red line). (**B**) SEC profiles (left) and SDS-PAGE analysis of indicated fractions (right) for PaSta1 Fab alone (3 μM, blue line), soluble tapasin-ERp57 (3 μM, red line), and the tapasin-ERp57/PaSta1 complex (3 μM, black line). (**C**) SEC profiles (left) and SDS-PAGE analysis of indicated fractions (right) for calreticulin (Crt, 3 μM, black line) and its deletion variant ($Crt^{\Delta acidic}$, 3 μM, red line). M marker, I input, kDa kilodalton, A_{280} absorption at 280 nm. Data shown in panels (**A–C**) are representative of three independent experiments (*n* = 3). Source data are available online for this figure.

© The Author(s)

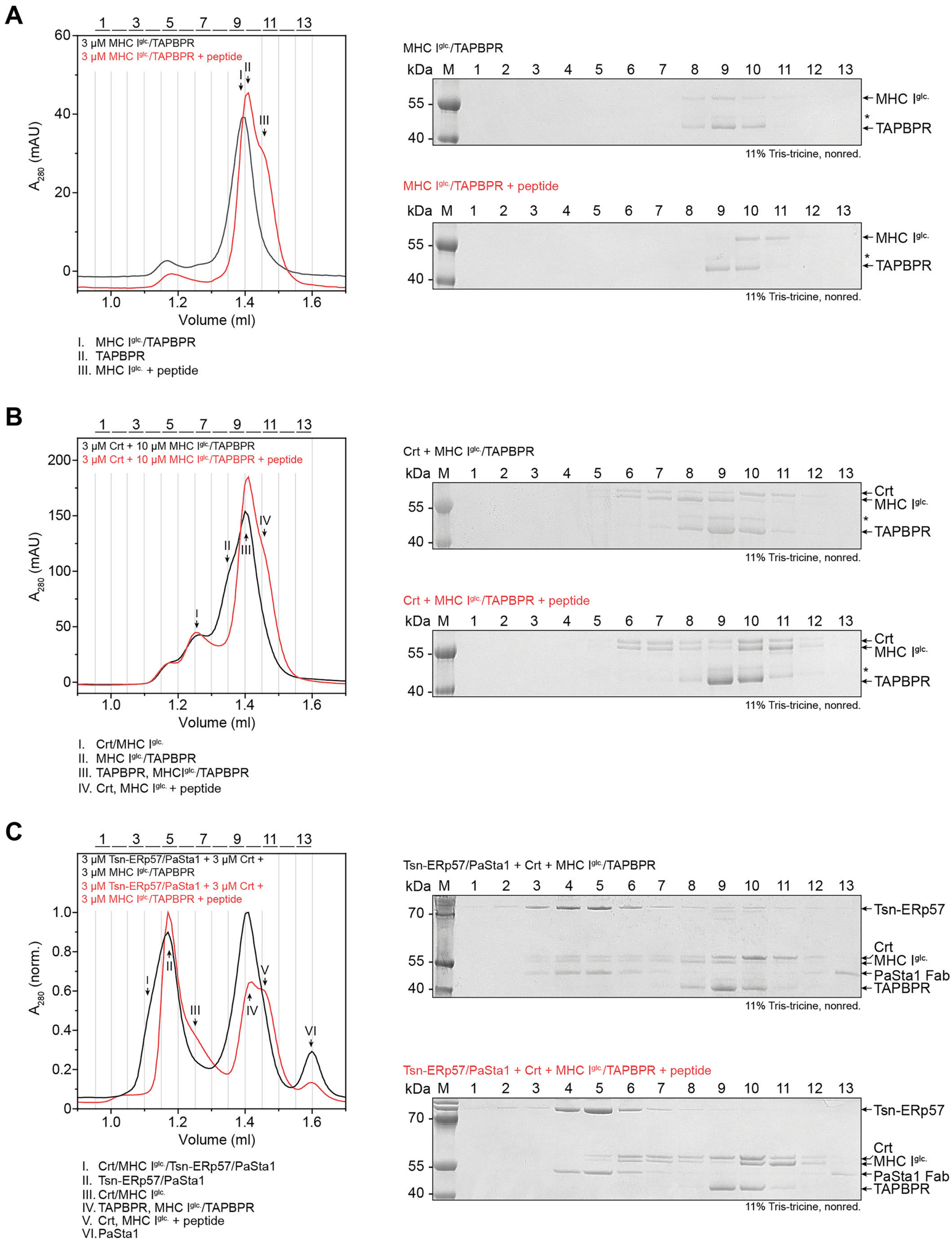
A
3 µM MHC Iglc./TAPBPR
3 µM MHC Iglc./TAPBPR + peptide
A280 (mAU)
Volume (ml)
I. MHC Iglc./TAPBPR
II. TAPBPR
III. MHC Iglc. + peptide
MHC Iglc./TAPBPR
kDa M 1 2 3 4 5 6 7 8 9 10 11 12 13
MHC Iglc.
TAPBPR
11% Tris-tricine, nonred.
MHC Iglc./TAPBPR + peptide
B
3 µM Crt + 10 µM MHC Iglc./TAPBPR
3 µM Crt + 10 µM MHC Iglc./TAPBPR + peptide
I. Crt/MHC Iglc.
II. MHC Iglc./TAPBPR
III. TAPBPR, MHCIglc./TAPBPR
IV. Crt, MHC Iglc. + peptide
Crt + MHC Iglc./TAPBPR
Crt + MHC Iglc./TAPBPR + peptide
Crt
C
3 µM Tsn-ERp57/PaSta1 + 3 µM Crt + 3 µM MHC Iglc./TAPBPR
3 µM Tsn-ERp57/PaSta1 + 3 µM Crt + 3 µM MHC Iglc./TAPBPR + peptide
A280 (norm.)
I. Crt/MHC Iglc./Tsn-ERp57/PaSta1
II. Tsn-ERp57/PaSta1
III. Crt/MHC Iglc.
IV. TAPBPR, MHC Iglc./TAPBPR
V. Crt, MHC Iglc. + peptide
VI. PaSta1
Tsn-ERp57/PaSta1 + Crt + MHC Iglc./TAPBPR
Tsn-ERp57/PaSta1 + Crt + MHC Iglc./TAPBPR + peptide
Tsn-ERp57
PaSta1 Fab

 © The Author(s)

Figure EV2. Influence of high-affinity peptides on MHC I quality control and recycling complexes.

(**A**) SEC profiles (left) and SDS-PAGE analysis of indicated fractions (right) for the monoglucosylated β_2m-HLA-A*68:02/TAPBPR complex (3 μM) in the presence (red line) or absence (black line) of peptide ETVSKQSNV (0.75 mM). (**B**) SEC profiles (left) and SDS-PAGE analysis (right) of mixtures of calreticulin (Crt, 3 μM) with monoglucosylated single-chain β_2m-HLA-A*68:02/TAPBPR complex (10 μM) in the presence (red line) or absence (black line) of peptide ETVSKQSNV (2.5 mM). (**C**) SEC profiles (left) and SDS-PAGE analysis (right) of mixtures of tapasin-ERp57/PaSta1 Fab (3 μM), monoglucosylated single-chain β_2m-HLA-A*68:02/TAPBPR complex, and Crt with (red line) or without (black line) peptide ETVSKQSNV (0.75 mM). Note that SEC profiles and corresponding SDS-PAGE analyses shown in panels (**B**, **C**) are identical to those in Figs. 3B, C and 1C, E, respectively. Image shown in panel (**B**) was taken from the same gel as shown in Fig. 3C, but at a different exposure time. Roman numbers above each peak correspond to the proteins or protein complexes defined below. M marker, I input, kDa kilodalton, A_{280} absorption at 280 nm. Data shown in panels (**A**–**C**) are representative of two independent experiments ($n = 2$) with peptide and three independent experiments ($n = 3$) without peptide. Source data are available online for this figure.

© The Author(s)

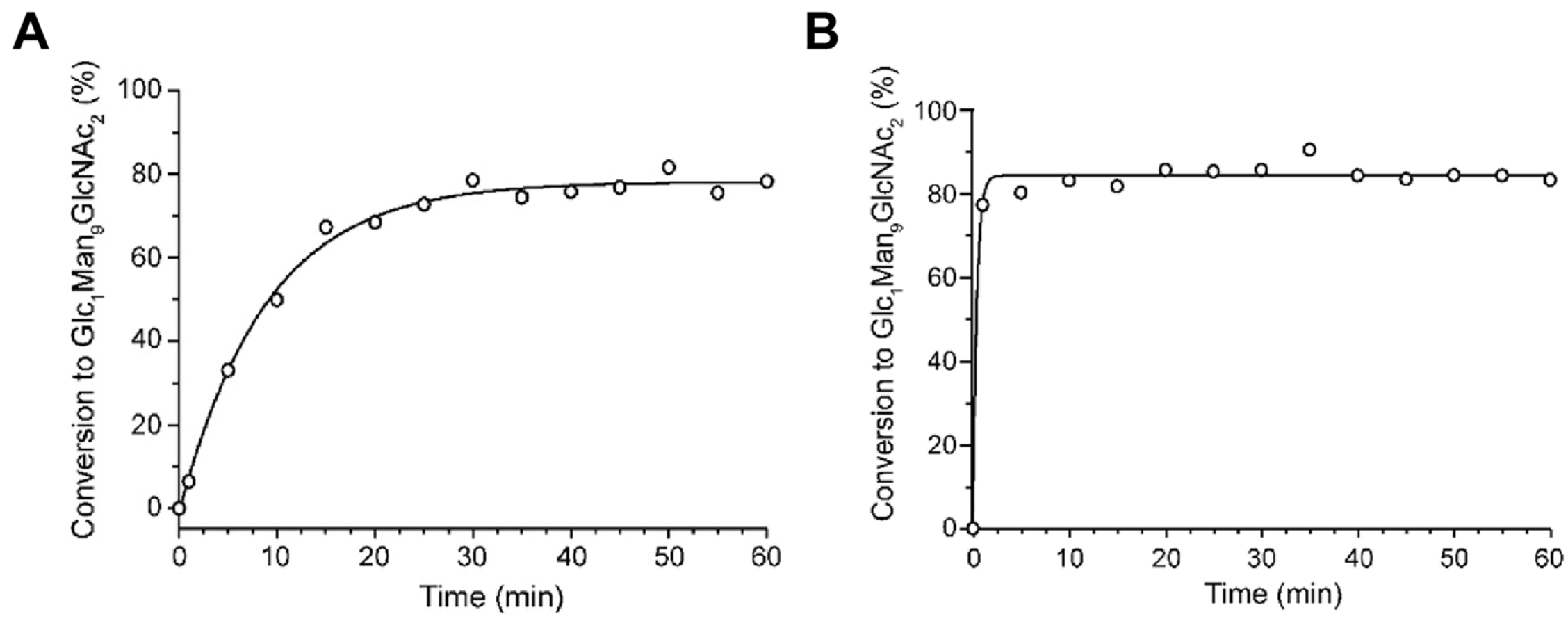

Figure EV3. Comparison of the monoglucosylation capacity of human UGGT1 and *Chaetomium thermophilum* UGGT on MHC I/TAPBPR complexes.

(**A**) Human UGGT1 (*Hs*UGGT1, 1 µM) (Sagert et al, 2023) or (**B**) *Chaetomium thermophilum* UGGT (*Ct*UGGT, 1 µM) was incubated with MHC I/TAPBPR complexes (3 µM) in 1× HBS buffer supplemented with UDP-glucose (UDP-Glc; 200 µM) and $CaCl_2$ (5 mM) at room temperature. Conversion to the monoglucosylated $Glc_1Man_9GlcNAc_2$ glycan (MHC I$^{glc.}$) was analyzed by LC-MS. Source data are available online for this figure.

© The Author(s)

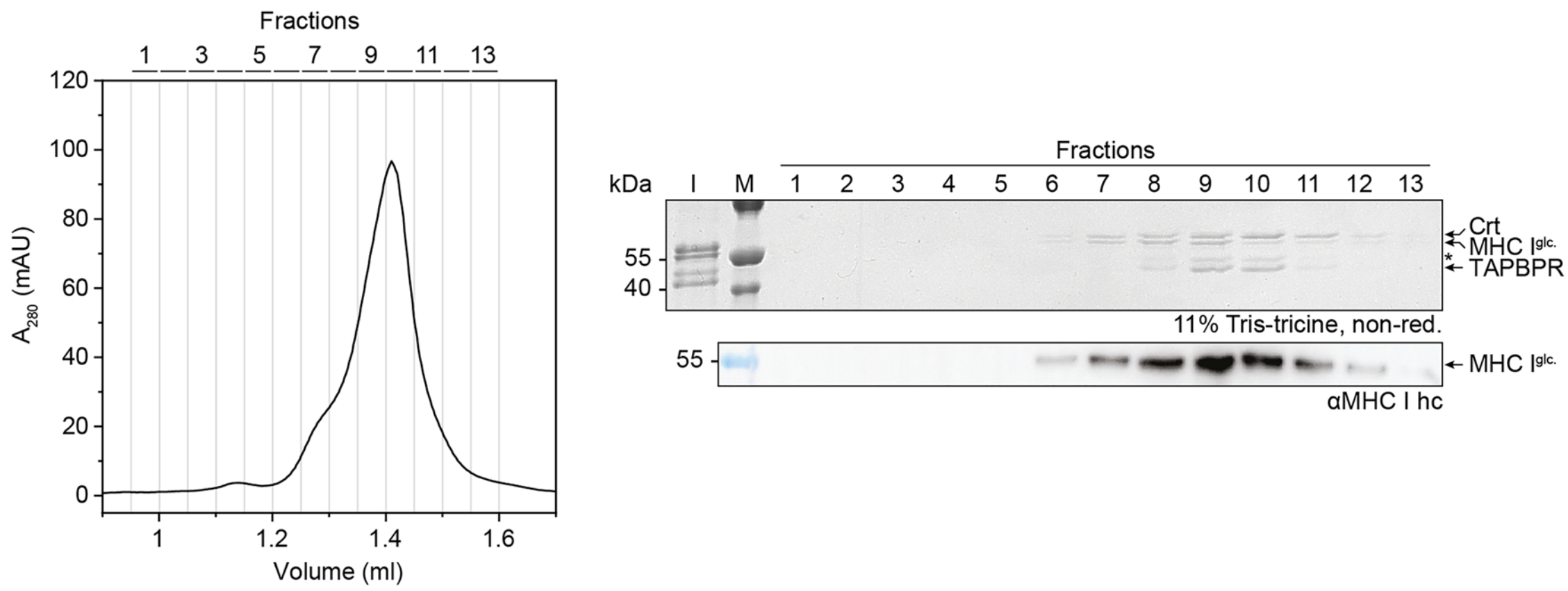

Figure EV4. Chaperone exchange of MHC I at equimolar concentrations of MHC $I^{glc.}$/TAPBPR and calreticulin.

Size-exclusion chromatography (SEC) profiles (left) with SDS-PAGE and immunoblot analyses (right) of monoglucosylated single-chain β_2m-HLA-A*68:02/TAPBPR complex and calreticulin (Crt) (3 µM each). The HC10 antibody was used to detect the MHC I heavy chain (αMHC I hc). M marker, I input, kDa kilodalton. Data shown in panels are representative of three independent experiments ($n = 3$). Source data are available online for this figure.

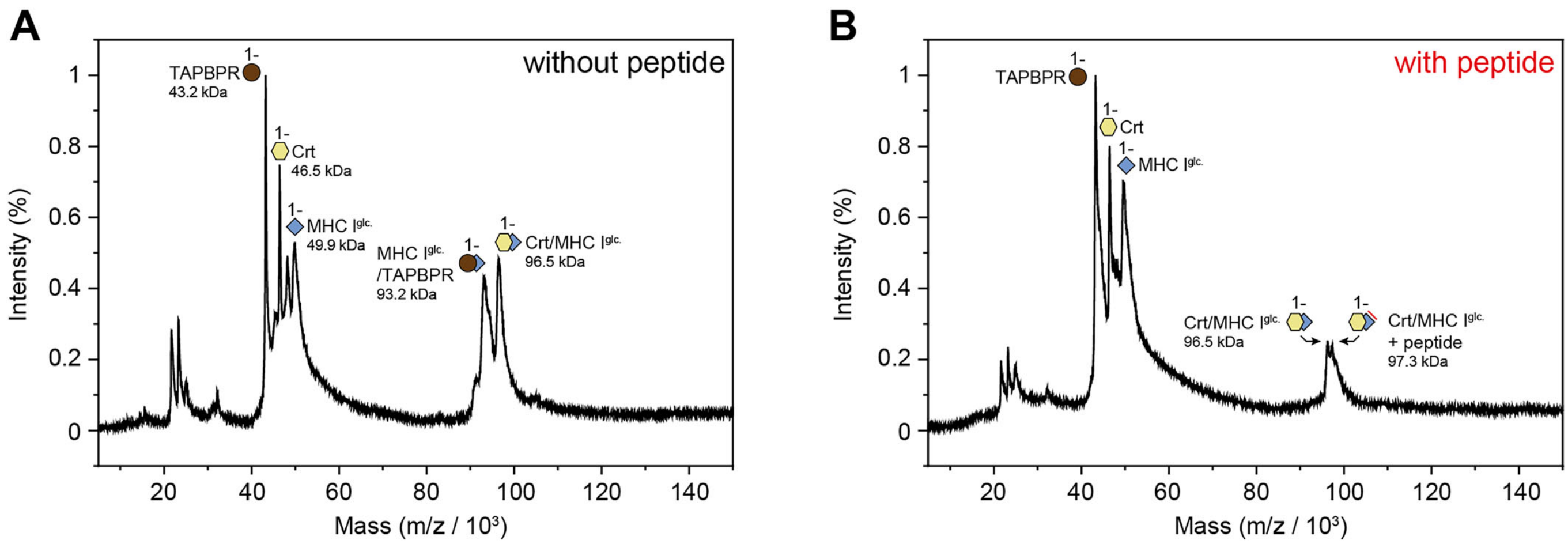

Figure EV5. High-affinity peptide induces disengagement of MHC I from TAPBPR.

(**A**, **B**) LILBID mass spectra of complexes formed upon mixture of monoglucosylated β_2m-HLA-A*68:02/TAPBPR complex (10 μM) with calreticulin (Crt; 3 μM) in the absence (**A**) or presence (**B**) of peptide ETVSKQSNV (2.5 mM). Charge states of the individual proteins or complexes and the corresponding observed masses are indicated. Brown circle – TAPBPR; yellow hexagon – Crt; blue square – MHC I$^{glc.}$. Source data are available online for this figure.

© The Author(s)
