# Supplementary material for: Reconstitution of glycan-driven MHC-I recycling reveals calreticulin as mediator between TAPBPR and tapasin
Source: EMBO J. 2026 Aug 17;45(19):6699–722. doi: 10.1038/s44318-026-00873-2 (PMC13631358; doi:10.1038/s44318-026-00873-2)
Supplement: Supplementary file 2 [file 44318_2026_873_MOESM1_ESM.pdf]

Appendix for:

Reconstitution of glycan-driven MHC-I recycling reveals calreticulin as mediator between TAPBPR and tapasin

This PDF file includes:

Appendix Table S1 Page 1

Appendix Table S1. Mass determination of MHC I species by LC-MS. List of observed masses ($M_{obs.}$) of glycosylated β_2m-HLA-A*68:02 single chain constructs by LC-MS with their corresponding calculated masses ($M_{calc.}$) related to Fig. 1 *B* and Fig. 2 *D-F*. Glc – Glucose, Man – Mannose, GlcNAc – N-acetylglucosamine, Da – Dalton.

MHC I species (N-glycan)	$M_{calc.}$ (Da)	$M_{obs.}$ (Da)				
		Fig. 1B (untreated)	Fig. 1B (UGGT-treated)	Fig. 2D	Fig. 2E	Fig. 2F
**$Man_6GlcNAc_2$**	49,674.64	-	-	49,674.73	49,674.48	-
**$Man_7GlcNAc_2$**	49,836.69	49,836.73	49,836.91	49,836.73	49,836.73	-
**$Man_8GlcNAc_2$**	49,998.74	49,998.82	49,998.71	49,998.76	49,998.58	-
**$Glc_1Man_8GlcNAc_2$ /$Man_9GlcNAc_2$**	50,160.79	50,161.19	50,160.97	50,161.12	50,161.62	50,160.86
**$Glc_1Man_9GlcNAc_2$**	50,322.84	-	50,322.89	50,322.70	-	50,323.01
