# Supplementary material for: Reconstitution of glycan-driven MHC-I recycling reveals calreticulin as mediator between TAPBPR and tapasin
Source: EMBO J. 2026 Aug 17;45(19):6699–722. doi: 10.1038/s44318-026-00873-2 (PMC13631358; doi:10.1038/s44318-026-00873-2)
Supplement: Supplementary file 3 [file 44318_2026_873_MOESM2_ESM.pdf]

Reconstitution of glycan-driven MHC-I recycling reveals calreticulin as mediator between TAPBPR and tapasin

Tim Heinke, Amin Fahim, Niko Popovic, Tobias Rath, Ines Müller, Nina Morgner, Simon Trowitzsch, and Robert Tampe

Corresponding author(s): Robert Tampe (tampe@em.uni-frankfurt.de) , Simon Trowitzsch (Trowitzsch@biochem.uni-frankfurt.de)

Review Timeline:

Submission Date:	23rd Dec 25
Editorial Decision:	25th Feb 26
Revision Received:	23rd May 26
Editorial Decision:	19th Jun 26
Revision Received:	27th Jun 26
Accepted:	29th Jun 26

Editor: Ioannis Papaioannou

Transaction Report:

(Note: With the exception of the correction of typographical or spelling errors that could be a source of ambiguity, letters and reports are not edited. Depending on transfer agreements, referee reports obtained elsewhere may or may not be included in this compilation. Referee reports are anonymous unless the Referee chooses to sign their reports.)

1st Editorial Decision **25th Feb 2026**

Dear Prof. Tampe,

Thank you for submitting your manuscript EMBOJ-2025-123404 for consideration by The EMBO Journal, and for your patience during peer review. Your manuscript has now been seen by three experts in the field, and we have received the complete set of their comments, which you can find appended below.

As you will see, referees #1 and #2 recognize the significance of the study and the advance provided by the manuscript. Referee #3, on the other hand, raises concerns regarding the novelty of the findings, although they do see value in the direct reconstitution of the transfer mechanism. In addition, the referees list a few specific points for strengthening the manuscript further and increasing its impact on the field.

On balance, and following the additional supportive input we received from the referees during a pre-decision cross-referee discussion, I would like to invite you to submit a revised version of your manuscript taking the referees' suggestions on board, along with a detailed point-by-point response addressing all referees' comments. In particular, I would like to note regarding the specific comments of the referees:
- re. point 1 of referee #1: all data generated in this study must be deposited in public repositories and listed in a Data availability statement (see also my detailed instructions below for more information)
- re. point 2 of referee #1: the point can be textually addressed, no experimental data are required
- re. point 1 of referee #2: we agree with the referee that the study would benetit significantly from the determination of the affinity constants as per the referee's suggestion
- re. points 2 and 3 of referee #2: the points can be textually addressed, no further experimentation in these directions will be required for this manuscript
- re. points 1, 2, 4, and 5 of referee #3: these minor points should be fully addressed/clarified in the revised version
- re. point 3 of referee #3: we agree with the referee that validation using disease-associated mutant would be desirable, if possible, but not strictly required for further consideration of the present manuscript.

Please note that it is The EMBO Journal policy to allow only a single round of revision, and acceptance of your manuscript will therefore depend on the completeness of your responses in this revised version.

We generally allow three months as standard revision time (24 May, 2026). As a matter of policy, competing manuscripts published during this period will not negatively impact our assessment of the conceptual advance presented by your study. However, we request that you contact us as soon as possible upon publication of any related work, to discuss how to proceed. Should you foresee a problem in meeting this three-month deadline, please let us know in advance and we will be able to grant an extension.

Please let me know if you have any questions or comments that you would like to discuss with me. Thank you for the opportunity to consider your work for publication in The EMBO Journal. I look forward to your revision.

Best regards,

Ioannis

Ioannis Papaioannou, PhD
Editor, The EMBO Journal
i.papaioannou@embojournal.org

*****
Instructions for preparing your revised manuscript
*****

1. When you are ready to submit the revision, please upload:

- A Word file of the manuscript text (including legends of main Figures, EV Figures and Tables). Please make sure that changes are highlighted (or "tracked") to be clearly visible.

- Individual production-quality figure files (one file per figure). When assembling your figures, please refer to our figure preparation guidelines in order to ensure proper formatting and readability in print as well as on screen:
https://bit.ly/EMBOPressFigurePreparationGuideline
If the data shown in a figure are obtained from n {less than or equal to} 2, please use scatter plots showing the individual data

points.
Please also include scale bars in all microscopy images.
The following points must be specified in each figure legend:
i. the name of the statistical test used to generate error bars and P values
ii. the number (n) of independent experiments (please specify technical or biological replicates) underlying each data point (discussion of statistical methodology can be reported in the Materials and Methods section, but figure legends should contain a basic description of n, P, and the test applied)
iii. the nature of the bars and error bars (s.d., s.e.m.).
See also guidelines for figure legends: https://www.embopress.org/page/journal/14602075/authorguide#figureformat

- A point-by-point response to the referees' comments, with a detailed description of the changes made (as a word file). All referees' concerns must be fully addressed and their suggestions taken on board. When preparing your letter of response to the referees' comments, please bear in mind that this will form part of the Review Process File and will therefore be available online to the community. Please note that you have the possibility to opt out of the transparent process at any stage prior to publication by letting the editorial office know (contact@embojournal.org); if you do opt out, the Review Process File link will point to the following statement: "No Review Process File is available with this article, as the authors have chosen not to make the review process public in this case.". For more details on our Transparent Editorial Process, please visit our website: https://www.embopress.org/page/journal/14602075/authorguide#transparentprocess

- Expanded View (EV) files (replacing Supplementary Information) that are collapsible/expandable online. A maximum of 5 EV Figures can be typeset. EV Figures should be cited as "Figure EV1, Figure EV2" etc. in the text, and their respective legends should be included in the manuscript file after the legends of regular figures. See detailed instructions regarding Expanded View files here:
https://www.embopress.org/page/journal/14602075/authorguide#expandedview

- For the figures that you do NOT wish to display as Expanded View figures, they should be bundled together with their legends in a single PDF file called "Appendix", which should start with a short Table of Contents (including page numbers). Appendix figures should be referred to in the main text as: "Appendix Figure S1, Appendix Figure S2" etc. Please see detailed instructions here: https://www.embopress.org/page/journal/14602075/authorguide#expandedview

- A complete author checklist, which you can download from our author guidelines (https://www.embopress.org/page/journal/14602075/authorguide). Please note that the checklist will also be part of the Review Process File.

2. Please note that no statistics should be calculated and shown in Figures if n=2. Please also note that each p value should be reported as an exact value.

3. Before submitting your revision, primary datasets (and computer code, where appropriate) produced in this study need to be deposited in appropriate public databases (see https://www.embopress.org/page/journal/14602075/authorguide#dataavailability). In particular, all datasets such as DNA or RNA sequencing data, or mass spectrometry data generated in the study must be deposited in appropriate repositories. The accession numbers, database, and the specific URLs (links) should be listed in a formal "Data availability" section (placed after Methods), following the example below:

"The RNA-seq datasets produced in this study are available in the following database:
Gene Expression Omnibus GSE46843 (https://www.ncbi.nlm.nih.gov/geo/query/acc.cgi?acc=GSE46843)"

*** All links should resolve to a page where the data can be accessed. ***

*** Please remember to provide in the Data availability section of your revised manuscript reviewer passwords if the datasets are not yet public. ***

*** The Data Availability Section is restricted to new primary data that are part of this study. In case you have no data that require deposition in a public database, please state so instead of referring to the database: "Our study includes no data deposited in public repositories." under the heading "Data availability". ***

4. The materials and methods need to be described in the manuscript using our structured methods format, which is now required for all research articles. According to this format, the Methods section includes a single "Reagents and Tools Table" - listing key reagents, experimental models, software and relevant equipment including their sources and relevant identifiers- followed by a "Methods and Protocols" section describing the methods. Please download and fill our Reagents and Tools Table template (.docx), which you can find in our author guide:
https://www.embopress.org/page/journal/14602075/authorguide#structuredmethods. When submitting your revised manuscript, please do not include the Reagents and Tools Table in the Methods section of the manuscript but instead upload it as a separate file choosing the file type "Reagent Table".

5. Please check that the title and the abstract of the manuscript are brief, yet explicit, even to non-specialists. The length of the title should not exceed 100 characters, and the abstract should be a single paragraph not exceeding 175 words.

6. Please also note our reference format: https://www.embopress.org/page/journal/14602075/authorguide#referencesformat.

7. At EMBO Press we ask authors to provide source data for the main manuscript figures. You will receive a separate email with instructions for providing source data with your revised manuscript, including how to upload and organize the files.

8. Please remember: digital image enhancement is acceptable practice, as long as it accurately represents the original data and conforms to community standards. If a figure has been subjected to significant electronic manipulation, this must be noted in the figure legend or in the "Materials and Methods" section. The editors reserve the right to request original versions of figures and the original images that were used to assemble the figure.

9. Our journal encourages inclusion of data citations in the reference list to directly cite datasets that were obtained from public databases. Data citations in the article text are distinct from normal bibliographical citations and should directly link to the database records from which the data can be accessed. In the main text, data citations are formatted as follows: "Data ref: Smith et al, 2001" or "Data ref: NCBI Sequence Read Archive PRJNA342805, 2017". In the Reference list, data citations must be labeled with "[DATASET]". A data reference must provide the database name, accession number/identifiers, and a resolvable link to the landing page from which the data can be accessed at the end of the reference. Further instructions are available at: https://www.embopress.org/page/journal/14602075/authorguide#referencesformat.

10. We request authors to consider both actual and perceived competing interests. Please review our policy (https://www.embopress.org/page/journal/14602075/authorguide#conflictsofinterest) and update your competing interests statement if necessary. Please name this section 'Disclosure and competing interests statement' and place it after the Acknowledgements section.

11. Please note that all corresponding authors are required to provide an ORCID ID upon submission of a revised manuscript (https://orcid.org/). Please find instructions on how to link your ORCID ID to your account in our manuscript tracking system in our Author guidelines
(https://www.embopress.org/page/journal/14602075/authorguide#authorshipguidelines).

12. We use CRediT to specify the contributions of each author in the journal submission system. CRediT replaces the author contribution section, which should be removed from the manuscript. Please use the free text box to provide more detailed descriptions. See also guide to authors:
https://www.embopress.org/page/journal/14602075/authorguide#authorshipguidelines.

13. Further information is available in our Guide For Authors: https://www.embopress.org/page/journal/14602075/authorguide

14. We would also welcome the submission of cover suggestions or motifs to be used by our Graphics Illustrator in designing a cover.

15. Please use the link below to submit your revision:
https://emboj.msubmit.net/cgi-bin/main.plex

Referee #1:

The authors have used a fully defined in vitro reconstitution assay to assess the peptide editor, the molecular chaperone TAP-binding protein related, TAPBPR interaction with the glucosyl transferase UGGT, the lectin chaperone calreticulin, and Tapasin-erp57. The objective was to deduce the mechanism for MHC I transfer between TABPR and the peptide loading complex. The authors indicate in supplementary Figure EV1, the purification of the constituents of the reconstitution assay using size exclusion chromatography of secreted expressed constructs. The authors tested by size exclusion chromatography the effect of the peptide ETVSKQSNV on monoglucosylated β2m-HLA-A*68:02/TAPBPR complex in Figure EV2 and the glucosylation by Chaetomium thermophilum UGGT on MHC I/TAPBPR complex in Figure EV3. The effect of the peptide ETVSKQSNV on monoglucosylated β2m-HLA-A*68:02/TAPBPR complex and calreticulin is indicated in Figure EV4 as assessed by mass spectrometry.
Mass spectrometry of UGGT/UDP-glucose-treated single-chain β2m-HLA-A*68:02 is indicated in Figure 1B and size exclusion chromatography of monoglucosyltaed β2m-HLA-A*68:02
With tapasin-ERp57/PaSta1, TAPBPR, and calreticulin in Figures 1C, 1E. The PaSta1 Fab specific to tapasin was expressed in

HEK293 cells and isolated by Ni2+-NTA agarose resin followed by elution and size exclusion chromatography. The tapasin-Erp57 disulfide linked was isolated after expression in Sf21 insect cells and Ni2+-N-nitrilotriacetic acid (NTA) agarose resin affinity, elution and size exclusion chromatography.

Co-migration of calreticulin with glucosylated MHC I/tapasin-ERp57/PaSta1 by size exclusion chromatography is indicated in Figure 2A, 2B with mass spectrometry indicating the glucosylated MHC I in the complex in Figure 2 F, Table EV1.

Size exclusion chromatography, glycan mass spectrometry, and ultrasoft laser-induced liquid bead ion desorption mass spectrometry indicated the association of calreticulin with glucosylated MHC I from MHC I/TAPBPR in Figure 3. Ultrasoft laser-induced liquid bead ion desorption mass spectrometry assessed the effect of the peptide ETVSKQSNV on in Figure EV4 on glucosylated MHC I /calreticulin and MHC I/TAPBPR. With mutant calreticulin deleted for its C terminal acidic domain the authors tested by size exclusion chromatography the association with glucosylated β2m-HLA-A*68:02/TAPBPR in Figure 4. Marked effects of the mutant calreticulin were observed by size exclusion chromatography of incubations with tapasin-ERp57/PaSta1 and glucosylated, HLAA*68:02/TAPBPR in Figure 5.

The authors make at least 2 noteworthy conclusions. The first is the mechanism for calreticulin to release glucosylated MHC I from TAPBPR. The second is the requirement of the calreticulin C terminus to enable glucosylated MHC I to associate with tapasin.

These are genuine advances that are supported by the Figures and supplementary figures that include size exclusion chromatography with SDS PAGE and western blots with appropriate controls.
Have the mass spec raw data been deposited to a database such as PRIDE for access by the community?
Do the authors have any comments about the role of glucosidase II since reglucosylation is dependent on glucosidase II?

Referee #2:

Review of manuscript by Heinke et al.

This manuscript describes a reconstituted protein system tailored to answer questions about the role of glucosylation and calreticulin binding of MHC class I molecules. More specifically the authors want to address in how far glucosylation by the UGGT1 enzyme is required to allow the handover of non-optimally loaded MHCI molecules from TAPBPR to tapasin. Tapasin is a component of the peptide loading complex (PLC) and on the one hand chaperones MHCI molecules and on the other hand competes with low-affinity peptides in order to allow other peptides to more efficiently compete with the exchange factor. Still, non-optimally loaded pMHC complexes are thought to exit in the PLC and then undergo a second step of quality control by the Tapasin-related factor TAPBPR, which acts outside the PLC along the ER-Golgi exit route of the vesicular pathway.

The manuscript addresses an important scientific question by vigorous protein biochemical means and clearly shows that glycosylated MHCI in the TAPBPR complex can be competed out by Calreticulin and eventually give rise to the formation of a tapasin/MHCIglyc complex, thus corroborating a model of retrograde transfer of suboptimally loaded MHCI to the PLC. The essential components have been described before, the novelty of this manuscript lies in the biochemical dissection of a conceivable molecular hierarchy of events. While this manuscript thereby fits into the scopes of EMBO Journal, I have a few major concerns that should be addressed prior to publication.

1.) While the authors use terms such as "transfer" or "handover", there is no direct evidence from the reconstituted system itself. What is measured through the SEC approach shown here most likely reflects the relative affinities of the individual components for each other and its dependance on the terminal glucose moiety. However, in order to more clearly support such a back-exchange model the biochemical affinity constants of the components should be determined. What are the numbers here ? For example, it would be important to know the relative affinities of calreticulin versus TAPBPR for the glucosylated MHCI. Methods such as ITC or microscale thermophoresis could be used to determine the affinity constants quantitatively and to subsequently put these numbers in context of what is known about the cellular concentrations.
2.) The study is straightforward for the strongly TAPBPR-binding allotype A68*02. However, how do they envision quality control to be maintained for allotypes that do not bind to TAPBPR? In the discussion they suggest that one can distinguish between allotypes "which bind broad peptide repertoires but require more extensive QC, and "specialists", which stabilize quickly upon binding a narrow set of high-affinity peptides (Chappell et al, 2015)." This model is based on observations made for chicken MHC, but does it really hold true for the human (or mammalian) situation? Chicken MHC is polymorphic, but more or less monogenic, while the expression of several, conceivably competing MHCI might create different selection criteria in mammals. So, what is the evidence that for example a human "specialist" exists that is independent of TAPBPR? I would expect the immunopeptidome of such an allotype to be "narrower" than that of a TAPBPR-dependent generalist? Is there evidence out there that could be analyzed and scrutinize this important aspect?

3.) The recognition of UGGT1 and calreticulin regarding MHCI is distinct. While calreticulin recognizes stably folded peptide

MHC as well as empty MHC, UGGT1 senses the folding state of the MHC, despite both molecules interacting with the sugar moieties of N86 of the MHCI. How do they explain such difference ? While this goes beyond the scope of the study, I would expect a paragraph in the discussion that addresses this issue. And what would the role of TAPBPR be in this case ? Stabilizing the empty MHC or destabilizing it for UGGT1 recognition ?

Referee #3:

MHC class I molecules (MHC I) mature under quality control in the endoplasmic reticulum (ER) and play a crucial role in cellular immunity by presenting intracellular antigenic peptides on the cell surface. Newly synthesized MHC I molecules are folded with the assistance of ER chaperones and acquire suitable peptide. It has been established that MHC I forms the peptide loading complex (PLC) with ERp57, tapasin, and other components to facilitate peptide selection. However, the mechanism by which MHC I molecules that fail peptide selection are returned to the PLC for another round of peptide loading remains unclear. In particular, the molecular mechanisms underlying retrograde transport have not been fully elucidated.

In this study, the authors reconstitute the mechanism by which MHC I is transferred from TAPBPR to PLC. They demonstrated that UGGT1-mediated reglucosylation of MHC I promotes recognition by calreticulin (CRT), thereby facilitating the transfer of MHC I to the PLC. However, there are concerns regarding the novelty of these findings.

The manuscript states, "It is not known how MHC I molecules that fail TAPBPR- mediated peptide editing are returned to the PLC for another round of high-affinity peptide selection." However, the importance of UGGT-dependent reglucosylation and its role in PLC recruitment has been reported (Wearsch et al., PNAS, 2011; Zhang et al., PNAS, 2011). Therefore, although this study provides value by directly reconstituting the transfer mechanism, its novelty in demonstrating the requirement of glucose addition for PLC recruitment appears limited and may not meet the criteria of the EMBO Journal.

Minor points

• In Fig. 3, MHCglc is indicated in black, whereas it is shown in red in Fig. 4. The color scheme should be unified to avoid confusion for the readers.

• Fig. 1E and 5B appear to use the same experimental system; however, the elution fractions of the bands differ. In Fig. 1E, tsn-ERp57 elutes in fractions 3-5, whereas in Fig. 5B, it elutes in fractions 1-3. The authors should explain the reason for this discrepancy in the manuscript.

• In Fig. 4, the authors mentioned a disease-associated CRT frameshift mutant that reverses the charge of the C-terminal acidic helix. However, these experiments were performed using a mutant lacking the C-terminal acidic helix. This experimental design does not allow discrimination between the importance of the C-terminal structure itself and the role of the amino acid charge; therefore, it does not adequately address disease relevance. Validation using actual disease-associated mutants is desirable.

• In Fig. 1B, a peak at approximately 50 kDa is observed. The left panel shows only the M9 glycoform, whereas the right panel shows the G1M8 glycoform. However, molecular weight alone cannot distinguish glucose from mannose residues, making it unclear whether G1M8 truly increased in the right panel. Therefore, this labeling may be misleading. The same concern applies to Fig. 2F.

• Fig. 5B is not mentioned in the main text. On page 10, line 2, "Fig. 5A" should be revised to "Fig. 5A and 5B."

Point-to-Point Response to Reviewers – EMBOJ-2025-123404

Referee #1:

*The authors have used a fully defined in vitro reconstitution assay to assess the peptide editor, the molecular chaperone TAP-binding protein related, TAPBPR interaction with the glucosyl transferase UGGT, the lectin chaperone calreticulin, and Tapasin-ERp57. The objective was to deduce the mechanism for MHC I transfer between TABPR and the peptide loading complex. The authors indicate in Supplementary Figure EV1, the purification of the constituents of the reconstitution assay using size exclusion chromatography of secreted expressed constructs. The authors tested by size exclusion chromatography the effect of the peptide ETVSKQSNV on monoglucosylated β2m-HLA-A*68:02/TAPBPR complex in Figure EV2 and the glucosylation by Chaetomium thermophilum UGGT on MHC I/TAPBPR complex in Figure EV3. The effect of the peptide ETVSKQSNV on monoglucosylated β2m-HLA-A*68:02/TAPBPR complex and calreticulin is indicated in Figure EV4 as assessed by mass spectrometry.*

*Mass spectrometry of UGGT/UDP-glucose-treated single-chain β2m-HLA-A*68:02 is indicated in Figure 1B and size exclusion chromatography of monoglucosylated β2m-HLA-A*68:02 with tapasin-ERp57/PaSta1, TAPBPR, and calreticulin in Figures 1C, 1E. The PaSta1 Fab specific to tapasin was expressed in HEK293 cells and isolated by Ni^{2+}-NTA agarose resin followed by elution and size exclusion chromatography. The tapasin-ERp57 disulfide linked was isolated after expression in Sf21 insect cells and Ni2+-N-nitrilotriacetic acid (NTA) agarose resin affinity, elution and size exclusion chromatography. Co-migration of calreticulin with glucosylated MHC I/tapasin-ERp57/PaSta1 by size exclusion chromatography is indicated in Figure 2A, 2B with mass spectrometry indicating the glucosylated MHC I in the complex in Figure 2 F, Table EV1.*

*Size exclusion chromatography, glycan mass spectrometry, and ultrasoft laser-induced liquid bead ion desorption mass spectrometry indicated the association of calreticulin with glucosylated MHC I from MHC I/TAPBPR in Figure 3. Ultrasoft laser-induced liquid bead ion desorption mass spectrometry assessed the effect of the peptide ETVSKQSNV on in Figure EV4 on glucosylated MHC I/calreticulin and MHC I/TAPBPR. With mutant calreticulin deleted for its C terminal acidic domain the authors tested by size exclusion chromatography the association with glucosylated β2m-HLA-A*68:02/TAPBPR in Figure 4. Marked effects of the mutant calreticulin were observed by size exclusion chromatography of incubations with tapasin-ERp57/PaSta1 and glucosylated, HLAA*68:02/TAPBPR in Figure 5.*

The authors make at least 2 noteworthy conclusions. The first is the mechanism for calreticulin to release glucosylated MHC I from TAPBPR. The second is the requirement of the calreticulin C terminus to enable glucosylated MHC I to associate with tapasin.

These are genuine advances that are supported by the Figures and supplementary figures that include size exclusion chromatography with SDS PAGE and western blots with appropriate controls. Have the mass spec raw data been deposited to a database such as PRIDE for access by the community?

Reply: We thank the reviewer for this thorough and highly positive assessment of our work. We appreciate the careful summary of the experimental strategy and key findings, as well as the recognition that the study provides genuine mechanistic advances regarding (i) the role of calreticulin in the release of monoglucosylated MHC I from TAPBPR and (ii) the functional significance of the calreticulin C-terminal acidic domain in enabling transfer to tapasin-containing complexes. We are also grateful for the reviewer's acknowledgement that the biochemical conclusions are well supported by

the extensive SEC, SDS-PAGE, immunoblotting, and mass spectrometry analyses, as well as by the inclusion of appropriate controls throughout the study.

In response to the reviewer's question regarding data availability, we agree that deposition of the raw MS data is important to ensure transparency and accessibility for the scientific community. We have therefore deposited all relevant raw and processed MS datasets underlying Figures 1–3 and Table EV1 in the GUDe repository under the accession number (gude.18f9-c6gc); they are publicly accessible at https://doi.org/10.25716/gude.18f9-c6gc.

<u>Changes to the manuscript:</u> A statement on data deposition has been added to the Methods section ("Mass spectrometry").

We thank the reviewer once again for the positive evaluation of the study and for highlighting the significance of the mechanistic insights provided by our reconstitution system.

Do the authors have any comments about the role of glucosidase II since reglucosylation is dependent on glucosidase II?

<u>Reply:</u> We thank the reviewer for highlighting the role of glucosidase II in ER glycoprotein quality control. We concur that glucosidase II is an integral component of the calnexin/calreticulin cycle, as its sequential glucose trimming generates the deglucosylated glycoforms that serve as substrates for UGGT1-mediated reglucosylation. In our reconstituted system, we specifically isolated the reglucosylation-dependent recruitment step by starting from defined deglucosylated MHC I glycoforms and reconstituting UGGT1-mediated monoglucosylation *in vitro*. This experimental design allowed us to directly examine how reglucosylation promotes calreticulin-dependent re-engagement of MHC I with the peptide-loading complex. In the revised version, we have now clarified the relationship between glucosidase II and UGGT1 in the introduction part.

<u>Changes to the manuscript</u> <u>(Introduction, page 2):</u> "Glucosidase II plays a central role in this glycan cycle by sequentially removing the two innermost glucose residues. This process generates initially monoglucosylated and subsequently fully deglucosylated glycoforms, which mediate entry into the calnexin/calreticulin (Cnx/Crt) cycle as well as release from it (Deprez *et al*, 2005; Helenius & Aebi, 2004). Another key regulator of this process is UDP-glucose:glycoprotein glucosyltransferase 1 (UGGT1), which selectively reglucosylates non-native glycoproteins, thereby prolonging their residence time in the Cnx/Crt cycle until proper folding is achieved (Kozlov & Gehring, 2020; Ninagawa *et al*, 2024). Through this iterative trimming process, glucosidase II functionally opposes UGGT1-mediated reglucosylation and enables repeated rounds of chaperone binding, thereby serving as another driver of glycoprotein QC."

Referee #2:

This manuscript describes a reconstituted protein system tailored to answer questions about the role of glucosylation and calreticulin binding of MHC class I molecules. More specifically the authors want to address in how far glucosylation by the UGGT1 enzyme is required to allow the handover of non-optimally loaded MHCI molecules from TAPBPR to tapasin. Tapasin is a component of the peptide loading complex (PLC) and on the one hand chaperones MHC I molecules and on the other hand

competes with low-affinity peptides in order to allow other peptides to more efficiently compete with the exchange factor. Still, non-optimally loaded pMHC complexes are thought to exit in the PLC and then undergo a second step of quality control by the Tapasin-related factor TAPBPR, which acts outside the PLC along the ER-Golgi exit route of the vesicular pathway.

The manuscript addresses an important scientific question by vigorous protein biochemical means and clearly shows that glycosylated MHC I in the TAPBPR complex can be competed out by Calreticulin and eventually give rise to the formation of a tapasin/MHC I^{glyc} complex, thus corroborating a model of retrograde transfer of suboptimally loaded MHCI to the PLC. The essential components have been described before; the novelty of this manuscript lies in the biochemical dissection of a conceivable molecular hierarchy of events. While this manuscript thereby fits into the scopes of EMBO Journal, I have a few major concerns that should be addressed prior to publication.

Reply: We thank the reviewer for the careful evaluation of our manuscript and for recognizing both the importance of the scientific question and the strength of our biochemical reconstitution approach. We are particularly encouraged by the reviewer's assessment that our data clearly demonstrate the formation of a calreticulin-mediated signaling pathway that enables the transfer of glycosylated MHC I molecules from TAPBPR to tapasin, thereby supporting a model of retrograde quality control and reintegration into the PLC. We also appreciate the reviewer's recognition that the novelty of the study lies in the biochemical dissection of the molecular hierarchy underlying these events.

We are pleased that the reviewer considers the work suitable for the scope of EMBO Journal. Below, we address each of the reviewer's key comments in detail. In response, we have performed additional analyses and extensively revised the manuscript to clarify the experimental rationale, strengthen the mechanistic interpretation, and improve the presentation of the data. We believe that these revisions substantially strengthen the manuscript and fully address the reviewer's concerns.

1) While the authors use terms such as "transfer" or "handover", there is no direct evidence from the reconstituted system itself. What is measured through the SEC approach shown here most likely reflects the relative affinities of the individual components for each other and its dependance on the terminal glucose moiety. However, in order to more clearly support such a back-exchange model, the biochemical affinity constants of the components should be determined. What are the numbers here? For example, it would be important to know the relative affinities of calreticulin versus TAPBPR for the glucosylated MHCI. Methods such as ITC or microscale thermophoresis could be used to determine the affinity constants quantitatively and to subsequently put these numbers in context of what is known about the cellular concentrations.

Reply: We thank the reviewer for this important observation and agree that our SEC-based experiments alone do not establish a strictly directional "handover" mechanism. Rather, they report on the redistribution of MHC I between competing chaperone environments under defined biochemical conditions. To address this point quantitatively, we performed isothermal titration calorimetry (ITC) experiments for key interactions underlying this exchange process.

Our measurements show that calreticulin (Crt) binds monoglucosylated MHC I in the context of the MHC I/TAPBPR complex with low micromolar affinity (K_d = 0.7 ± 0.3 µM), consistent with a strong glycan-dependent interaction. In contrast, no interaction was observed for the non-glucosylated MHC I/TAPBPR complex. We further determined that Crt interacts with the tapasin-ERp57 complex

with similarly low micromolar affinity (K_d = 3.0 ± 2.6 µM). These measurements support a model in which Crt can simultaneously engage reglucosylated MHC I and the PLC environment with physiologically relevant affinity.

Notably, the affinity of the full-length Crt for tapasin-ERp57 is stronger than previously reported for the interaction of the isolated Crt P-domain with ERp57 alone (Frickel *et al*, 2002), suggesting additional stabilizing contacts within the PLC, likely involving the acidic C-terminal region of Crt, as indicated by our structural work (Blees *et al*, 2017; Domnick *et al*, 2022).

Given the high abundance of Crt within the ER lumen relative to TAPBPR (Bakunts *et al*, 2017; Lievremont *et al*, 1997), these affinity relationships are expected to favor Crt-mediated recruitment of reglucosylated MHC I into the calreticulin/tapasin pathway. We therefore revised the manuscript to describe the process more precisely as an affinity-driven redistribution mechanism rather than a strictly directional transfer reaction.

Changes to the manuscript: We have included several new sets of ITC experiments in the Results section (page 9 and 12) and incorporated the following statement into the Discussion: "In this context, it is of note that calreticulin is a highly abundant ER-resident chaperone that is present in concentrations ranging from tens to hundreds of micromolar, whereas TAPBPR is expressed at substantially lower levels. Considering the low-micromolar affinities measured here, these concentration differences establish conditions that strongly favor Crt-mediated engagement of reglucosylated MHC I and recruitment into the peptide-loading complex environment. Future studies will be required to determine to what extent compartmentalization further contributes to the selection and stabilization of high-affinity peptide–MHC I complexes."

*2) The study is straightforward for the strongly TAPBPR-binding allotype A68*02. However, how do they envision quality control to be maintained for allotypes that do not bind to TAPBPR? In the discussion they suggest that one can distinguish between allotypes "which bind broad peptide repertoires but require more extensive QC, and "specialists", which stabilize quickly upon binding a narrow set of high-affinity peptides (Chappell et al, 2015)." This model is based on observations made for chicken MHC, but does it really hold true for the human (or mammalian) situation? Chicken MHC is polymorphic, but more or less monogenic, while the expression of several, conceivably competing MHC I might create different selection criteria in mammals. So, what is the evidence that for example a human "specialist" exists that is independent of TAPBPR? I would expect the immunopeptidome of such an allotype to be "narrower" than that of a TAPBPR-dependent generalist? Is there evidence out there that could be analyzed and scrutinize this important aspect?*

Reply: We thank the reviewer for this important and thoughtful comment. We agree that the "generalist/specialist" framework proposed by *Chappell et al.* was derived from the chicken MHC system (Chappell *et al*, 2015) and therefore cannot be directly extrapolated to mammalian MHC I biology. In particular, as the reviewer correctly states, the co-expression of multiple class I allotypes in mammals likely imposes additional layers of selection and competition that are absent or reduced in the largely monogenic chicken MHC.

By citing this framework, we did not intend to imply that a direct mammalian equivalent has been conclusively established, but rather to provide a conceptual model illustrating how distinct MHC I allotypes may differ in their reliance on quality-control factors such as tapasin, TAPBPR, UGGT1, and

calreticulin. We acknowledge that direct evidence for truly TAPBPR-independent “specialist” allotypes in humans remains limited.

Nevertheless, several studies support the notion that human MHC I allotypes differ substantially in their reliance on peptide-loading cofactors such as tapasin and TAPBPR (Bashirova *et al*, 2020; Ilca *et al*, 2019). In particular, some allotypes exhibit relative independence from editing factors and maintain efficient peptide loading and surface expression without prolonged PLC engagement, whereas others strongly depend on chaperone-assisted editing. These observations suggest that human MHC I molecules occupy a continuum of peptide-loading strategies, even though a strict ‘generalist/specialist’ classification analogous to the chicken system remains unproven. We have therefore revised the Discussion to tone down this point and explicitly state that the applicability of the “generalist/specialist” concept to mammalian MHC I biology remains hypothetical and requires further experimental validation.

Changes to the manuscript: We modified the relevant paragraph in the Discussion (page 15) as follows: “Direct evidence for TAPBPR-independent ‘specialist’ allotypes in the human system remains limited. It therefore remains unclear how broadly the mechanism described here applies across the MHC I repertoire. Although allotype-specific differences in tapasin and TAPBPR dependence are well documented in humans, it is unresolved whether these differences map onto the ‘generalist/specialist’ dichotomy originally proposed for the chicken MHC system.”

We believe that this revision better reflects the current state of research and avoids conclusions beyond the available evidence.

3) The recognition of UGGT1 and calreticulin regarding MHCI is distinct. While calreticulin recognizes stably folded peptide MHC as well as empty MHC, UGGT1 senses the folding state of the MHC, despite both molecules interacting with the sugar moieties of N86 of the MHCI. How do they explain such difference? While this goes beyond the scope of the study, I would expect a paragraph in the discussion that addresses this issue. And what would the role of TAPBPR be in this case? Stabilizing the empty MHC or destabilizing it for UGGT1 recognition?

Reply: We thank the reviewer for this insightful comment. We agree that there is an important distinction between the recognition of MHC I by calreticulin and UGGT1, even though both factors bind to the N86-linked glycan. In fact, calreticulin primarily recognizes the glycan state itself, whereas UGGT1 acts as a folding sensor that integrates information from both the glycan and the conformational state of the protein substrate. We are aware that this point deserves additional discussion in the manuscript.

Current models suggest that UGGT1 links glycan recognition with the detection of conformational features associated with incompletely folded glycoproteins, including increased structural flexibility and transient exposure of hydrophobic surfaces. In the context of MHC I, peptide-deficient or suboptimally loaded conformers are thought to transiently sample partially destabilized states that become substrates for UGGT1-mediated reglucosylation (Pearse *et al*, 2010; Sagert *et al*, 2023; Zhang *et al*, 2011). In contrast, calreticulin recognition is largely dictated by the presence of the terminal glucose on the A-branch of the glycan and therefore does not directly discriminate between peptide-loaded and peptide-receptive MHC I conformers, provided the monoglucosylated glycan is accessible.

This distinction is consistent with our observation that calreticulin can engage monoglucosylated MHC I regardless of high-affinity peptide loading, whereas UGGT1-mediated reglucosylation requires access to conformationally non-native or dynamic MHC I states. Thus, UGGT1 functions upstream as a conformational checkpoint that selectively reglucosylates incompletely matured MHC I molecules, while calreticulin functions downstream as a glycan-dependent capture factor that retrieves reglucosylated substrates into the calnexin/calreticulin cycle and ultimately back to the PLC.

Regarding the role of TAPBPR, we favor a model in which TAPBPR primarily stabilizes a peptide-receptive yet conformationally dynamic state of MHC I that remains competent for UGGT1 recognition. Structural and biochemical studies indicate that TAPBPR widens the peptide-binding groove and promotes conformational plasticity of peptide-deficient MHC I molecules rather than fully destabilizing them (Ilca *et al*, 2018; Sagert *et al*, 2023; Thomas & Tampé, 2017). In this framework, TAPBPR would maintain MHC I in a long-lived editing-competent intermediate state that exposes the structural features sensed by UGGT1, thereby facilitating reglucosylation and re-entry into the calreticulin-dependent quality-control cycle.

<u>Changes to the manuscript:</u> We added the following paragraph to the Discussion (page 12): "A clear conceptual distinction emerges between UGGT1- and Crt-mediated recognition of MHC I. Calreticulin primarily recognizes the monoglucosylated glycan and can engage both peptide-loaded and peptide-receptive MHC I. By contrast, UGGT1 acts as a folding sensor that selectively reglucosylates conformationally dynamic or incompletely matured glycoproteins. In the case of MHC I, peptide-deficient or suboptimally loaded conformers likely expose structural features recognized by UGGT1, whereas calreticulin engagement occurs downstream once the monoglucosylated glycan has been installed. In this framework, TAPBPR may stabilize a peptide-receptive yet conformationally flexible MHC I state that remains competent for UGGT1 recognition and reglucosylation, thereby promoting re-entry into the calreticulin-dependent quality-control cycle."

We believe that inclusion of this discussion strengthens the conceptual framework of the manuscript and clarifies the distinct yet coordinated roles of UGGT1, calreticulin, and TAPBPR in MHC I quality control.

Referee #3:

MHC class I molecules (MHC I) mature under quality control in the endoplasmic reticulum (ER) and play a crucial role in cellular immunity by presenting intracellular antigenic peptides on the cell surface. Newly synthesized MHC I molecules are folded with the assistance of ER chaperones and acquire suitable peptide. It has been established that MHC I forms the peptide loading complex (PLC) with ERp57, tapasin, and other components to facilitate peptide selection. However, the mechanism by which MHC I molecules that fail peptide selection are returned to the PLC for another round of peptide loading remains unclear. In particular, the molecular mechanisms underlying retrograde transport have not been fully elucidated.

In this study, the authors reconstitute the mechanism by which MHC I is transferred from TAPBPR to PLC. They demonstrated that UGGT1-mediated reglucosylation of MHC I promotes recognition by calreticulin (CRT), thereby facilitating the transfer of MHC I to the PLC. However, there are concerns regarding the novelty of these findings.

The manuscript states, "It is not known how MHC I molecules that fail TAPBPR-mediated peptide editing are returned to the PLC for another round of high-affinity peptide selection." However, the importance of UGGT-dependent reglucosylation and its role in PLC recruitment has been reported (Wearsch et al., PNAS, 2011; Zhang et al., PNAS, 2011). Therefore, although this study provides value by directly reconstituting the transfer mechanism, its novelty in demonstrating the requirement of glucose addition for PLC recruitment appears limited and may not meet the criteria of the EMBO Journal.

Reply: We thank the reviewer for this important comment and agree that the role of UGGT1-mediated reglucosylation in MHC I quality control and PLC recruitment has been established previously by the important studies from the Cresswell laboratory (Wearsch et al., 2011; Zhang et al., 2011). It was never our intention to claim the discovery of the reglucosylation-dependent retrieval pathway as our own.

Rather, we view the key advancement of this study as the direct biochemical reconstitution and mechanistic dissection of the glycan-driven MHC I recycling process using purified components. Specifically, our system allowed us to define a sequential pathway in which UGGT1-mediated reglucosylation promotes calreticulin engagement of TAPBPR-bound MHC I and enables its subsequent recruitment into the tapasin–ERp57-containing PLC environment. In addition, using the reconstituted system enabled us to identify a functional contribution of the calreticulin acidic C-terminal region to PLC association and to quantitatively characterize key interaction affinities underlying the exchange process.

We recognize that the original wording in the manuscript overstated the novelty of the reglucosylation requirement itself. We therefore revised the Introduction and Discussion to more clearly distinguish prior cellular observations from the mechanistic insights gained here through biochemical reconstitution.

Minor points

1) *In Fig. 3, MHC I^{glc} is indicated in black, whereas it is shown in red in Fig. 4. The color scheme should be unified to avoid confusion for the readers.*

   Reply: We thank the reviewer for pointing this out. The color scheme for monoglucosylated MHC I has now been unified throughout all figures to avoid confusion.

2) *Fig. 1E and 5B appear to use the same experimental system; however, the elution fractions of the bands differ. In Fig. 1E, tsn-ERp57 elutes in fractions 3-5, whereas in Fig. 5B, it elutes in fractions 1-3. The authors should explain the reason for this discrepancy in the manuscript.*

   Reply: We thank the reviewer for this comment. The tapasin-ERp57 complex elutes at the same retention volume in both sets of experiments, as shown in the corresponding chromatograms. The apparent discrepancy arises because different fraction ranges were collected and analyzed in the respective experiments to accommodate the distinct sample compositions. We have clarified this in the figure legend.

3) *In Fig. 4, the authors mentioned a disease-associated CRT frameshift mutant that reverses the charge of the C-terminal acidic helix. However, these experiments were performed using a mutant lacking the C-terminal acidic helix. This experimental design does not allow discrimination between the importance of the C-terminal structure itself and the role of the amino acid charge; therefore, it does not adequately address disease relevance. Validation using actual disease-associated mutants is desirable.*

Reply: We thank the reviewer for pointing this out. We acknowledge that the Crt$^{\Delta acidic}$ construct used in our study does not fully recapitulate disease-associated Crt frameshift mutants found in myeloproliferative neoplasms, in which the acidic C-terminal region is not completely removed but is replaced by a positively charged sequence. Consequently, our experiments do not distinguish between the contributions of acidic helix architecture, charge reversal, and altered sequence composition.

Our intention was not to directly model the disease-associated variants, but rather to determine whether the acidic C-terminal region of calreticulin functionally contributes to recruitment of MHC I into the tapasin-ERp57-containing PLC environment. In this context, the deletion construct serves as a reductionist mechanistic tool.

We agree, however, that the original wording overstated the direct relevance of the experiment. We therefore revised the manuscript to clarify that our findings are consistent with the phenotypes previously reported for disease-associated calreticulin mutants.

We also attempted to produce the disease-associated Crt^{del52} variant for analysis in our reconstituted system. However, despite extensive optimization in both the bacterial and mammalian expression systems, we were unable to obtain monodisperse full-length protein suitable for biochemical reconstitution because the mutant underwent extensive truncation and aggregation (**Fig. R1** and **R2**).

Changes to the manuscript: We revised the relevant Discussion section (page 12) to read: "Although our Crt$^{\Delta acidic}$ construct does not directly mimic disease-associated calreticulin frameshift mutants, the observed defect in tapasin-ERp57 recruitment is consistent with previous cellular studies reporting impaired PLC incorporation and defective MHC I maturation for these variants (Arshad & Cresswell, 2018). The inability of these mutants to support PLC function provides a mechanistic explanation for reduced surface MHC I levels observed in pathological settings. Future work using authentic disease-associated mutants will be required to disentangle the contributions of charge reversal, altered sequence composition, and loss of ER retention to these phenotypes."

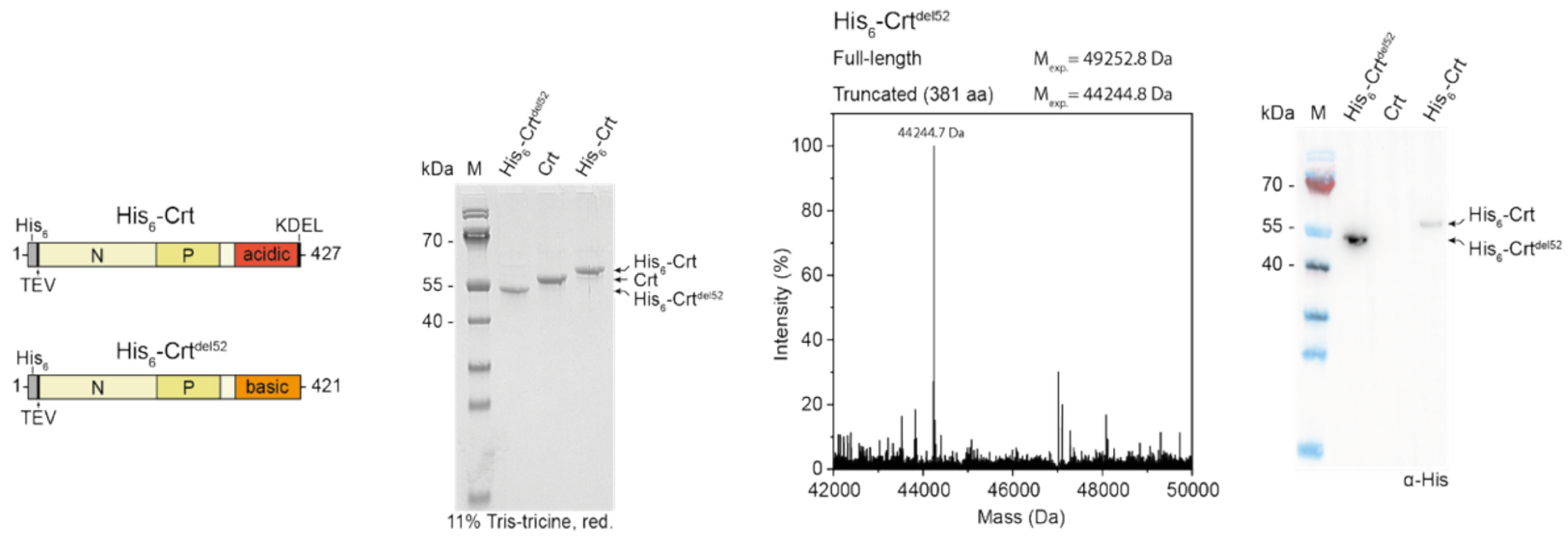

Fig. R1. Attempts to purify recombinant Crt frame-shift mutant Crt^{del52} from bacterial BL21 GOLD expression. A bar diagram of the expression constructs of Crt and Crt^{del52} (upper left) provide context to the SDS-PAGE and immunoblot against the His-tag of purified Crt variants (right). LC-MS data on the purified Crt^{del52} revealed a C-terminal truncation of 40 amino acids (381 aa are the remaining amino acids from the protein sequence). N – lectin domain, P – P-arm, acidic – acidic helix, basic – basic helix, TEV – TEV cleavage site.

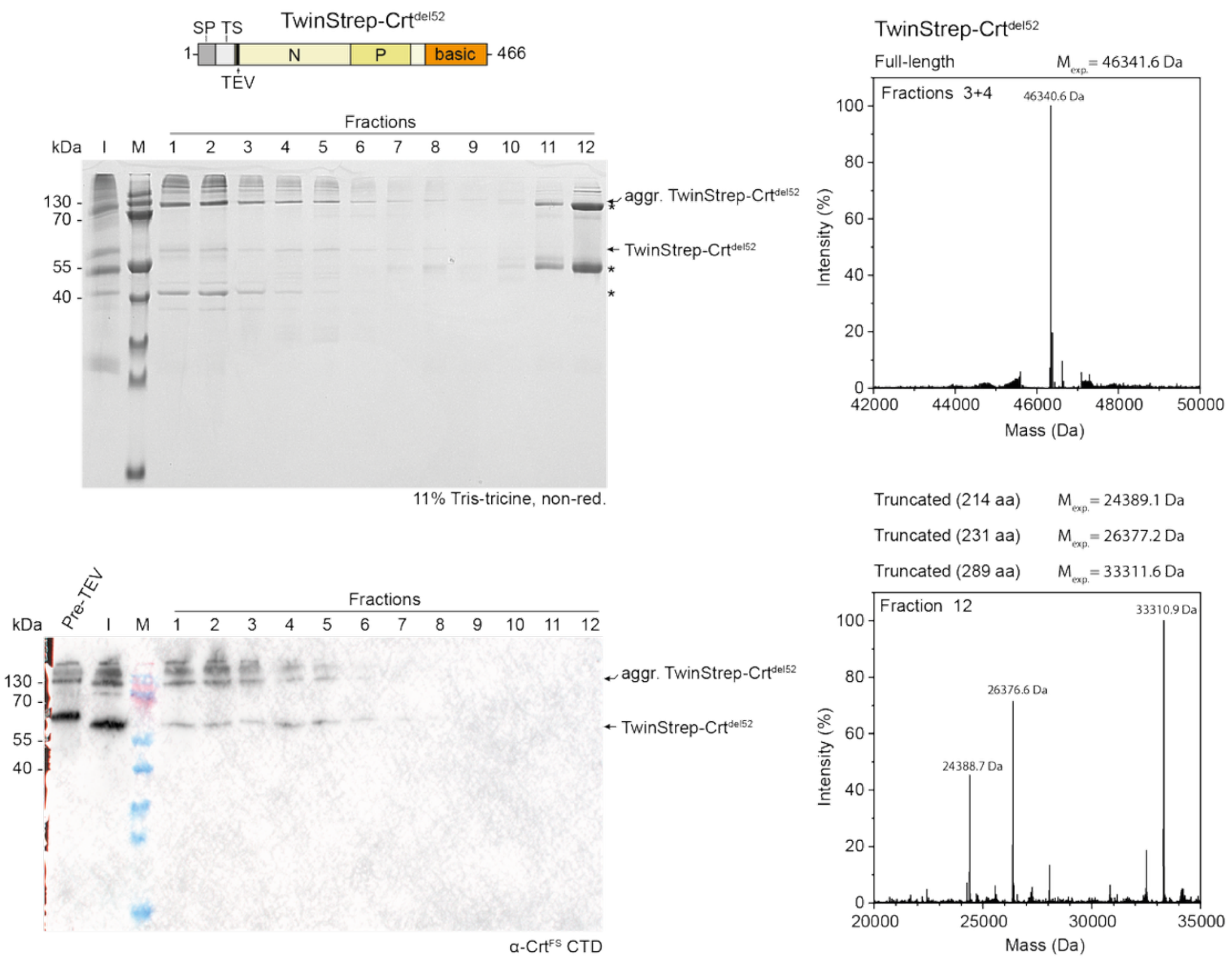

Fig. R2. Attempts to purify secreted, recombinant Crt frame-shift mutant Crt^{del52} from human HEK293-F cell expression. Bar diagram of the expression construct of TwinStrep-Crt^{del52} (upper left). SDS-PAGE and immunoblot against the C-terminal domain of Crt^{FS} mutants (lower left) indicate a mixture of different Crt^{del52} truncations. LC-MS data on gel-filtrated Crt^{del52} revealed the full-length protein and C-terminal truncations (right panels). Notably, all Crt variants run as aggregated proteins during size exclusion chromatography. N – lectin domain, P – P-arm, basic – basic helix, TEV – TEV cleavage site, TS – TwinStrep-tag, aggr. - aggregated.

4) *In Fig. 1B, a peak at approximately 50 kDa is observed. The left panel shows only the M9 glycoform, whereas the right panel shows the G1M8 glycoform. However, molecular weight alone cannot distinguish glucose from mannose residues, making it unclear whether G1M8 truly increased in the right panel. Therefore, this labeling may be misleading. The same concern applies to Fig. 2F.*

Reply: We thank the reviewer for highlighting this important limitation. We agree that intact molecular mass alone cannot distinguish between glucose- and mannose-containing glycoforms with identical monosaccharide composition, such as G1M8 and M9.

Our assignments were therefore based on comparative LC-MS analysis before and after UGGT-mediated reglucosylation together with the known enzymatic reaction context, rather than

molecular weight measurements alone. Nevertheless, we agree that the original labeling may have implied a level of structural assignment not directly supported by intact mass measurements.

To avoid overinterpretation, we specifically noted how assignments were done in the methods sectionto clarify that the indicated glycoforms represent the predominant species inferred from the UGGT-dependent shift rather than direct discrimination between glucose- versus mannose-containing structures by mass alone.

5) *Fig. 5B is not mentioned in the main text. On page 10, line 2, "Fig. 5A" should be revised to "Fig. 5A and 5B."*

Reply: We thank the reviewer for detecting this omission. The respective sentence in the Results section has been corrected to refer to both panels ("Fig. 5A,B").

References:

Arshad N, Cresswell P (2018) Tumor-associated calreticulin variants functionally compromise the peptide loading complex and impair its recruitment of MHC-I. *J Biol Chem* 293: 9555–9569. doi:10.1074/jbc.RA118.002836

Bakunts A, Orsi A, Vitale M, Cattaneo A, Lari F, Tade L, Sitia R, Raimondi A, Bachi A, van Anken E (2017) Ratiometric sensing of BiP-client versus BiP levels by the unfolded protein response determines its signaling amplitude. *eLife* 6 doi:10.7554/eLife.27518

Bashirova AA, Viard M, Naranbhai V, Grifoni A, Garcia-Beltran W, Akdag M, Yuki Y, Gao X, O'HUigin C, Raghavan M, Wolinsky S, Bream JH, Duggal P, Martinson J, Michael NL, Kirk GD, Buchbinder SP, Haas D, Goedert JJ, Deeks SG *et al* (2020) HLA tapasin independence: broader peptide repertoire and HIV control. *Proc Natl Acad Sci U S A* 117: 28232–28238. doi:10.1073/pnas.2013554117

Blees A, Januliene D, Hofmann T, Koller N, Schmidt C, Trowitzsch S, Moeller A, Tampé R (2017) Structure of the human MHC-I peptide-loading complex. *Nature* 551: 525–528. doi:10.1038/nature24627

Chappell P, Meziane el K, Harrison M, Magiera L, Hermann C, Mears L, Wrobel AG, Durant C, Nielsen LL, Buus S, Ternette N, Mwangi W, Butter C, Nair V, Ahyee T, Duggleby R, Madrigal A, Roversi P, Lea SM, Kaufman J (2015) Expression levels of MHC class I molecules are inversely correlated with promiscuity of peptide binding. *eLife* 4: e05345. doi:10.7554/eLife.05345

Deprez P, Gautschi M, Helenius A (2005) More than one glycan is needed for ER glucosidase II to allow entry of glycoproteins into the calnexin/calreticulin cycle. *Mol Cell* 19: 183–195. doi:10.1016/j.molcel.2005.05.029

Domnick A, Winter C, Sušac L, Hennecke L, Hensen M, Zitzmann N, Trowitzsch S, Thomas C, Tampé R (2022) Molecular basis of MHC I quality control in the peptide loading complex. *Nat Commun* 13: 4701. doi:10.1038/s41467-022-32384-z

Frickel EM, Riek R, Jelesarov I, Helenius A, Wuthrich K, Ellgaard L (2002) TROSY-NMR reveals interaction between ERp57 and the tip of the calreticulin P-domain. *Proc Natl Acad Sci U S A* 99: 1954–1959. doi:10.1073/pnas.042699099

Helenius A, Aebi M (2004) Roles of N-linked glycans in the endoplasmic reticulum. *Annu Rev Biochem* 73: 1019–1049. doi:10.1146/annurev.biochem.73.011303.073752

Ilca FT, Drexhage LZ, Brewin G, Peacock S, Boyle LH (2019) Distinct polymorphisms in HLA class I molecules govern their susceptibility to peptide editing by TAPBPR. *Cell Rep* 29: 1621–1632 e1623. doi:10.1016/j.celrep.2019.09.074

Ilca FT, Neerincx A, Hermann C, Marcu A, Stevanovic S, Deane JE, Boyle LH (2018) TAPBPR mediates peptide dissociation from MHC class I using a leucine lever. *eLife* 7 doi:10.7554/eLife.40126

Kozlov G, Gehring K (2020) Calnexin cycle - structural features of the ER chaperone system. *FEBS J* 287: 4322–4340. doi:10.1111/febs.15330

Lievremont JP, Rizzuto R, Hendershot L, Meldolesi J (1997) BiP, a major chaperone protein of the endoplasmic reticulum lumen, plays a direct and important role in the storage of the rapidly exchanging pool of Ca^{2+}. *J Biol Chem* 272: 30873–30879. doi:10.1074/jbc.272.49.30873

Ninagawa S, Matsuo M, Ying D, Oshita S, Aso S, Matsushita K, Taniguchi M, Fueki A, Yamashiro M, Sugasawa K, Saito S, Imami K, Kizuka Y, Sakuma T, Yamamoto T, Yagi H, Kato K, Mori K (2024) UGGT1-mediated reglucosylation of N-glycan competes with ER-associated degradation of unstable and misfolded glycoproteins. *eLife* doi:10.7554/eLife.93117.2

Pearse BR, Tamura T, Sunryd JC, Grabowski GA, Kaufman RJ, Hebert DN (2010) The role of UDP-Glc:glycoprotein glucosyltransferase 1 in the maturation of an obligate substrate prosaposin. *J Cell Biol* 189: 829–841. doi:10.1083/jcb.200912105

Sagert L, Winter C, Ruppert I, Zehetmaier M, Thomas C, Tampé R (2023) The ER folding sensor UGGT1 acts on TAPBPR-chaperoned peptide-free MHC I. *eLife* 12 doi:10.7554/eLife.85432

Thomas C, Tampé R (2017) Proofreading of peptide-MHC complexes through dynamic multivalent interactions. *Front Immunol* 8: 65. doi:10.3389/fimmu.2017.00065

Zhang W, Wearsch PA, Zhu Y, Leonhardt RM, Cresswell P (2011) A role for UDP-glucose glycoprotein glucosyltransferase in expression and quality control of MHC class I molecules. *Proc Natl Acad Sci U S A* 108: 4956–4961. doi:10.1073/pnas.1102527108

Dear Prof. Tampé,

Thanks again for the submission of your revised manuscript (EMBOJ-2025-123404R) to The EMBO Journal for our consideration, and for your patience during re-review. Your manuscript and your point-by-point responses to the comments of all original referees have now been seen by referees #2 and #3, and we have received their comments, which are appended below.

As you will see, both referees are satisified with the revision and find the initially raised points and concerns adequately addressed. In light of this input and their recommendations, I am pleased to inform you that your manuscript has been accepted in principle for publication in The EMBO Journal.

Before we can proceed with the next steps towards publication of your article, there is a minor comment regarding a typographical error (referee #2), which we kindly ask you to address in a final version of your manuscript.

In addition, there are a few more changes and corrections from the editorial side we need you to make in the final version of the manuscript:

- Please remove the Figures from the main manuscript file. Figures should only be uploaded separately as individual high-quality Figure files (as they already are). Their legends should remain in the manuscript, below the References lists.

- The reference format must be updated to the style used by EMBO Press (i.e., alphabetical list, with the names of the first 10 co-authors listed for each citation, followed by "et al."). For more information, please refer to our Author Guidelines: https://link.springer.com/journal/44318/submission-guidelines#cms-Reference-guidelines.

- Please provide in the Data Availability section of the revised manuscript the database, accession ID, and specific URL for the mass spectrometry data generated in this study. Please consider depositing these data in a structured database suitable for this type of data. All data should be publicly available at the time of publication.

- We kindly request that authors consider both actual and perceived competing interests. Please review our policy (https://link.springer.com/journal/44318/submission-guidelines#conflictsofinterest) and include a competing interests statement. Please name this section "Disclosure and competing interests statement" and place it after the Acknowledgements section.

- Please note that Figure panel callouts in the text should be listed sequentially.

- The Appendix file needs to be in PDF format; its title page should contain heading "Appendix for", followed by the manuscript's title (author and affiliation lists are not necessary), and a Table of Contents including page numbers for all listed items; nomenclature for the Appendix items should be "Appendix Figure S#" and "Appendix Table S#" throughout the manuscript and the Appendix file.

- The EV Figures should be removed from the Appendix PDF file and only be uploaded as individual Figure files (which is correctly done) with their legends remaining in the main manuscript file below the legends of the regular Figures.

- Please change heading "Materials and Methods" to "Methods".

- We noticed that your Reagents and Tools Table file includes two references; please move those to the main list of References in the manuscript file.

- Please note that EMBO press papers are accompanied online by:
A) a short (2 sentences) summary of the findings and their significance,
B) 2-5 short bullet points highlighting the key results, and
C) a synopsis image in .jpg or .png format that is exactly 550 pixels wide and 300 pixels high. Please note that all text needs to be legible at the final size.
Please upload this information along with your revised manuscript (the text for A and B should be provided in a separate Word file).

- During our standard Figure integrity checks, our team detected Figure reuse between Figure 1E and Figure EV2C. This is called out in the legend, however, the blot reuse is not called out. Please update the legends making sure that reuse of all elements, when necessary, is explicitly mentioned in the legends.

- The manuscript sections need to be named and ordered as follows: Title page - Abstract - Introduction - Results - Discussion - Methods - Data Availability - Acknowledgements - Disclosure and Competing Interests Statement - References - Figure Legends - main Tables (if there are any) - Expanded View Figure Legends (if there are any).

- Please also note that as part of the EMBO Press transparent editorial process, The EMBO Journal publishes online a Peer Review File along with each accepted manuscript. This File will be published in conjunction with your paper and will include the referee reports, your point-by-point responses and all pertinent correspondence relating to the manuscript. Your Author's Checklist will also be published at the end of the Peer Review File. Please let us know in case you want to remove any data or figures from your point-by-point responses before they are published as part of the Peer Review File. Retaining unpublished data in the Peer Review File means that these count as published and that the Peer Review File would need to be referenced in future publications. Please let the editorial office know in case you want to remove any data from this file (contact@embojournal.org).

I would like to thank you for informing us that you are interested in preparing an image/illustration for the front cover of our journal once a final decision has been made on the manuscript. We would be very happy to consider your candidate image(s)/illustration(s). Please feel free to contact my colleague Cornelius Schneider (c.schneider@embojournal.org), who currently coordinates the front cover pipeline for our journal, for more information.

We look forward to seeing a final version of your manuscript as soon as possible. Please let us know if you have any questions and use this link to submit your revision: https://emboj.msubmit.net/cgi-bin/main.plex.

Best regards,

Ioannis

Ioannis Papaioannou, PhD
Editor, The EMBO Journal
i.papaioannou@embojournal.org

Referee #2:

The authors have addresed my concerns from the primary reveiw of the manuscript very adequately. I appreciate the incoropartion of quantitative data (Kd values) and also the extension of the discussion with regard to the stability/foldedness of MHCI. I support publication and look forward to the manuscript being published.

Referee #3:

We appreciate the authors for their comprehensive replies and modifications.
Our main concern was that the role of UGGT1-mediated reglycosylation in MHC I quality control and PLC recruitment has already been established in previous studies. The authors have now acknowledged these prior findings and clarified that the principal advance of the present work lies in the biochemical reconstitution and mechanistic dissection of the MHC I recycling pathway.
We now understand that the novelty of this study lies not in demonstrating the importance of UGGT1-mediated reglucosylation itself, but in directly reconstituting the pathway and demonstrating that UGGT1-mediated reglucosylation enables calreticulin-dependent transfer of TAPBPR-bound MHC I to the tapasin-ERp57-containing PLC.
We appreciate the revisions made to the Introduction and Discussion sections to better distinguish the current study from previous work. Our concerns have been adequately addressed, and we have no further comments.
As a minor typographical correction, please change "$CaCl^2$" on page 19, line 10, to "$CaCl_2$."
Therefore, we recommend the acceptance of this manuscript.

All editorial and formatting issues were resolved by the authors.

2nd Revision - Editorial Decision **29th Jun 2026**

Dear Prof. Tampé,

Congratulations on an excellent manuscript! I am very pleased to inform you that it has been accepted for publication in The EMBO Journal. Thank you for comprehensively addressing the initially raised referees' concerns and our editorial requests for changes and corrections.

Your manuscript will be processed for publication by EMBO Press. It will be copy edited and you will receive page proofs prior to publication. Please note that you will be contacted by Springer Nature Author Services to complete licensing and payment information.

You may qualify for financial assistance for your publication charges - either via a Springer Nature fully open access agreement or an EMBO initiative. Check your eligibility: https://link.springer.com/journal/44318/how-to-publish-with-us

Should you be planning a Press Release on your article, please notify the production team via this form in order to coordinate publication and release dates: https://forms.cloud.microsoft/e/eH4A8JVP6g

If you have any questions, please do not hesitate to contact the Editorial Office. Thank you for your contribution to The EMBO Journal. Working with you has been a pleasure.

Best regards,

Ioannis

Ioannis Papaioannou, PhD
Editor, The EMBO Journal
i.papaioannou@embojournal.org

*****
Please note that it is The EMBO Journal policy for the transcript of the editorial process (containing referee reports and your response letters) to be published as an online supplement to each paper. If you should prefer removal of any referee-only figures included in the point-by-point response(s), e.g. because they may still be used for future publication or because they have been reproduced from published work by others, please do let us know immediately via response email.
More information is available here: https://link.springer.com/partners/embo-press/editorial-policies#Peer%20review

** Click here to be directed to your login page: https://emboj.msubmit.net

EMBO Press Author Checklist

Corresponding Author Name: Trowitzsch, S.; Tampé, R.	
Journal Submitted to: The EMBO Journal	
Manuscript Number: EMBOJ-2025-123404R	

USEFUL LINKS FOR COMPLETING THIS FORM
The EMBO Journal - Author Guidelines
EMBO Reports - Author Guidelines
Molecular Systems Biology - Author Guidelines
EMBO Molecular Medicine - Author Guidelines

Reporting Checklist for Life Science Articles (updated January
This checklist is adapted from Materials Design Analysis Reporting (MDAR) Checklist for Authors. MDAR establishes a minimum set of requirements in transparent reporting in the life sciences (see Statement of Task: 10.31222/osf.io/9sm4x). Please follow the journal's guidelines in preparing your
Please note that a copy of this checklist will be published alongside your article.

Abridged guidelines for figures
1. Data
The data shown in figures should satisfy the following conditions:
- the data were obtained and processed according to the field's best practice and are presented to reflect the results of the experiments in an accurate and unbiased manner.
- ideally, figure panels should include only measurements that are directly comparable to each other and obtained with the same assay.
- plots include clearly labeled error bars for independent experiments and sample sizes. Unless justified, error bars should not be shown for technical
- if n<5, the individual data points from each experiment should be plotted. Any statistical test employed should be justified.
- Source Data should be included to report the data underlying figures according to the guidelines set out in the authorship guidelines on Data

2. Captions
Each figure caption should contain the following information, for each panel where they are relevant:
- a specification of the experimental system investigated (eg cell line, species name).
- the assay(s) and method(s) used to carry out the reported observations and measurements.
- an explicit mention of the biological and chemical entity(ies) that are being measured.
- an explicit mention of the biological and chemical entity(ies) that are altered/varied/perturbed in a controlled manner.
- the exact sample size (n) for each experimental group/condition, given as a number, not a range;
- a description of the sample collection allowing the reader to understand whether the samples represent technical or biological replicates (including how many animals, litters, cultures, etc.).
- a statement of how many times the experiment shown was independently replicated in the laboratory.
- definitions of statistical methods and measures:
  - common tests, such as t-test (please specify whether paired vs. unpaired), simple χ2 tests, Wilcoxon and Mann-Whitney tests, can be unambiguously identified by name only, but more complex techniques should be described in the methods section;
  - are tests one-sided or two-sided?
  - are there adjustments for multiple comparisons?
  - exact statistical test results, e.g., P values = x but not P values < x;
  - definition of 'center values' as median or average;
  - definition of error bars as s.d. or s.e.m.

Please complete ALL of the questions below.
Select "Not Applicable" only when the requested information is not relevant for your study.

Materials

Newly Created Materials	Information included in the manuscript?	In which section is the information available? (Reagents and Tools Table, Materials and Methods, Figures, Data Availability Section)
New materials and reagents need to be available; do any restrictions apply?	Not Applicable	

Antibodies	Information included in the manuscript?	In which section is the information available? (Reagents and Tools Table, Materials and Methods, Figures, Data Availability Section)
For **antibodies** provide the following information:<br>- Commercial antibodies: RRID (if possible) or supplier name, catalogue number and or/clone number<br>- Non-commercial: RRID or citation	Yes	Reagents and Tools Table

DNA and RNA sequences	Information included in the manuscript?	In which section is the information available? (Reagents and Tools Table, Materials and Methods, Figures, Data Availability Section)
**Short novel DNA or RNA including primers, probes**: provide the sequences.	Not Applicable	

Cell materials	Information included in the manuscript?	In which section is the information available? (Reagents and Tools Table, Materials and Methods, Figures, Data Availability Section)
**Cell lines:** Provide species information, strain. Provide accession number in repository **OR** supplier name, catalog number, clone number, and/**OR** RRID.	Yes	Reagents and Tools Table
**Primary cultures:** Provide species, strain, sex of origin, genetic modification status.	Not Applicable	
Report if the cell lines were recently **authenticated** (e.g., by STR profiling) and tested for mycoplasma contamination.	Not Applicable	

Experimental animals	Information included in the manuscript?	In which section is the information available? (Reagents and Tools Table, Materials and Methods, Figures, Data Availability Section)
**Laboratory animals or Model organisms:** Provide species, strain, sex, age, genetic modification status. Provide accession number in repository **OR** supplier name, catalog number, clone number, **OR** RRID.	Not Applicable	
**Animal observed in or captured from the field:** Provide species, sex, and age where possible.	Not Applicable	
Please detail **housing and husbandry conditions**.	Not Applicable	

Plants and microbes	Information included in the manuscript?	In which section is the information available? (Reagents and Tools Table, Materials and Methods, Figures, Data Availability Section)
**Plants:** provide species and strain, ecotype and cultivar where relevant, unique accession number if available, and source (including location for collected wild specimens).	Not Applicable	
**Microbes:** provide species and strain, unique accession number if available, and source.	Not Applicable	

Human research participants	Information included in the manuscript?	In which section is the information available? (Reagents and Tools Table, Materials and Methods, Figures, Data Availability Section)
If collected and within the bounds of privacy constraints report on age, sex and gender or ethnicity for all study participants.	Not Applicable	

Core facilities	Information included in the manuscript?	In which section is the information available? (Reagents and Tools Table, Materials and Methods, Figures, Data Availability Section)
If your work benefited from core facilities, was their service mentioned in the acknowledgments section?	Not Applicable	

Design

Study protocol	Information included in the manuscript?	In which section is the information available? (Reagents and Tools Table, Materials and Methods, Figures, Data Availability Section)
If study protocol has been **pre-registered, provide DOI in the manuscript**. For clinical trials, provide the trial registration number **OR** cite DOI.	Not Applicable	
Report the **clinical trial registration number** (at ClinicalTrials.gov or equivalent), where applicable.	Not Applicable	

Laboratory protocol	Information included in the manuscript?	In which section is the information available? (Reagents and Tools Table, Materials and Methods, Figures, Data Availability Section)
Provide DOI OR other citation details if **external detailed step-by-step protocols** are available.	Not Applicable	

Experimental study design and statistics	Information included in the manuscript?	In which section is the information available? (Reagents and Tools Table, Materials and Methods, Figures, Data Availability Section)
Include a statement about **sample size** estimate even if no statistical methods were used.	Yes	Figure Legends, Materials and Methods
Were any steps taken to minimize the effects of subjective bias when allocating animals/samples to treatment (e.g. **randomization procedure**)? If yes, have they been described?	Not Applicable	
Include a statement about **blinding** even if no blinding was done.	Not Applicable	
Describe **inclusion/exclusion criteria** if samples or animals were excluded from the analysis. Were the criteria pre-established?<br><br>If sample or data points were omitted from analysis, report if this was due to attrition or intentional exclusion and provide justification.	Not Applicable	
For every figure, are **statistical tests** justified as appropriate? Do the data meet the assumptions of the tests (e.g., normal distribution)? Describe any methods used to assess it. Is there an estimate of variation within each group of data? **I**s the variance similar between the groups that are being statistically compared?	Not Applicable	

Sample definition and in-laboratory replication	Information included in the manuscript?	In which section is the information available? (Reagents and Tools Table, Materials and Methods, Figures, Data Availability Section)
In the figure legends: state number of times the experiment was **replicated** in laboratory.	Yes	Figure Legends
In the figure legends: define whether data describe **technical or biological replicates**.	Not Applicable	

Ethics

Ethics	Information included in the manuscript?	In which section is the information available? (Reagents and Tools Table, Materials and Methods, Figures, Data Availability Section)
Studies involving **human participants**: State details of **authority granting ethics approval** (IRB or equivalent committee(s), provide reference number for approval.	Not Applicable	
Studies involving **human participants**: Include a statement confirming that **informed consent** was obtained from all subjects and that the experiments conformed to the principles set out in the WMA Declaration of Helsinki and the Department of Health and Human Services Belmont Report.	Not Applicable	
Studies involving **human participants:** For publication of **patient photos**, include a statement confirming that consent to publish was obtained.	Not Applicable	
Studies involving experimental **animals**: State details of **authority granting ethics approval** (IRB or equivalent committee(s), provide reference number for approval. Include a statement of compliance with ethical regulations.	Not Applicable	
Studies involving **specimen and field samples:** State if relevant **permits** obtained, provide details of authority approving study; if none were required, explain why.	Not Applicable	

Dual Use Research of Concern (DURC)	Information included in the manuscript?	In which section is the information available? (Reagents and Tools Table, Materials and Methods, Figures, Data Availability Section)
Could your study fall under dual use research restrictions? Please check biosecurity documents and list of **select agents and toxins** (CDC): https://www.selectagents.gov/sat/list.htm	Not Applicable	
If you used a select agent, is the security level of the lab appropriate and reported in the manuscript?	Not Applicable	
If a study is subject to dual use research of concern regulations, is the name of the **authority granting approval and reference number** for the regulatory approval provided in the manuscript?	Not Applicable	

Reporting

The MDAR framework recommends adoption of discipline-specific guidelines, established and endorsed through community initiatives. Journals have their own policy about requiring specific guidelines and recommendations to complement MDAR.

Adherence to community standards	Information included in the manuscript?	In which section is the information available? (Reagents and Tools Table, Materials and Methods, Figures, Data Availability Section)
State if relevant guidelines or checklists (e.g., **ICMJE, MIBBI, ARRIVE, PRISMA**) have been followed or provided.	Not Applicable	
For **tumor marker prognostic studies,** we recommend that you follow the **REMARK** reporting guidelines (see link list at top right). See author guidelines, under 'Reporting Guidelines'. Please confirm you have followed these guidelines.	Not Applicable	
For **phase II and III randomized controlled trials**, please refer to the **CONSORT** flow diagram (see link list at top right) and submit the CONSORT checklist (see link list at top right) with your submission. See author guidelines, under 'Reporting Guidelines'. Please confirm you have submitted this list.	Not Applicable	

Data Availability

Data availability	Information included in the manuscript?	In which section is the information available? (Reagents and Tools Table, Materials and Methods, Figures, Data Availability Section)
Have **primary datasets** been deposited according to the journal's guidelines (see 'Data Deposition' section) and the respective accession numbers provided in the Data Availability Section?	Yes	Data Availability Section
Were **human clinical and genomic datasets** deposited in a public access-controlled repository in accordance to ethical obligations to the patients and to the applicable consent agreement?	Not Applicable	
Are **computational models** that are central and integral to a study available without restrictions in a machine-readable form? Were the relevant accession numbers or links provided?	Not Applicable	
If publicly available data were reused, provide the respective **data citations in the reference list**.	Not Applicable	
